# Supplementary material for: Outdoor environmental comfort evaluation for retail planning in a tropical business district using Integrated Environmental Modeller
Source: PLoS One. 2023 Mar 3;18(3):e0282106. doi: 10.1371/journal.pone.0282106 (PMC9983900; doi:10.1371/journal.pone.0282106)
Supplement: Attachment — Submitted filename: Response to Reviewers.pdf [file pone.0282106.s002.pdf]

PLoS One / Ref. No.: PONE-D-22-17882

Title: Outdoor environmental comfort evaluation for retail planning in a tropical business district using Integrated Environmental Modeller

Author: Po-Yen Lai, Wee Shing Koh, Harish Gopalan, Huizhe Liu, Dias Leong, Hyosoo Lee, Johnathan Goh, Jiun Yeu Lim, Jacob Ang, Gibert Peh, Gilbert Cher, Cheng Hui Eng, Jia Li Goh, Edmund Tan, James Tan

---

Dear Editor,

Thanks for your response. We also would like to thank the reviewers for their valuable comments. Our point-by-point replies for the journal requirements and reviewers' comments are shown as follows. The revised manuscript is resubmitted. Please consider for the publication on PLOS ONE.

Reply to Journal requirements

C1	Please ensure that your manuscript meets PLOS ONE's style requirements, including those for file naming. The PLOS ONE style templates can be found at https://journals.plos.org/plosone/s/file?id=wjVg/PLOSOne_formatting_sample_main_body.pdf and https://journals.plos.org/plosone/s/file?id=ba62/PLOSOne_formatting_sample_title_authors_affiliations.pdf
R1	Thanks for the suggestion. We've applied the format of the manuscript in accordance with the requirements of the journal strictly, including those for file naming.
C2	a) Please note that PLOS journals require authors to make all data necessary to replicate their study's findings publicly available without restriction at the time of publication. Please see our Data Availability policy at https://journals.plos.org/plosone/s/data-availability. As such, please make the full specific dataset used in this study available by either A) uploading the full dataset as supplementary information files, or B) including a URL link in your Data Availability Statement and Methods section to where the full dataset can be accessed.<br>b) Additionally, please note that PLOS ONE has specific guidelines on code sharing for submissions in which author-generated code underpins the findings in the manuscript. In these cases, all author-generated code must be made available without restrictions upon publication of the work. Please review our guidelines at https://journals.plos.org/plosone/s/materials-and-software-sharing#loc-sharing-code and ensure that your code is shared in a way that follows best practice and facilitates reproducibility and reuse.
R2	a) We want to change our description of "Data Availability" as:<br>"All the simulation conditions were listed in the manuscript. The CAD data used in simulations will be made available at the A*STAR Open Access Repository (https://oar.a-star.edu.sg/) upon acceptance of the manuscript. Registration of an authenticated access account at the A*STAR Open Access Repository site is required. The CAD data will be made freely available if the user is going to use the CAD data for the sole purpose to reproduce the results presented in this paper. Permission has to be sought if the CAD data is going to be used for any other purposes."<br>b) The software used in the manuscript has been licensed by Wizvision Pte Ltd (https://www.wizvision.com/contact-us/). Interested users purchase the Integrated Environmental Modeller software from the distributor.
C3	Thank you for stating the following in the Acknowledgments Section of your manuscript:<br>"This research is supported by A*STAR under its URBAN & GREENTECH OFFICE (UGTO) CONSULTANCY FUND (Grant No.: UGTO/CF/01). This development work was undertaken as a part of the project "Modeling of Air Flow,

	Thermal and Chemical Gas Dispersion Towards Next Generation Port (Tuas Maritime Hub)", funded by the Singapore Maritime Institute (SMI-2016-MA-04). The conclusions put forward reflect the views of the author alone, and not necessarily those of the funding institution"<br>We note that you have provided funding information that is not currently declared in your Funding Statement. However, funding information should not appear in the Acknowledgments section or other areas of your manuscript. We will only publish funding information present in the Funding Statement section of the online submission form.<br>Please remove any funding-related text from the manuscript and let us know how you would like to update your Funding Statement. Currently, your Funding Statement reads as follows:<br>"This research is supported by A*STAR under its URBAN & GREENTECH OFFICE (UGTO) CONSULTANCY FUND (Grant No.: UGTO/CF/01). This development work was undertaken as a part of the project "Modeling of Air Flow, Thermal and Chemical Gas Dispersion Towards Next Generation Port (Tuas Maritime Hub)", funded by the Singapore Maritime Institute (SMI-2016-MA-04). The conclusions put forward reflect the views of the author alone, and not necessarily those of the funding institution. The funders had no role in study design, data collection and analysis, decision to publish, or preparation of the manuscript.<br>Please include your amended statements within your cover letter; we will change the online submission form on your behalf.
R3	Thanks for the comment. In this case, we removed the "Acknowledgments section" in the manuscript and we confirmed that the current Funding Statement reads as follows: "This development work was undertaken as a part of the project "Modeling of Air Flow, Thermal and Chemical Gas Dispersion Towards Next Generation Port (Tuas Maritime Hub)", funded by the Singapore Maritime Institute (SMI-2016-MA-04). The conclusions put forward reflect the views of the author alone, and not necessarily those of the funding institution. The funders had no role in study design, data collection and analysis, decision to publish, or preparation of the manuscript." is correct.
C4	We note that Figure 1 in your submission contain [map/satellite] images which may be copyrighted. All PLOS content is published under the Creative Commons Attribution License (CC BY 4.0), which means that the manuscript, images, and Supporting Information files will be freely available online, and any third party is permitted to access, download, copy, distribute, and use these materials in any way, even commercially, with proper attribution. For these reasons, we cannot publish previously copyrighted maps or satellite images created using proprietary data, such as Google software (Google Maps, Street View, and Earth). For more information, see our copyright guidelines: http://journals.plos.org/plosone/s/licenses-and-copyright.<br>We require you to either (a) present written permission from the copyright holder to publish these figures specifically under the CC BY 4.0 license, or (b) remove the figures from your submission:<br>a. You may seek permission from the original copyright holder of Figure 1 to publish the content specifically under the CC BY 4.0 license.<br>We recommend that you contact the original copyright holder with the Content Permission Form (http://journals.plos.org/plosone/s/file?id=7c09/content-permission-form.pdf) and the following text:<br>"I request permission for the open-access journal PLOS ONE to publish XXX under the Creative Commons Attribution License (CCAL) CC BY 4.0 (http://creativecommons.org/licenses/by/4.0/). Please be aware that this license allows unrestricted use and distribution, even commercially, by third parties. Please reply and provide explicit written permission to publish XXX under a CC BY license and complete the attached form."<br>Please upload the completed Content Permission Form or other proof of granted permissions as an "Other" file with your submission.<br>In the figure caption of the copyrighted figure, please include the following text: "Reprinted from [ref] under a CC BY

	license, with permission from [name of publisher], original copyright [original copyright year].”<br>b. If you are unable to obtain permission from the original copyright holder to publish these figures under the CC BY 4.0 license or if the copyright holder’s requirements are incompatible with the CC BY 4.0 license, please either i) remove the figure or ii) supply a replacement figure that complies with the CC BY 4.0 license. Please check copyright information on all replacement figures and update the figure caption with source information. If applicable, please specify in the figure caption text when a figure is similar but not identical to the original image and is therefore for illustrative purposes only.<br>The following resources for replacing copyrighted map figures may be helpful:<br>USGS National Map Viewer (public domain): http://viewer.nationalmap.gov/viewer/<br>The Gateway to Astronaut Photography of Earth (public domain): http://eol.jsc.nasa.gov/sseop/clickmap/<br>Maps at the CIA (public domain): https://www.cia.gov/library/publications/the-world-factbook/index.html and https://www.cia.gov/library/publications/cia-maps-publications/index.html<br>NASA Earth Observatory (public domain): http://earthobservatory.nasa.gov/<br>Landsat: http://landsat.visibleearth.nasa.gov/<br>USGS EROS (Earth Resources Observatory and Science (EROS) Center) (public domain): http://eros.usgs.gov/#<br>Natural Earth (public domain): http://www.naturalearthdata.com/
R4	Thanks for pointing this out. We combined the **Figure 1(a)** and **(b)** and remove the copyrighted [map/satellite] images in **Figure 1(a)**. The amended **Figure 1** and the caption are as follows<br>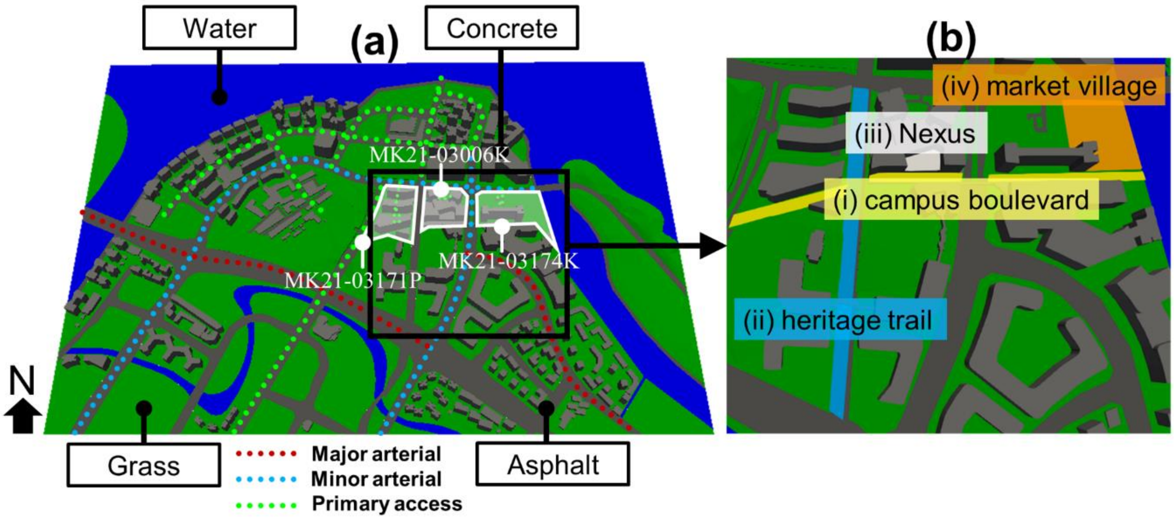<br>**Fig 1. (a)** The schematic illustration of the studied urban district in the north-east region of Singapore, including the material assignment of infrastructures and the categorization of traffic noise sources from main roads. White portions are the planned business park with the specific mukim lot numbers MK21-03171P, MK21-03006K and MK21-03174K. **(b)** The zoom-in feature of the study sites with high pedestrian traffic for the evaluation of comfort factors: (i) campus boulevard, (ii) heritage trail, (iii) Nexus, and (iv) market village.<br>The figure number has been updated accordingly in corresponding context. (Please see the document “Revised Manuscript with Track Changes” or “Manuscript”.)
C5	Regarding the potential copyright of figures 1, 5, 6, 7, and 8c please respond the following prompts:<br>A. For the above figures, were any shapefiles or basemaps obtained? If so, where were they obtained? Please provide any relevant links as well as any relevant copyright information.<br>B. For the above figures, were any external data utilized in the creation of these figures? If so, where were they obtained?

	Please inform us if the data was collected or produced by the authors. Please also provide any relevant links as well as any relevant copyright information.<br>C. Was any software (e.g. ArcGIS) used in the creation of these figures?
R5	A. No basemaps or shapefiles were used.<br>B. All the geometries were generated by the authors.<br>C. Commercial software, Revit and Rhino, were used to generate, report, and export the geometries for simulations. Simulations results were plotted with the open-source software, Paraview.

Reply to Reviewer #1's comments

C1	Include all the keywords inside the Abstract. "Tropical urban district", "Thermal comfort index", "Acoustic comfort index", "Environmental evaluation" and "retail planning" are not in the abstract.
R1	We thank the reviewer for the comment. The keywords are slightly revised as 「Retail planning, Integrated Environmental Modeller (IEM), Thermal comfort, Acoustic comfort, Tropical urban district」 and added into Abstract as follows.<br>Ln 16: 「…for a planned business urban district in Singapore for **retail planning** using…」<br>Ln 18: 「…called the **Integrated Environmental Modeller** (IEM). 」<br>Ln 22: 「…**thermal** and **acoustic comfort** acceptability indicators. 」<br>Ln 38-39: 「people working in or visiting the **tropical urban district**. 」
C2	Since coupled wind-thermal is one of main research, if possible the author might to explain more on definition or background what is coupled wind-thermal?
R2	The "wind-thermal" here is defined as the interaction between wind dynamics and heat transfer on the building or ground surfaces in the urban environment. "coupled" in the paper is mainly used to represent the connection between "wind-thermal model" and "noise propagation". For example, in the Ln 68「…coupled wind-thermal and noise propagation…」 To make it clearer, we add the definition of "wind-thermal" in the Ln 65-67 「in which the core physical model is the wind-thermal interaction between wind dynamics and heat transfer on building and ground surfaces in the urban environment. 」 and remove "coupled" in Ln 235.
C3	The author claim there is no studies on the coupled solar-wind-noise simulations, however there is model developed and used to study for this case? Seems like contradict. (on line 67 – 71).
R3	Thanks for pointing this out. The references [12, 21, 22, 23] are the different modules in IEM which was published for each physical schemes (i.e., solar radiation [12], wind dynamics [22, 23], and noise propagation [21]). The results of coupled solar-wind-noise simulations are not published yet.<br>To avoid misleading the readers, we amend the context and include few more current references on Ln 76-81 as 「…Integrated Environmental Modeller (IEM), has been developed to conduct coupled solar-wind-noise simulations in Singapore at different spatial scales from individual buildings to districts and regions. The IEM framework integrates environmental models, including solar radiation [12], wind dynamics [23, 24], and noise propagation [22], to captures the correlation between the physical models in simulations…」
C4	Line 442, reference defined the thresholds for thermal and acoustic comfort? Same goes to threshold for PTA and PNA.
R4	The comfort thresholds are not universal but are defined for different purposes and target matrices. We listed down few references, in which authors mentioned about "thermal comfort threshold", below for instance:<br>[Ref 4-1] Atmosphere 2018, 9, 108<br>[Ref 4-2] Procedia Engineering 2015, 2125-2131, 121<br>[Ref 4-3] Lancet Planet Health 2022, e301-e309, 6<br>In this work, we adopted the majority threshold for thermal and acoustic comfort, i.e., PTA = 0.5 and PNA = 0.5, to

determine whether majority of pedestrians accepts the thermal and acoustic conditions of the specific locations. The local thermal or acoustic comfort must be improved where PTA < 0.5 or PNA < 0.5. Then we claim the threshold used in this study is not universal in the latter context.

To make it clearer, we added the context on Ln 452-454 as 「…From different studies [Ref 4-1, Ref 4-2, Ref 4-3], other environmental comfort thresholds were introduced based on different environmental matrices for the specific purpose. 」

C5 Line 612, is there any reference to support author claim on blocking solar.

R5 Thanks for the comment. As referring [Ref 5-1, Ref 5-2, Ref 5-3, Ref 5-4], controlling solar radiation (blocking by shelter or tree canopy) can be seen as the most effective way to improve the outdoor thermal comfort. We added these references into the manuscript on Ln 681-682 as 「…This fact means that blocking solar irradiance is more efficient to improve $\overline{PTA}$ in open spaces that agree with the findings from [Ref 5-1, Ref 5-2, Ref 5-3, Ref 5-4]. …」

[Ref 5-1] Shashua-Bar, L., Ioannis, X.T., Milo, H. Passive cooling design options to ameliorate thermal comfort in urban streets of a Mediterranean climate (Athens) under hot summer conditions. Build. Environ. 2012, 57, 110–119.

[Ref 5-2] Cheung, P.K. & Jim, C.Y. Comparing the cooling effects of a tree and a concrete shelter using PET and UTCI. Build. Environ. 2018, 130, 49–61.

[Ref 5-3] Nasrollahi, N., Ghosouri, A., Khodakarami, J., Taleghani, M. Heat-Mitigation Strategies to Improve Pedestrian Thermal Comfort in Urban Environments: A Review. Sustainability 2020, 12, 10000.

[Ref 5-4] Assessment of outdoor air temperature with different shaded area within an urban university campus in hot-humid climate. Open Access. Zaki, S.A., Syahidah, S.W., Shahidan, M.F., (...), Hassan, M.Z., Daud, M.Y.M. 2020. Sustainability (Switzerland). 12(14), 5741, pp. 1-24

C6 Maybe the author can compile results from Figure 9, 10 ,11 and 12 in Table.

R6 Thanks for the comment. We added Table 4 that summarize the results from Figures 9, 10 ,11 and 12 as follows:

Table 4. The table summarizes the conditions to achieve $\overline{PTA}$ = 0.5 from **Error! Reference source not found.**(d), 10(d), 10(e), 11(d), 12(d), and 12(e). In each cell, the upper panel is the required shade percentage, and the upper panel is the required increasing wind speed. The cells in green represent that $\overline{PTA}$ ≥ 0.5 can be naturally achieved. The cells in red represent that cannot be achieved by only cannot be achieved by only increasing the wind speed in a reasonable range (< 10 m/s).

		campus boulevard site (i)	heritage trail site (ii)	Nexus site (iii)	market village site (iv)
sunny day	10:30	42 %<br>3.7 m/s	32 %<br>2.6 m/s		29 %<br>1.8 m/s
	13:30	68%<br>>10 m/s	68 %<br>>10 m/s	4.2 %<br>0.2 m/s	54 %<br>>10 m/s
cloudy day	10:30		6.7 %<br>0.3 m/s		17 %<br>0.8 m/s
	13:30		21 %<br>1.1 m/s		27.5 %<br>1.8 m/s

The corresponding context was added on Ln 615-617: 「…The overall conditions to achieve $\overline{PTA}$ = 0.5 from **Error! Reference source not found.**(d), 10(d), 10(e), 11(d), 12(d), and 12(e) are summarized in Table 4. 」 and on Ln 743-744: 「…According to Table 4 (the results from **Error! Reference source not found.**(d), **Error! Reference source not**

	**found.**(d), **Error! Reference source not found.**(e), **Error! Reference source not found.**(d), **Error! Reference source not found.**(d), and **Error! Reference source not found.**(e)) …」
C7	Here are several references the author might consider looking at:<br>[Ref 1] Field study of pedestrians' comfort temperatures under outdoor and semi-outdoor conditions in Malaysian university campuses Othman, N.E., Zaki, S.A., Rijal, H.B., Ahmad, N.H., Razak, A.A. 2021. International Journal of Biometeorology. 65(4), pp. 453-477<br>[Ref 2] Analysis of urban morphological effect on the microclimate of the urban residential area of Kampung Baru in Kuala Lumpur using a geospatial approach. Open Access. Zaki, S.A., Azid, N.S., Shahidan, M.F., (...), Ali, M.S.M., Yakub, F. 2020. Sustainability (Switzerland). 12(18),7301<br>[Ref 3] Assessment of outdoor air temperature with different shaded area within an urban university campus in hot-humid climate. Open Access. Zaki, S.A., Syahidah, S.W., Shahidan, M.F., (...), Hassan, M.Z., Daud, M.Y.M. 2020. Sustainability (Switzerland). 12(14),5741, pp. 1-24<br>[Ref 4] Effects of urban morphology on microclimate parameters in an urban university campus. Open Access. Zaki, S.A., Othman, N.E., Syahidah, S.W., (...), Shahidan, M.F., Saudi, A.S.M. 2020. Sustainability (Switzerland). 12(7), 2962<br>[Ref 5] Effects of roadside trees and road orientation on thermal environment in a tropical City. Open Access. Zaki, S.A., Toh, H.J., Yakub, F., (...), Ardila-Rey, J.A., Muhammad-Sukki, F. 2020. Sustainability (Switzerland). 12(3),1053
R7	Thanks for introducing the relevant references. We included four of them as follows:<br>[Ref 1] was cited on Ln 91 as: 「…To realize a fair thermal comfort assessment in a tropical climate, it is better to consider the results from local field studies in the existing thermal comfort index, e.g., [Ref 1, 33].…」<br>[Ref 3] was cited on Ln 682 as 「…This fact means that blocking solar irradiance is more efficient to improve $\overline{PTA}$ in open spaces that agree with the findings from [61, 62, 63, Ref 3]. …」<br>[Ref 4] was cited on Ln 719 as 「…The building shadows can directly improve the thermal comfort [Ref 4, 67]. …」<br>[Ref 5] was cited on Ln 722 as 「…and planting street trees with overhead coverage [54, 69, Ref 5]. …」
C8	The comments about the research conditions:<br>a) The reason of author choosing those specific research place?<br>b) The author might consider to include yearly temperature to support author chosen day.<br>c) Table 3, how the author choose the direction?<br>d) Line 318, reference on choosing dB value?<br>e) Line 366, the reason of choosing this specific initial temperature?<br>f) Line 667, the author can include the reasoning using this ideal condition.
R8	Thanks for the comments. Our responses are below:<br>a) The chosen place in study is a real planned district in Singapore (as mentioned in Abstract: Ln 16). The research results can help the planner to evaluate outdoor thermal and acoustic comfort. The selected studied sites in this paper are either the main pedestrian thoroughfares or gathering areas for outdoor community building and events with potential high pedestrian footfall where outdoor retail options are planned along or within these sites (see the context in Sec 2.1.1 Ln 149-157).<br>b) According to the Singapore's records of climate station in the past 30 years [39], the mean daily maximum air temperature is 32 ºC as defined "hot". To make it clear, we add the definition of hot day on Ln 200-202: 「…the hottest period (i.e., from March to June in which the air temperature is commonly higher than the mean daily maximum temperature of 32 ºC based on the climatological reference period between 1991 to 2020 [39]) …」<br>c) We adopted the BCA Green Mark's prevailing wind conditions [41], including mean speed and mean direction for Singapore, is based on the National Environment Agency's 18-year data at a reference height of 15.0 m. To make

Table 3 clearer, we amended the caption and table as follows:

Table 3. Climate conditions of wind applied to this paper. The expected values follow the Building and Construction Authority's (BCA) Green Mark [41] at the reference height of 15 m based on the National Environment Agency's 18-year data in Singapore.

**Date**	**mean speed (m/s)**	**mean direction**
Equinox (sunny day)	2.9	South
Solstice (cloudy day)	2.8	North-East

d) The value of 65 dB is suggested by WHO for the acoustic health purpose. To make it clear, we amend the context and add the corresponding reference on Ln 321-323 as: 「…Considering that the noise levels >65 dB could endanger the acoustic heath in urban environment [5], we assume that residents perceive traffic and construction noise annoyance level similarly at 65 dB …」

e) We adopted the BCA Green Mark's initial isothermal conditions of 30.0°C air temperatures to carry out the simulations. To make it clearer, we rewrote the corresponding context on Ln 373-374 as: 「…We adopted the initial isothermal conditions of 30.0°C air temperatures to carry out the simulations according to the BCA Green Mark standard [41].」

f) In this work, the reason for applying the ideal conditions is to propose the general parametric scope of thermal comfort improvement efficiently that can provide a quick guide for architects to plan the infrastructure and landscape combination during the scenario design stage. These ideal conditions are assumed that the solar radiation levels and wind speed have implicitly incorporated the effects of trees, shades, or outdoor fans. The exact details of surrounding environment, i.e., shades, trees and fans can be inversely designed based on the microclimate conditions, which is out of scope of this paper.

g) To make it clearer, we added the reason for ideal condition in the corresponding context on Ln 749-756 as: 「… Under the ideal conditions, we assumed that the solar radiation level and wind speed have implicitly incorporated the effects of trees, shades, or outdoor fans. The double parametric space in terms of solar radiation and wind speed provides a guideline for architects to quickly identify the optimal microclimate conditions for planning the infrastructure and landscape combination during the scenario design stage. The exact details of the surrounding environment, i.e., shades, trees and fans, can be inversely designed based on the optimal microclimate condition, which is out of the scope of this paper and requires further study. …」

C9

The comments for corrections:

a) Always start introduce the abbreviation first before use it. Line 64, "SOLWEIG" and "ENVI-met" not defined. Line 89, "WHO". Line 104, "3D".

b) Line 111, since author already defined IEM, just use IEM instead of full name.

c) Line 287, "Fk" referring for three things?

d) Figure 5, no arrow in (b).

R9

Thanks for the comments. Our responses are below:

a) We added "SOlar Long Wave Environmental Irradiance Geometry model (SOLWEIG)" in Ln 71-72, "World Health Organization (WHO)" in Ln 100, and "three dimensional (3D)" in Ln 115-116, respectively. ENVI-met is not the abbreviation but the name of the micro-climate software and the funded company.

b) We removed the full name of IEM in Ln 121.

	c) F_k is the angle factor form in k direction. To avoid misleading, we have rewritten the context in Ln 290-292: 「… Q_{Sk} (W/m^2) is the received shortwave radiation, $Q_{Lk}(W/m^2)$ is the received longwave radiation, and F_k is angle factor form in k direction…」<br>d) It's the typo. We corrected the caption in Figure 5: 「…The arrow in **(c)** denotes the wind direction.」<br>In addition, we also did the revisions in the manuscript as follows:<br>• "hourly average" to "hourly-averaged"<br>• Typo: we revised all "11:30" to "13:30"

Reply to Reviewer #2's comments

C1	This study does not include the novelty and significance of their research. This study is close to a technical report rather than a research paper. In addition, the acoustic comfort evaluation was simply conducted.
R1	We thank the reviewer to point out the comment that helps us to further improve the manuscript. We have carefully revised the **Abstract**, **Results and discussion**, and **Conclusion** sections to enhance the work's novelty and significance. Our bullet-point reply to this comment is addressed as follows:<br>• The novelty and significance of this research:<br>**Novelty:**<br>1. The first workflow of the solar-wind-noise coupled simulation rather than the conventional two-step solar-wind coupled simulations [12-15] is presented.<br>2. The spatial plots of environmental acceptability indicators to distinguish the different zones exposed to thermal or noise problems in the worst-case scenario are proposed. (in the revised paper)<br>3. The parametric contours of averaged thermal acceptability indicators for improving overall thermal comfort at the studied sites are proposed.<br>4. The guideline, considering thermal and acoustic comfort evaluation simultaneously for retail planning in a planned district, is given.<br>**Significance:**<br>1. The coupled physics (solar-wind-noise) simulations have been conducted for a planned business urban district in Singapore.<br>2. Using the simulation results, the spatial distribution of thermal and acoustic acceptability indicators can be divided into local zones with different comfort extents.<br>3. In the worst-case scenario, noise-affected zones are concentrated near the noise sources, and the thermal-affected zones are almost everywhere at the studied sites.<br>4. The area-averaged thermal acceptability indicators have been derived to quickly evaluate and improve the overall thermal comfort at each specific site for the high-level retail training.<br>5. According to the evaluation results, the work proposed the guideline of selecting the proper locality for outdoor retail planning, the strategy for improving outdoor environmental comfort, and excluding the difficult areas exposed thermal-noise problem if the thermal and acoustic comfort cannot be improved simultaneously.<br>• Acoustic comfort evaluation in this study:<br>We agree that the evaluation of acoustic comfort is simpler than thermal comfort in the previous manuscript. The area-averaged environmental acceptable indicators conveniently evaluate the overall thermal and acoustic comforts for the specific area. The overall acoustic comforts are more acceptable at each site than thermal comforts. However, the local acoustic comfort is always unacceptable in the region near the noise sources. The fact shows that spatial noise level variations should be considered in retail planning to define the uncomfortable acoustic zone. Therefore,

	we add the statistical error bar, extending from the averaged environmental acceptability indicators, to propose the most acceptable or unacceptable extent to each site's thermal and acoustic environmental conditions at different points in time. Moreover, we add the spatial plots of the distribution of environmental acceptability indicators to divide the area of each studied site into the zone affected by thermal or noise issues. According to the newly added contents, the complex regions encountering thermal and noise issues simultaneously are recommended to exclude outdoor retail activities if the thermal and acoustic issues cannot be improved simultaneously. This can further improve the evaluation and guideline for the retail planning for the planned district in Singapore. The corresponding content can be found in the revised manuscript's **Abstract**, **Results and discussion**, and **Conclusion** sections.
