# Supplementary material for: Outdoor environmental comfort evaluation for retail planning in a tropical business district using Integrated Environmental Modeller
Source: PLoS One. 2023 Mar 3;18(3):e0282106. doi: 10.1371/journal.pone.0282106 (PMC9983900; doi:10.1371/journal.pone.0282106)
Supplement: Attachment — Submitted filename: PONE-D-22-17882_R1_reviewer_comments.pdf [file pone.0282106.s003.pdf]

PLOS ONE

Outdoor environmental comfort evaluation for retail planning in a tropical business district using Integrated Environmental Modeller

--Manuscript Draft--

**Manuscript Number:**	PONE-D-22-17882R1
**Article Type:**	Research Article
**Full Title:**	Outdoor environmental comfort evaluation for retail planning in a tropical business district using Integrated Environmental Modeller
**Short Title:**	Environmental comfort evaluation for retail planning in a tropical business district
**Corresponding Author:**	Po-Yen Lai, Ph.D.<br>Institute of High Performance Computing<br>Singapore, Please select SINGAPORE
**Keywords:**	Retail planning; Integrated Environmental Modeller (IEM); Thermal comfort; Acoustic comfort; Tropical urban district
**Abstract:**	This research proposes a simulation-based assessment of outdoor thermal and acoustic comfort for a planned business urban district in Singapore for retail planning using a customized OpenFOAM-centric multi-physics environmental simulation platform called the Integrated Environmental Modeller (IEM). IEM was employed to simulate the coupled impacts of solar radiation on wind and air temperature and wind and air temperature effects on traffic noise propagation in the district on the equinox and solstice day of the hottest period. Using IEM simulation results, we computed the thermal and acoustic comfort acceptability indicators derived from local field studies' results. The spatial distribution of environmental comfort acceptability indicators in the worst-case scenario can be used to distinguish the zones exposed to thermal or noise influence. The noise-affected zones are near the main roads and overlap a part of the thermal-affected area. The thermal-affected area is almost everywhere in the studied sites in the worst-case scenario. Having outdoor retail spaces with both poor thermal and acoustic comfort is not recommended if the thermal and acoustic comfort cannot be improved simultaneously. For the high-level retail planning, a simplified parametric analysis considering solar irradiance blockage and wind speed enhancements, is provided. Considering the worst-case scenario, ≥50% thermal acceptability can be achieved by blocking 54%-68% solar irradiance among the pedestrian thoroughfares and the retail spaces. Coupled together, blocking the solar irradiance and enhancing the wind speed can further improve thermal comfort locally. These results can guide the retail mix (e.g., al fresco restaurants, pop-up kiosks etc.) near high footfall areas and provide reference for future plans combining landscape and infrastructure, (e.g., trees with shelter walkaways, green walls with outdoor ventilation fans etc.) taking into account the environmental acceptability of people working in or visiting the tropical urban district.
**Order of Authors:**	Po-Yen Lai, Ph.D.
	Wee Shing Koh
	Harish Gopalan
	Huizhe Liu
	Dias Leong
	Hyosoo Lee
	Johnathan Goh
	Jiun Yeu Lim
	Jacob Ang
	Gibert Peh
	Gilbert Cher

Powered by Editorial Manager® and ProduXion Manager® from Aries Systems Corporation

	Cheng Hui Eng
	Jia Li Goh
	Edmund Tan
	James Tan
**Response to Reviewers:**	Please see our bullet-point responses for all reviewer's comments in "Response to Reviewers".
**Additional Information:**	
**Question**	**Response**
**Financial Disclosure**<br><br>Enter a financial disclosure statement that describes the sources of funding for the work included in this submission. Review the submission guidelines for detailed requirements. View published research articles from *PLOS ONE* for specific examples.<br><br>This statement is required for submission and **will appear in the published article** if the submission is accepted. Please make sure it is accurate.<br><br>**Unfunded studies**<br>Enter: *The author(s) received no specific funding for this work.*<br><br>**Funded studies**<br>Enter a statement with the following details:<br>• Initials of the authors who received each award<br>• Grant numbers awarded to each author<br>• The full name of each funder<br>• URL of each funder website<br>• Did the sponsors or funders play any role in the study design, data collection and analysis, decision to publish, or preparation of the manuscript?<br>• **NO** - Include this sentence at the end of your statement: *The funders had no role in study design, data collection and analysis, decision to publish, or preparation of the manuscript.*<br>• **YES** - Specify the role(s) played.<br><br>* typeset	This research is supported by A*STAR under its URBAN & GREENTECH OFFICE (UGTO) CONSULTANCY FUND (Grant No.: UGTO/CF/01). This development work was undertaken as a part of the project “Modeling of Air Flow, Thermal and Chemical Gas Dispersion Towards Next Generation Port (Tuas Maritime Hub)", funded by the Singapore Maritime Institute (SMI-2016-MA-04). The conclusions put forward reflect the views of the author alone, and not necessarily those of the funding institution. The funders had no role in study design, data collection and analysis, decision to publish, or preparation of the manuscript.
**Competing Interests**<br><br>Use the instructions below to enter a competing interest statement for this	The authors have declared that no competing interests exist.

submission. On behalf of all authors, disclose any competing interests that could be perceived to bias this work—acknowledging all financial support and any other relevant financial or non-financial competing interests.<br><br>This statement is required for submission and **will appear in the published article** if the submission is accepted. Please make sure it is accurate and that any funding sources listed in your Funding Information later in the submission form are also declared in your Financial Disclosure statement.<br><br>View published research articles from *PLOS ONE* for specific examples.<br><br>**NO authors have competing interests**<br><br>Enter: *The authors have declared that no competing interests exist.*<br><br>**Authors with competing interests**<br><br>Enter competing interest details beginning with this statement:<br><br>*I have read the journal's policy and the authors of this manuscript have the following competing interests: [insert competing interests here]*<br><br>* typeset	
**Ethics Statement**<br><br>Enter an ethics statement for this submission. This statement is required if the study involved:<br><br>• Human participants<br>• Human specimens or tissue<br>• Vertebrate animals or cephalopods<br>• Vertebrate embryos or tissues<br>• Field research<br><br>Write "N/A" if the submission does not	N/A

Powered by Editorial Manager® and ProduXion Manager® from Aries Systems Corporation

require an ethics statement.<br><br>General guidance is provided below. Consult the submission guidelines for detailed instructions. **Make sure that all information entered here is included in the Methods section of the manuscript.**<br><br>**Format for specific study types**<br><br>**Human Subject Research (involving human participants and/or tissue)**<br>• Give the name of the institutional review board or ethics committee that approved the study<br>• Include the approval number and/or a statement indicating approval of this research<br>• Indicate the form of consent obtained (written/oral) or the reason that consent was not obtained (e.g. the data were analyzed anonymously)<br><br>**Animal Research (involving vertebrate animals, embryos or tissues)**<br>• Provide the name of the Institutional Animal Care and Use Committee (IACUC) or other relevant ethics board that reviewed the study protocol, and indicate whether they approved this research or granted a formal waiver of ethical approval<br>• Include an approval number if one was obtained<br>• If the study involved *non-human primates*, add *additional details* about animal welfare and steps taken to ameliorate suffering<br>• If anesthesia, euthanasia, or any kind of animal sacrifice is part of the study, include briefly which substances and/or methods were applied<br><br>**Field Research**<br><br>Include the following details if this study involves the collection of plant, animal, or other materials from a natural setting:<br>• Field permit number<br>• Name of the institution or relevant body that granted permission	
**Data Availability**	Yes - all data are fully available without restriction

Powered by Editorial Manager® and ProduXion Manager® from Aries Systems Corporation

Authors are required to make all data underlying the findings described fully available, without restriction, and from the time of publication. PLOS allows rare exceptions to address legal and ethical concerns. See the PLOS Data Policy and FAQ for detailed information.<br><br>A Data Availability Statement describing where the data can be found is required at submission. Your answers to this question constitute the Data Availability Statement and **will be published in the article**, if accepted.<br><br>**Important:** Stating 'data available on request from the author' is not sufficient. If your data are only available upon request, select 'No' for the first question and explain your exceptional situation in the text box.<br><br>Do the authors confirm that all data underlying the findings described in their manuscript are fully available without restriction?	
**Describe where the data may be found in full sentences. If you are copying our sample text, replace any instances of XXX with the appropriate details.**<br><br>• If the data are **held or will be held in a public repository**, include URLs, accession numbers or DOIs. If this information will only be available after acceptance, indicate this by ticking the box below. For example: *All XXX files are available from the XXX database (accession number(s) XXX, XXX.)*.<br>• If the data are all contained **within the manuscript and/or Supporting Information files**, enter the following: *All relevant data are within the manuscript and its Supporting Information files.*<br>• If neither of these applies but you are able to provide **details of access elsewhere**, with or without limitations, please do so. For example:	All the simulation conditions were listed in the manuscript. The CAD data used in simulations will be made available at the A*STAR Open Access Repository (https://oar.a-star.edu.sg/) upon acceptance of the manuscript. Registration of an authenticated access account at the A*STAR Open Access Repository site is required. The CAD data will be made freely available if the user is going to use the CAD data for the sole purpose to reproduce the results presented in this paper. Permission has to be sought if the CAD data is going to be used for any other purposes.

Data cannot be shared publicly because of [XXX]. Data are available from the XXX Institutional Data Access / Ethics Committee (contact via XXX) for researchers who meet the criteria for access to confidential data.<br><br>*The data underlying the results presented in the study are available from (include the name of the third party and contact information or URL).*<br>• This text is appropriate if the data are owned by a third party and authors do not have permission to share the data.<br><br>* typeset	
Additional data availability information:	Tick here if the URLs/accession numbers/DOIs will be available only after acceptance of the manuscript for publication so that we can ensure their inclusion before publication.

Powered by Editorial Manager® and ProduXion Manager® from Aries Systems Corporation

Manuscript Click here to access/download;Manuscript;Manuscript.docx

Outdoor environmental comfort evaluation for retail planning in a tropical business district using Integrated Environmental Modeller

Po-Yen Lai[1]*, Wee Shing Koh[1, 2], Harish Gopalan[1], Huizhe Liu[1], Dias Leong[3], Hyosoo Lee[3], Johnathan Goh[3], Jiun Yeu Lim[1], Jacob Ang[3], Gibert Peh[3], Gilbert Cher[3], Cheng Hui Eng[3], Jia Li Goh[3], Edmund Tan[3], James Tan[3]

[1]A*STAR Institute of High Performance Computing, 1 Fusionopolis Way, #16-16 Connexis, 138632, Singapore

[2]Department of Physics, National University of Singapore, 2 Science Drive 3, 117551, Singapore

[3]Smart District / New Estates 2 / Urban Design & Architecture Divisions, JTC Corporation, 8 Jurong Town Hall Road, 609434, Singapore

Abstract

This research proposes a simulation-based assessment of outdoor thermal and acoustic comfort for a planned business urban district in Singapore for retail planning using a customized OpenFOAM-centric multi-physics environmental simulation platform called the Integrated Environmental Modeller (IEM). IEM was employed to simulate the coupled impacts of solar radiation on wind and air temperature and wind and air

temperature effects on traffic noise propagation in the district on the equinox and solstice day of the hottest period. Using IEM simulation results, we computed the thermal and acoustic comfort acceptability indicators derived from local field studies' results. The spatial distribution of environmental comfort acceptability indicators in the worst-case scenario can be used to distinguish the zones exposed to thermal or noise influence. The noise-affected zones are near the main roads and overlap a part of the thermal-affected area. The thermal-affected area is almost everywhere in the studied sites in the worst-case scenario. Having outdoor retail spaces with both poor thermal and acoustic comfort is not recommended if the thermal and acoustic comfort cannot be improved simultaneously. For the high-level retail planning, a simplified parametric analysis considering solar irradiance blockage and wind speed enhancements, is provided. Considering the worst-case scenario, ≥50% thermal acceptability can be achieved by blocking 54%-68% solar irradiance among the pedestrian thoroughfares and the retail spaces. Coupled together, blocking the solar irradiance and enhancing the wind speed can further improve thermal comfort locally. These results can guide the retail mix (e.g., al fresco restaurants, pop-up kiosks etc.) near high footfall areas and provide reference for future plans combining landscape and infrastructure, (e.g., trees with shelter walkaways, green walls with outdoor ventilation fans etc.) taking into account the environmental acceptability of people working in or visiting the tropical

urban district.

Keywords: Retail planning, Integrated Environmental Modeller (IEM), Thermal comfort, Acoustic comfort, Tropical urban district.

*Electronic address: lai_po-yen@ihpc.a-star.edu.sg

1. Introduction

Over the past two decades, rapid urbanization in Singapore has affected human livability in outdoor thermal and acoustic environments. [1] Urban corridors and high-rise urban structures absorb the intense solar radiation from the sun in the day and radiate heat at night. This induces a higher urban air temperature than that in rural areas, producing thermal stress [2], i.e., urban heat island (UHI) [3, 4]. As modern architectures and population exponentially grows, urban transportation needs have also vastly increased. With high road density and heavy traffic, traffic noise accounts for up to 80% of whole noise sources in urban areas [5]. Thus, the urban area is significantly noisier than the rural area, i.e., urban noise island (UNI) [6, 7]. UHI and UNI coexist and seriously affect urban areas' societal life and health conditions [6]. Therefore, assessing thermal and acoustic environments for improving the urban livability of planned districts in Singapore is essential in urban planning and architectural design.

Microclimate variables (solar radiation, air temperature, and wind speed) and noise

propagation together characterize the thermal and acoustic environments in urban areas [8]. To conduct thermal and acoustic environmental studies and assessments, numerical simulation has been widely used [9]. Such simulations can provide detailed information of the physical variables of interest at any point within the domain at different scales. [10, 11] Considering the typical weather condition in Singapore: a sunny day in the tropics with low average wind speeds, buoyancy-driven turbulence induced by solar radiation is the most critical parameter that influences urban microclimate simulations [12, 13, 14, 15] in which the core physical model is the wind-thermal interaction between wind dynamics and heat transfer on building and ground surfaces in the urban environment. Meanwhile, wind and thermal shear also impact the traffic noise levels in dense canopies [16, 17]. Therefore, a coupled wind-thermal and noise propagation model is valuable to simulate and evaluate the thermal and acoustic environment in dense urban spaces for lower wind speed conditions [18]. While, by using existing simulators (e.g., Rayman [19], SOlar Long Wave Environmental Irradiance Geometry model (SOLWEIG) [20], and ENVI-met [21]), many environmental studies have been done on simulations of solar radiation, wind dynamics, and noise propagation separately or solar-wind at most, there were no reported studies on the coupled solar-wind-noise simulations to our best knowledge. Hence, a novel coupled solar-wind-noise modeling tool, i.e., Integrated Environmental Modeller (IEM), has been

developed to conduct coupled solar-wind-noise simulations in Singapore at different spatial scales from individual buildings to districts and regions. The IEM framework integrates environmental models, including solar radiation [12], wind dynamics [23, 24], and noise propagation [22], to captures the correlation of several physical schemes in simulations. The outputs of such microscale simulations enable further evaluation of thermal and acoustic comfort in the Singapore's urban environment.

To adequately evaluate thermal comfort in outdoor spaces, the human physiological thermoregulation mechanisms, subjective psychological perceptions, cultural backgrounds, and weather conditions should be taken into account [25, 26]. Several thermal comfort indices have been proposed in different scopes of application, including Physiological Equivalent Temperature (PET) [27], Standard Effective Temperature (SET) [28], Universal Thermal Climate Index (UTCI) [29], Predicted Mean Vote (PMV) [30], Thermal Sensation Vote (TSV) [31], to name a few. To realize a fair thermal comfort assessment in a tropical climate, it is better to consider the results from local field studies in the existing thermal comfort index, e.g., [32, 33]. Based on the statistical results from the field studies conducted in the Singapore's outdoor environment [33], such work proposed a correlation between TSV and operative temperature derived as a linear regression formula. TSV depends on the local microclimate factors and human physiological thermoregulation on the Singaporean

background. The corresponding thermal acceptability was further derived as a function of TSV using binary logistic regression to quantify the occupants' reaction to the thermal environment.

Researchers commonly recognized the noise level as the acoustic comfort index, e.g., World Health Organization (WHO) noise guideline [34]. Similar to thermal acceptability, the research team proposed a relationship between the noise acceptability and noise level to quantify the occupants' reaction to the environmental noise according to the field study conducted in construction site offices in Hong Kong [35]. In a modern urban area, the main component of environmental noise comes from road traffic [5]. Traffic noise modeling is essential to evaluate the acoustic comfort in urban spaces by calculating the noise mapping, which helps qualitatively identify the noise level at a receiver position due to traffic emission source. [36] Traffic noise modeling considers traffic volume (e.g., average vehicle speed), composition (e.g., proportion of large vehicles), road geometry, and absorption features of ground cover in the calculation. The Calculation of Road Traffic Noise (CRTN) [37] is the first systematic scheme developed to predict the noise level due to road traffic. The CRTN method has been applied to predict the traffic noise and validated experimentally in Singapore [22].

This research is the first to propose a simulation-based workflow for simultaneously assessing the thermal and acoustic environmental comforts of a planned urban district

in Singapore. We conducted solar-wind-noise simulations based on a three dimensional (3D) urban district modeled by architects on equinox and solstice day during the hottest period of Singapore weather. Material properties, terrains, building appearance with 3D geometric details, and the worst-case peak period road traffic noise sources were considered in simulations. Using the local survey-based sensation models [33, 35], we further evaluate the acceptability of thermal and acoustic comfort of pedestrian thoroughfares and the retail spaces based on outputs from IEM. Moreover, we perform the parametric study of the decreasing solar irradiance and the increasing wind speed to show room for improvement of outdoor environmental thermal comfort theoretically. The evaluation provides the guidance of high pedestrian footfall areas for retail and event planning (i.e., al fresco dining, popular pop-up retail kiosks with long queues, outdoor theme business community events, etc.) that considers thermal and acoustic comfort in the new business district. This workflow provides a reference framework for incorporating environmental comfort modeling into future landscape-infrastructure planning as part of early stage architectural design. In this paper, the methodologies, including numerical approaches and the corresponding theoretical basis, are described in Sec. 2. The numerical results, the analysis, and the discussion are presented in Sec. 3. Finally, the concluding remarks on the work are given in Sec. 4.

2. Methodology

2.1 Main features of Study areas and environmental conditions

2.1.1 Main features of the business district

The study area, as shown in Fig 1(a) is a coastal district in the north-eastern region of Singapore. The terrain is relatively flat, with an elevation of 15.6 m above sea level. A waterway crosses through the south part of the district. The district's planned business park is marked in white, and the various locations referred to by its respective mukim lot numbers. Fig 1(a) shows the computer-aided design (CAD) view of the urban district where the business park is in the region marked within the black box. Fig 1(b) represents the zoom-in feature of the marked region. For outdoor comfort evaluations, four study sites with high pedestrian or human traffic are marked in Fig 1(b), which are respectively (i) an east-west boulevard that connects the business park buildings to the waterfront ("campus boulevard"), (ii) a north-south trail that was converted from an old road to a pedestrian-only walkway ("heritage trail"), (iii) an open podium under a mid-rise office building ("Nexus"), and (iv) an outdoor waterfront plaza to hold gatherings and events for tenants and their customers or families ("market village"). The sites proposed in this study are either the main pedestrian thoroughfares, or gathering areas for outdoor community building and events. Taking advantage of the potential high pedestrian footfall, outdoor retail options are planned along or within these sites. For

example, al fresco restaurants and pop-up retail kiosks may be set up around these sites, where visitors spend their time on various shopping, leisure, and dining activities. Thermal and acoustic comfort assessments in this work can be useful for retail planning to ensure that the outdoor areas are comfortable and attractive for outdoor dining, waiting for takeaways, queuing and shopping.

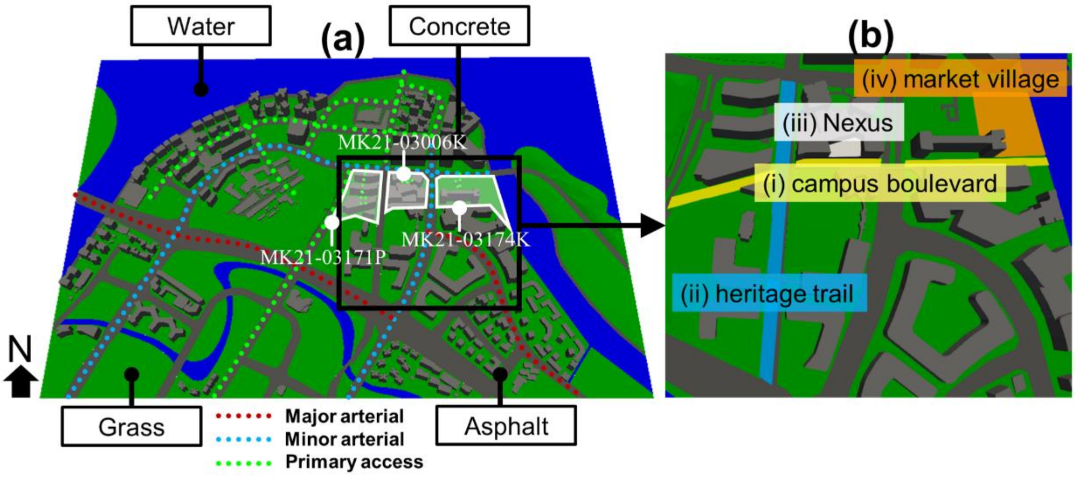

Fig 1. (a) The schematic illustration of the studied urban district in the north-east region of Singapore, including the four primary materials assigned to the 3D geometry and 3 different road categories. White portions are the planned business park with the specific mukim lot numbers MK21-03171P, MK21-03006K and MK21-03174K. **(b)** The zoom-in feature of the study sites with high pedestrian traffic for the evaluation of comfort factors: (i) campus boulevard, (ii) heritage trail, (iii) Nexus, and (iv) market village.

2.1.2 Environmental conditions for simulations

In Fig 1(a), the district's urban surfaces are grouped by materials, i.e., concrete, asphalt, grass, and water, which represents the appropriate boundary conditions for IEM coupled simulation. In the various simulations, buildings, roads, greenery, and water body were assigned concrete, asphalt, grass, water in which the surface of each object

has been defined own optical, thermal, acoustic properties. Table 1 gives the material properties used in this study.

The district's main roads classification is schematically represented by colored dashed lines in Fig 1(a). In Singapore, main roads are classified into five categories: expressway, major arterial, minor arterial, primary access, and local access from high to low traffic flow. This classification is purposed for the developer to establish the buffer requirements of surrounding buildings from the road [38] and used to define the traffic noise source in simulations. Table 2 shows that the categories used in this study are defined according to the numerical setup [22] in which the noise source is based on the hourly-averaged equivalent noise level during peak hours derived from measurements. Major arterials (i.e., 71 dB), shown in red, predominantly carry through traffic from one region to another, forming primary avenues of communication for urban traffic movements. Minor arterials (i.e., 70.2 dB), shown in blue, distribute traffic within the major residential and industrial areas. Primary accesses (i.e., 68.3 dB), shown in green, constitute the link between local accesses and arterial roads. In this study, the geometry description of the noise source was modeled as a finite line source at 1 meter above the center of the road surface.

Table 1 Material properties used in this paper.

Material	albedo	emissivity	heat absorptivity	sound absorptivity
asphalt	0.16	0.93	0.93	0.1
concrete	0.21	0.85	0.79	0.1
grass	0.39	0.98	0.61	0.1
water	0.14	0.96	0.86	0.1

Table 2 Road categories used in this paper.

Road Category	Hourly-averaged equivalent noise level (dB)
Major Arterial	71
Minor Arterial	70.2
Primary Access	68.3

The climate in Singapore is hot and humid, with a low average wind speed and abundant solar irradiation throughout the year. [39] The typical meteorological year (TMY) data [40] for Singapore was adopted as the data source in this study. Two representative days in the hottest period (i.e., from March to June in which the air temperature is commonly greater than the mean daily maximum temperature of 32 °C based on the climatological reference period between 1991 to 2020 [39]) in Singapore were selected for this study, which are equinox and solstice days respectively. The equinox day was a sunny day on 20 March, where the direct normal irradiance (DNI) mostly dominates over the diffused horizontal irradiance (DHI). Fig 2(a) shows the selected hourly-averaged climate data of sky longwave irradiance, direct normal irradiance, and diffuse horizontal irradiance on the equinox day, respectively. The solstice day was a cloudy day on 21 June. Fig 2(b) shows the corresponding metrological data in which the

radiation was diffusely dominated.

Considering that winds in Singapore are generally light, the daily average wind speed and direction were adopted in this study for simplicity. To make sure that the assessments in this study are certificated by the local policy, we adopted the Building and Construction Authority's (BCA) Green Mark scheme [41] as the wind conditions (including averaged wind speed and direction, reference height, and aerodynamic roughness length), which is the compulsory requirement for green building in Singapore. Table 3 shows the average daily wind speed and direction in selected days according to the BCA Green Mark scheme [41] to evaluate green buildings in Singapore, where the values are expected at the reference height of 15 m.

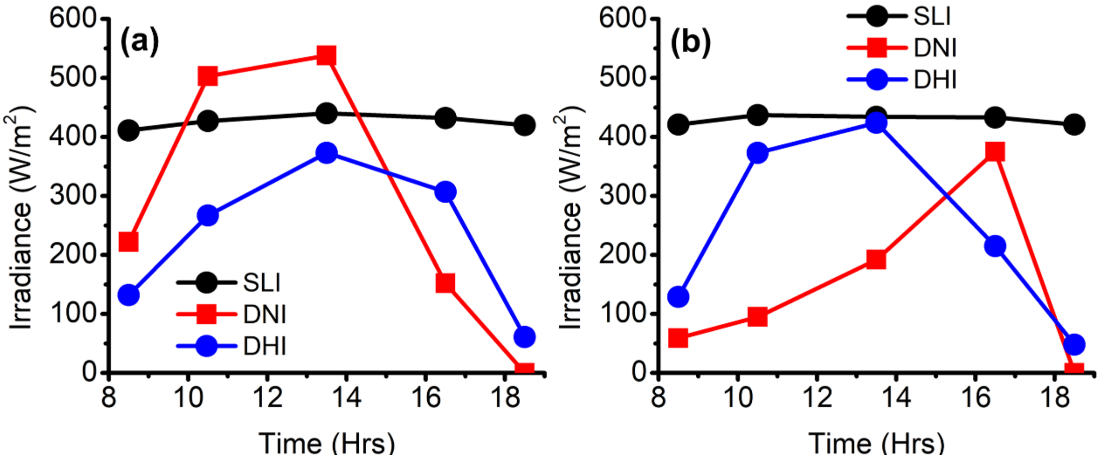

Fig 2 The selected hourly-averaged metrological data [40] of sky longwave irradiance (SLI), direct normal irradiance (DNI), and diffused horizontal irradiance (DHI) on **(a)** the equinox (sunny day) and **(b)** the solstice (cloudy day).

Table 3. Climate conditions of wind applied to this paper. The expected values follow the BCA Green Mark [41] at the reference height of 15 m based on the National

Environment Agency’s 18-year data.

Date	mean speed (m/s)	mean direction
Equinox (sunny day)	2.9	South
Solstice (cloudy day)	2.8	North-East

2.2 Integrated Environmental Modeller

The Integrated Environmental Modeller (IEM) [12, 22, 23, 24] has been developed as an integrated multi-physics urban microclimatic modeling tool for urban environment evaluation customized for the tropical climate. IEM can simulate the coupling between solar irradiance, wind flow, air temperature, and traffic noise propagation. Fig 3 shows the workflow of IEM. Two different methods are available for calculating solar irradiance: ray-tracing and discrete ordinates models. The ray-tracing approach was used for standalone solar irradiance studies, while the discrete ordinate models were used for wind-thermal computations to keep the computational cost low. Furthermore, the Perez all-weather sky model [12, 42] was incorporated as it has the best overall performance over a wide range of locations [43] and is one of the most suitable transposition models to predict solar irradiance for Singapore [44, 45]. Using the solar heat solver in IEM, the solar irradiance values on all surfaces in the studied system can be generated and passed to the aerodynamic simulation as input parameters.

The aerodynamic model in IEM was developed within OpenFOAM®, a finite-volume

computational fluid dynamics (CFD) platform. Buoyancy effects were modeled using the Boussinesq approximation. The steady-state Reynolds-averaged Navier-Stokes (RANS) method was used for turbulence modeling to reduce the computational cost [24]. The turbulence effect was modeled using Wray-Agarwal (WA) one-equation model [24, 46, 47]. The turbulent external flows around the buildings of concern were resolved by solving the RANS equation. The Monin-Obukhov similarity theory (MOST) [48] was applied to specify the boundary conditions for wind, temperature, and turbulent viscosity. [49] All building surfaces and ground were set as non-slip walls. Atmospheric boundary layer profile for neutral flow was used at the inlet of the computational domain. Surface-Energy-balance (SEB) model determines the heat transfer between the wind flow and the building surface. Solar shortwave radiation, thermal longwave radiation, and convective and convection heat transfer are the main mechanisms of the SEB model. The finite volume Discrete Ordinates Method (fvDOM) [50] was adopted to simulate the longwave radiation exchange between the urban surfaces and the sky. Using the aerodynamic model in IEM, the wind speed and air temperature values in the climate space can be generated and passed to noise simulation as input parameters.

The noise propagation model in IEM was developed based on the Calculation of Road Traffic Noise (CRTN) [51] coupled with the atmospheric refraction model [52] for

accessing meteorological effects. Once the turbulent wind flow has been simulated using the aerodynamic model in IEM, the calculated wind speed, lapse rate, and wind shear will be passed to the atmospheric refraction model. The atmospheric refraction effects on noise propagation are calculated and considered in the CRTN model. The noise level of the area of concern due to distance attenuation, ground absorption, screening, and site layout effects can be evaluated using the CRTN model. This approach [22] allows the adoption of a set of unstructured surface mesh to represent arbitrary 3D building geometry instead of just an extrusion of a building footprint.

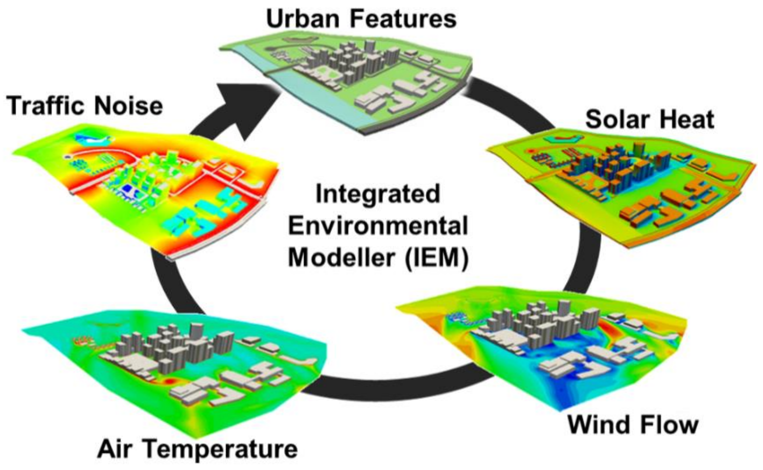

Fig 3. The workflow of coupled simulations using IEM.

2.3 Environmental comfort indices

Thermal indices have various degrees of dependence on environmental variables, including air temperature, wind speed, relative humidity, thermal radiation heat, and personal variables, including clothing, metabolic heat, mentality, and state of health. Because the empirical fits of these variables to derive thermal indices (e.g., predicted

mean vote) are complicated, a more straightforward measure can be more useful in practice. Thus, operative temperature (T_O) was adopted in this study as the thermal index derived from air temperature, mean radiant temperature, and wind speed. According to the standard from ISO 7726:1998 [53], the mathematical form of T_O (℃) is written as

$$T_O = \frac{T_{air}\sqrt{10U}+T_{MRT}}{\sqrt{10U}+1}, \quad (1)$$

where T_{air} (℃) is air temperature, U (m/s) is wind speed, and T_{MRT} (℃) is mean radiant temperature. By definition [54], T_{MRT} (℃) is the effective temperature and relates to the radiation received from all the incoming angles and can be written as

$$T_{MRT} = \sqrt[4]{\sum_k \frac{F_k}{\sigma}\left[\left(1-\alpha_p\right)Q_{Sk} + \varepsilon_p Q_{Lk}\right]} - 273.15. \quad (2)$$

where σ is Stefan-Boltzmann constant, $\alpha_p = 0.3$ is the effective albedo value for a person, and $\varepsilon_p = 0.9$ is the effective emissivity for a person. Q_{Sk} (W/m^2) is the received shortwave radiation, $Q_{Lk}(W/m^2)$ is the received longwave radiation, and F_k is angle factor form in k direction. In this study, the evaluation of T_{MRT} is on the pedestrian level (i.e., 1.5 m apart from the ground).

To reveal the relationships between environment/personal variables and the human thermal comfort sensation, ASHRAE [31] defined the unitless index as the 7-point scale (–3 for cold, –2 for cool, –1 for slightly cool, 0 for neutrality, 1 for slightly warm, 2 for

warm, and 3 for hot) called thermal sensation vote (TSV). Considering the average metabolic and clothing conditions in Singapore, a linear relationship [33] between TSV and T_O was derived as

$$TSV = 0.356T_O - 10.21. \quad (3)$$

According to the on-site survey in Singapore [33], the relationship between the percentage of thermal acceptability (PTA) and TSV was achieved using binary logistical regression, i.e.,

$$PTA = \frac{\exp(-1.118TSV+2.853)}{1+\exp(-1.118TSV+2.853)}. \quad (4)$$

In this study, Eq 4 was adopted to represent human acceptability with outdoor thermal conditions directly.

Noise intensity is another factor affecting human acceptability with outdoor environmental conditions. Unlike thermal comfort, the noise level is usually the only metric to define human acoustic comfort. According to the published literature [35], the logistic regression model of noise acceptance has been derived from on-site surveys and measurements in Hong Kong. Similar to Eq 4, the relationship between the percentage of noise acceptability (PNA) and noise level (L) (dB) can be written as

$$PNA = \frac{\exp(-0.55L+34.5)}{1+\exp(-0.55L+34.5)}. \quad (5)$$

Eq 5 is derived according to the equivalent continuous noise level sourced from the

construction site and measured in the construction office, where the acceptable noise level is 65 dB [35]. According to the guideline from Sustainable Mobility Initiative for Local Environment (SMILE) [5], traffic noise can be regarded as the primary sound source in this study. From the National Environment Agency (NEA) [55] report in Singapore, the maximum acceptable value of traffic noise level is about 67 dB averaged over an hour. However, people's exposure to noise levels above 65 dB can cause severe health problems [5]. Considering that the noise levels >65 dB could endanger the acoustic heath in urban environment [5], we assume that residents perceive traffic and construction noise annoyance level similarly at 65 dB. Eq 5 was applied to predict the percentage of noise acceptability for traffic noise.

To evaluate the percentage of environmental acceptability within a specific region, the area-weighted average factors ($\overline{PTA}$ and $\overline{PNA}$) over the pedestrian's way can be defined

$$\overline{PTA} = \frac{1}{A_{tot}} \int_{A_{tot}} PTA \, dS, \quad (6)$$

$$\overline{PNA} = \frac{1}{A_{tot}} \int_{A_{tot}} PNA \, dS, \quad (7)$$

where dS is the area differential element and A_{tot} is the total area of the specific region. These two factors can facilitate summarizing the population the percentage of specific regions for urban planning and architectural design.

2.4 Study procedures

The procedures of this study can be summarized as shown in Fig 4. First, as discussed in Sec 2.1, the TMY weather data related to solar irradiance, the typical wind speed and direction from BCA, and the 3D STL model for the area of concern were set for input to conduct a coupled simulation for the day and time of interest. Next, radiative and thermal material properties were assigned to each group of urban surfaces (concrete, grass, roads and water body) of the 3D STL model. IEM would then automatically generate meshes for the physical simulations. As discussed in Sec 2.2, the received shortwave irradiance (Q_S) mapping on the 3D model at a specific day and time was generated by the solar solver in IEM. Once Q_S values on the surface of 3D model have been calculated, Q_S will be taken into account in SEB model to determine the heat transfer boundary condition. Based on the heat transfer boundary condition, the meteorological quantities, air temperature (T_{air}), wind speed (U), and received longwave radiation (Q_L) were computed by the aerodynamic solver in IEM. The noise solver would calculate the noise level (L) in IEM, where U is considered in the noise simulation. Finally, as discussed in Sec 2.3, the corresponding thermal indices (T_O, T_{MRT}, and TSV) and environmental comfort acceptability (PTA and PNA) would be evaluated using Eqs 1-5, respectively.

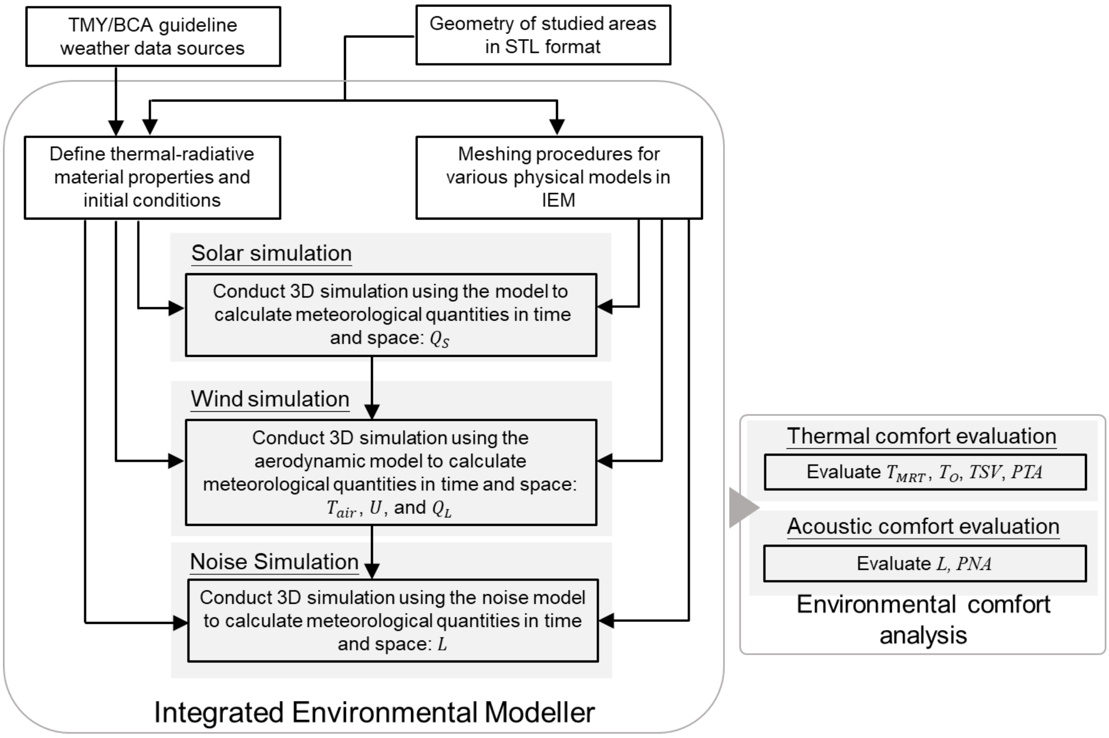

Fig 4. The flowchart represents the detailed research procedures in this study.

3. Results and discussion

3.1 Three-dimensional coupled simulations of the urban district

The size of the studied district is 2518 m × 1762 m × 84 m. For solar and noise simulations, the district's extent in STL format defines the computational domain. The meshes were generated uniformly for the 3D geometries. The resolution of the mesh is 2 meters, and the total mesh number is about 2.1 million. In wind simulations, the size of the computational domain must be greater than the original size of the district for setting corresponding inlet atmospheric boundary conditions. As discussed in Sec 2.1, the inlet wind condition is assigned according to the 2019 [41] shown in Table 3.

According to the wind directions at different times, the computational domain sizes are 44226 m × 30976 m × 605 m for the south inlet wind direction (i.e., the sunny day) and 22057 m × 30976 m × 605 m for the north-east inlet wind direction (i.e., the cloudy day). For generating the mesh for wind simulations, snappyHexMesh utility in OpenFOAM® has been used to discretize the computational domain. The total cell count of the computational domain is 17 million and consists of a maximum mesh resolution of 8 m away from the buildings and a minimum resolution of 2 m in the region of the building. The vertical resolution is 1 m. According to the Davenport-Wieringa roughness classification [56] and the BCA Green Mark standard [41], the aerodynamic roughness of inflow and ground surfaces were selected as 0.03 and 1.0, respectively. We adopted the initial isothermal conditions of 30.0°C air temperatures to carry out the simulations according to the BCA Green Mark standard [41].

In this section, we present physical quantities computed using IEM on the sunny day at 10:30 and on the cloudy day at 16:30, in which the solar radiation is the most direct dominant in each day. Fig 5 shows the simulated physical quantities on the sunny day at 10:30. Fig 5(a) is the solar irradiance mapping on the surfaces. The shadows of the buildings are on the west side due to the solar orientation in the morning. The air temperature and wind speed contours at the pedestrian level are shown in Figs 5(b)-(c), respectively. The wind profile (Fig 5(c)) is determined by the inflow wind direction,

topography, and material properties. It can be seen in Fig 5(b) that the hot spots are in the regions with abundant solar radiation ($Q_s > 600 W/m^2$) and low wind speed ($U <$ 0.5m/s). Fig 5(d) illustrates the 3D traffic noise map of the district. According to the traffic noise sources defined in Fig 1(a) and Table 2, the color in the middle of the road is significantly darker, implying the roads have a higher noise level. This noise mapping at the district comes from calculating the noise propagation from the source and the noise reduction due to obstruction, distance attenuation, ground absorption, and noise barrier screen. With such noise mapping and Eq 5, we can further evaluate the acoustic comfort in areas with high pedestrian traffic during peak hours and discuss this in Sec 3.2.

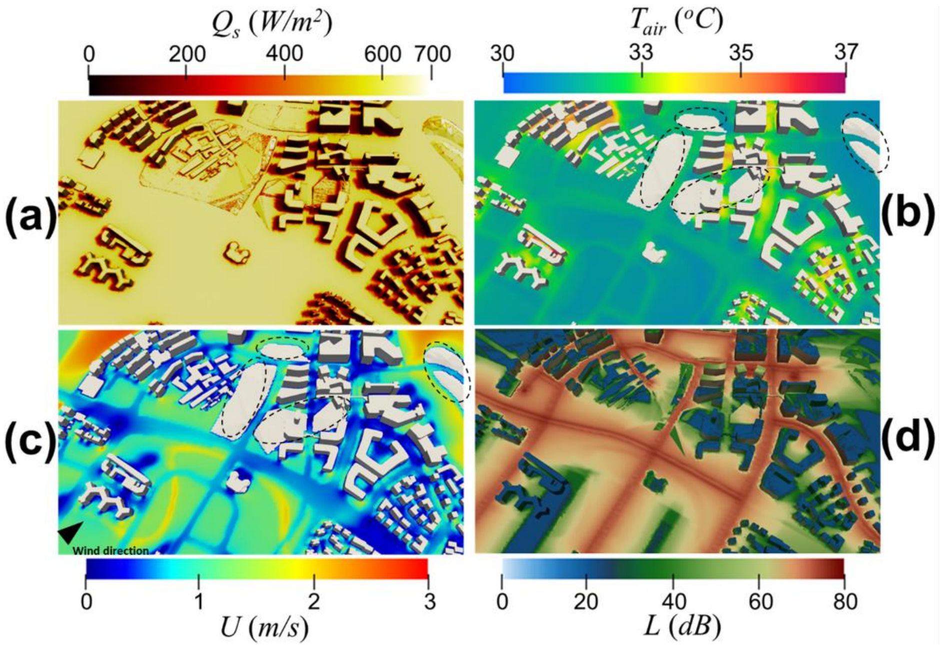

Fig 5. On the sunny day at 10:30, the figure shows **(a)** the solar irradiance mapping on the surface of the district, **(b)** air temperature as well as **(c)** wind speed contours at pedestrian level cut-plane (i.e., 1.5m) of the district, and **(d)** the noise level mapping on the surface of the district. The regions circled in **(b)** and **(c)** indicate that the terrain

is higher than the 1.5m pedestrian level cut-plane. The arrow in **(c)** denotes the wind direction.

On the other hand, Fig 6 shows the simulated physical quantities on the cloudy day at 16:30. The shadows of the buildings are on the east side due to the solar orientation in the afternoon. Fig 6(a) also shows that the amplitude of overall solar irradiance is relatively weak compared with that shown in Fig 5(a). It is reflected in the air temperature profile shown in Fig 6(b), where a decrease in global solar radiation decreases the overall air temperature. Under the different inflow directions, the wind speed profile at that time is revealed in Fig 6(c). Fig 6(d) shows the corresponding noise mapping. There is no noticeable difference between Figs 5(d) and 6(d) because the temperature gradient and wind shear impact on traffic noise propagation of both cases is relatively low for the area and tropical climate under study.

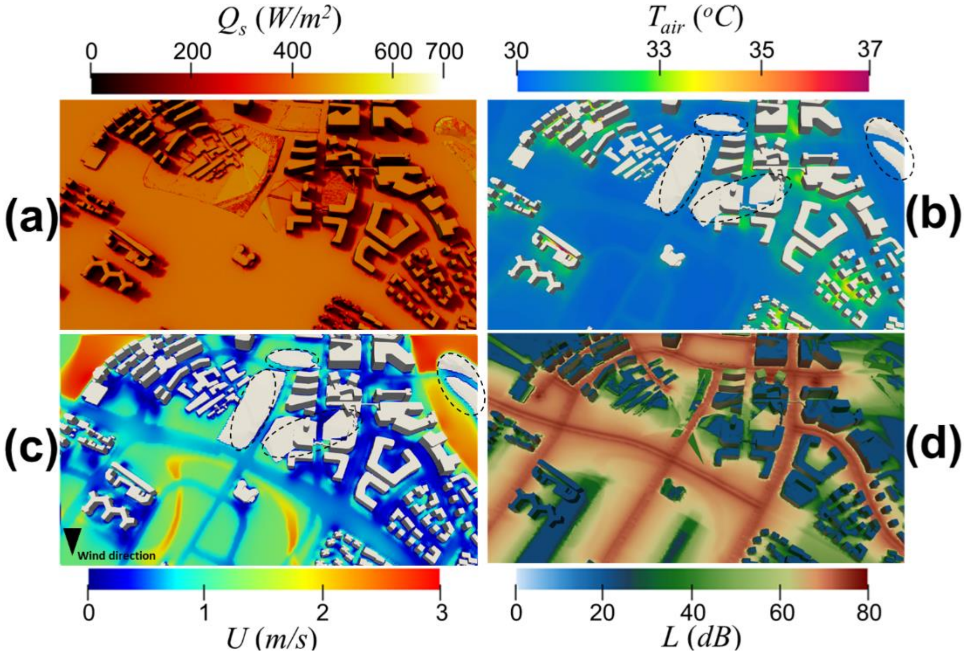

Fig 6. On the cloudy day at 16:30, the figure shows **(a)** the solar irradiance mapping

on the surface of the district, **(b)** air temperature as well as **(c)** wind speed contours at pedestrian level (i.e., 1.5-m) of the district, and **(d)** the noise level mapping on the surface of the district. The regions circled in **(b)** and **(c)** indicate that the terrain is higher than the 1.5m pedestrian level cut-plane. The arrow in **(c)** denotes the wind direction.

3.2 Evaluation and analysis of thermal comfort on the study sites

To further evaluate the environmental comfort in the area with high pedestrian traffic are marked in Fig 1(b), Fig 7 shows the thermal comfort index (i.e., TSV) and acoustic comfort index (i.e., L) contours at the pedestrian level (i.e., 1.5 m). The TSV results are calculated using Eq 3 based on the simulation results shown in Fig 5(a)-(c) and Figs 6(a)-(c). Fig 7(a) shows that TSV >2 in more than 80% of the area. The outdoor environment is considered hot on the sunny day at 10:30. In contrast, TSV on a cloudy day at 16:30, shown in Fig 7(c), is relatively low (<1) for >60% of the area of interest. The high TSV generally happens due to high solar irradiance, and due to the weak wind flow that is usually associated with a low wind speed of ≤ 2 m/s (see Fig 6(c)). Thus, in Figs 7(a) and 7(c), TSV hotspots occur in areas with great solar exposure and inadequate natural ventilation, strongly affected by street geometry, building orientation, wind direction, and sun position. For example, in Fig 7(a), the hotspot can be observed in the south area of the site (iii), where it is unshaded and lacks the speed for adequate permeability of natural ventilation on the sunny day 10:30. In contrast, in Fig 7(c), the

reduction of TSV in the same place is because solar irradiance, sun position, and wind direction change on the cloudy day 16:30. The contour of the noise level (L) shown in Figs 7(b) and 7(d) are similar, indicating that the effect of wind and thermal shear are weak for the area of interest. These results in Figs 7(b) and 7(d) are derived from the IEM outputs as shown in Figs 5(d) and 6(d).

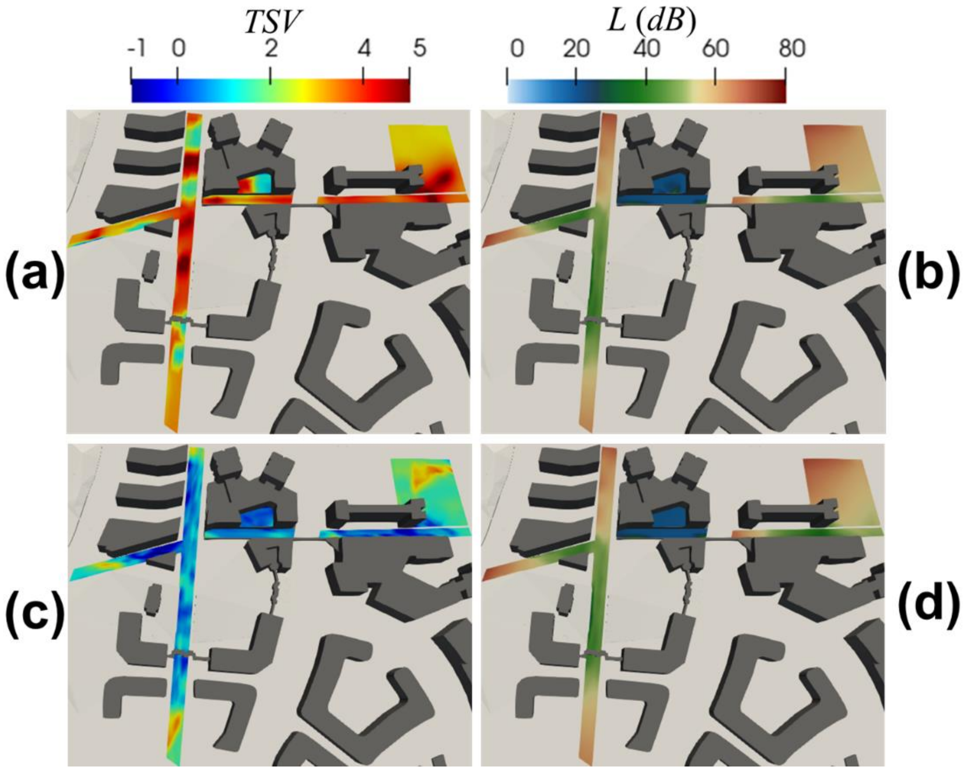

Fig 7. According to the simulation results shown in Fig 5 and Fig **6**, the figure shows contours of comfort indices of **(a)** TSV and **(b)** noise level at the sunny day 10:30, and **(c)** TSV and **(d)** noise level at the cloudy day 16:30.

Environmental acceptability as a people-centric way to evaluate thermal comfort for architecture and urban design, compared to using typical thermal comfort metrics (such as TSV) is also evaluated here. Fig 8(a) shows the relationship between the percentage of thermal acceptability (PTA) and TSV calculated following Eq 4. Similarly, Fig 8(b)

shows the relationship between the percentage of noise acceptability (PNA) and noise level according to Eq 5. The thresholds for thermal and acoustic comfort metrics are defined as 50% of the population feeling acceptable at TSV = 2.3 and L = 62.6 dB, respectively. The qualified evaluation of environmental comfort at a specific location is achievable based on whether the PTA and PNA are over 0.5. It is noted that the threshold (PTA = 0.5 and PNA = 0.5) used in this study is an estimate of the feelings of the simple majority rather than the universal definition. From different studies [57, 58, 59], other environmental comfort thresholds were introduced based on different environmental matrices for the specific purpose.

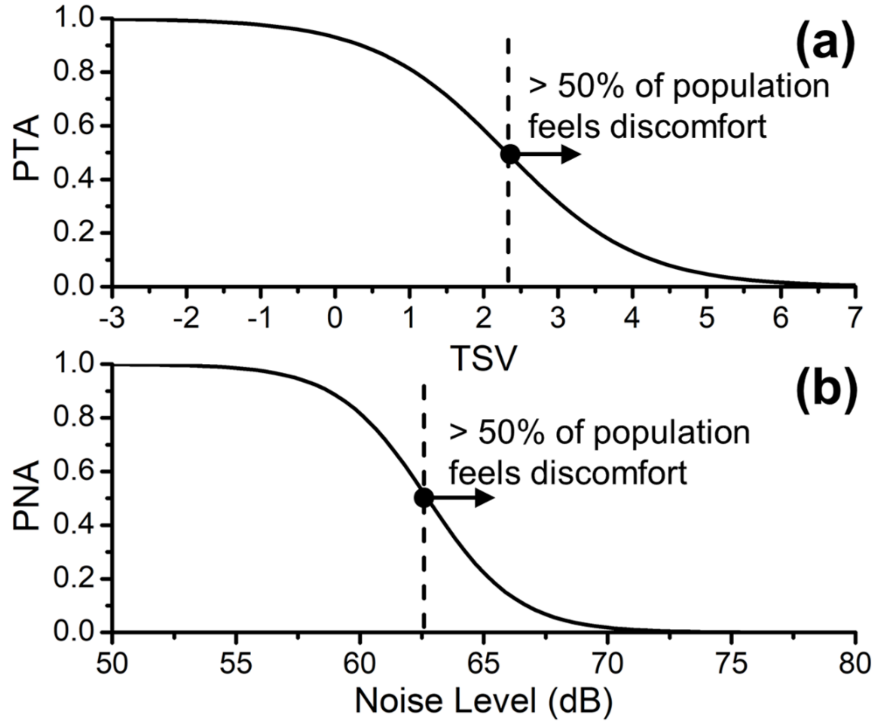

Fig 8. shows **(a)** the relationship between the percentage of thermal acceptability (PTA) and thermal sensation vote (TSV) obtained from Eq 4, and **(b)** the relationship between the percentage of noise acceptability (PNA) and noise level obtained from Eq 5, respectively. The dashed lines denote the index threshold (PTA = 0.5 and PNA = 0.5) of that over 50% population feels discomfort (i.e., the right area pointed by the

arrow).

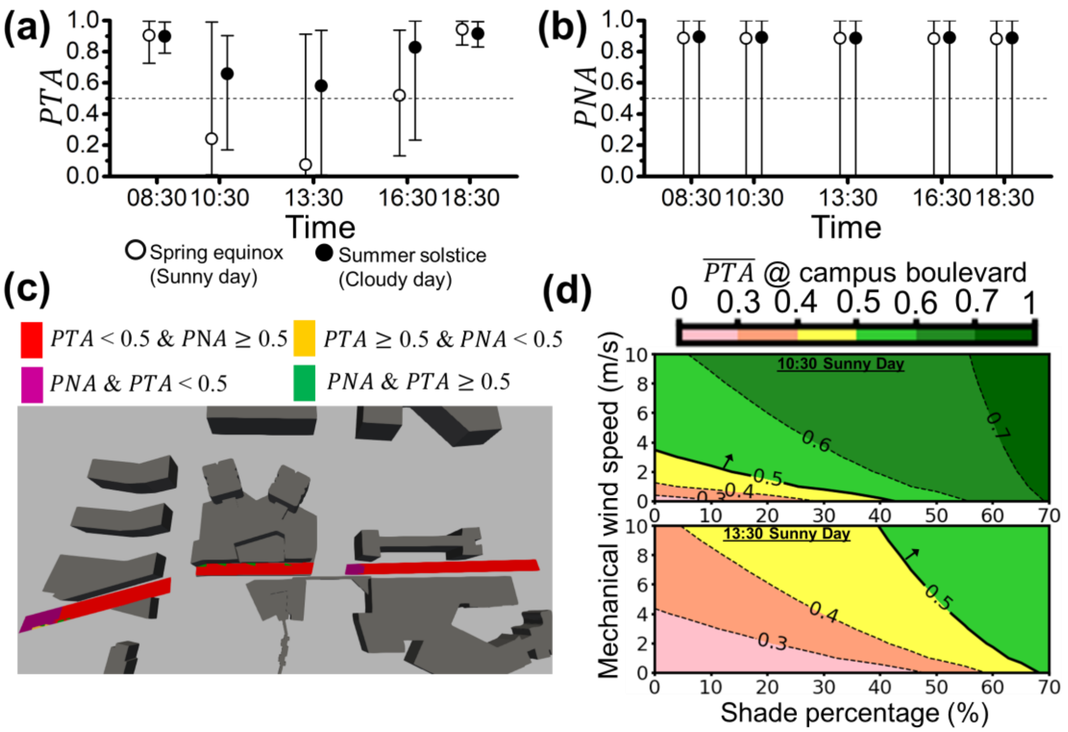

Fig 9. The area-averaged **(a)** PTA and **(b)** PNA of the campus boulevard (site (i)) for both sunny (open circles) and cloudy (filled circles) days are represented with open and filled circles (i.e., $\overline{PTA}$ and $\overline{PNA}$), respectively. The error bars extended from the area-averaged PTA or PNA (circles) represent the local maximum and local minimum PTA or PNA on the campus boulevard. The environmental acceptability threshold of 0.5 is denoted by the dotted line (i.e., $PTA =$ 0.5 or $PNA =$ 0.5). **(c)** The spatial plot of PTA and PNA above or below the threshold of 0.5 for campus boulevard at 13:30 on the sunny day (worst case) is illustrated. **(d)** The contour plot shows the potential improvement of $\overline{PTA}$ with the increase in shade and/or wind speed at 10:30 and 13:30 on the sunny day for the campus boulevard.

From Figs 9-12, we evaluate the environmental comfort at each site (i, ii, iii, and iv) within the area with high pedestrian traffic are marked in Fig 1(a). Fig 9(a) shows the area-averaged PTA (i.e., $\overline{PTA}$) of the sunny day and the cloudy day on the east-west boulevard. The cloudy day has higher $\overline{PTA}$s than the sunny day during the daytime, consistent with the trend of solar irradiance shown in Fig 2. The error bars illustrate the

huge variation between local maximum and minimum PTA at different locations along the campus boulevard. The local variation of PTA strongly depends on the extent of the shade projected by the surrounding buildings at that hour of interest. There are two points in time (10:30 and 13:30) that $\overline{PTA}$ is below 0.5 on the sunny day and the corresponding minimum PTA is very close to 0. We expect these two points to be under a relatively high global solar irradiance, and >50% of people are uncomfortable with the thermal environment. In contrast, Fig 9(b) shows that the area-averaged PNA (*i.e.* $\overline{PNA}$) is almost always close to a constant (~0.9) that far exceeds the baseline ($\overline{PNA}$ = 0.5) all the time during peak hours, even though the local minimum PNA is close to 0. In Fig 9(c), the spatial distribution of PTA and PNA at 13:30 on the sunny day (worst case) is illustrated. It is obvious that most of the area along the campus boulevard is expected to be thermally uncomfortable with $PTA < 0.5$ (red and purple zones), and the spaces, where $PNA < 0.5$, are concentrated near the two road junctions (i.e., traffic noise sources defined in Fig 1(a)), which overlaps with a part of the $PTA < 0.5$ region, marked in purple. The small zones with, $PTA < 0.5$ and $PNA > 0.5$; and; $PTA > 0.5$ and $PNA > 0.5$ along the bottom edge of the purple zone with $PTA < 0.5$ are marked in amber and green, respectively. This indicates that traffic noise level is always acceptable by most people even at peak hour traffic except for people who stay in the small region directly affected by the traffic noise. Local noise mitigation

measures are required in such the areas if open air retail spaces are planned in the purple and amber regions, where $PNA < 0.5$.

For improving overall environmental comforts on the specific site for high-level retail planning, the averaged indicators (i.e., $\overline{PTA}$ and $\overline{PNA}$) rather than local values (i.e., PTA and PNA) will be further analyzed. As compared to $\overline{PNA}$ shown in Fig 9(b), the improvement of $\overline{PTA}$ is the primary objective to be achieved by architectural solutions. We provide a simplified parametric analysis to improve thermal comfort at the hot times while $\overline{PTA}$ <0.5 (i.e., 10:30 and 13:30 of the sunny day) by decreasing solar irradiance and increasing the wind speed from the simulation results, and other conditions held constant. Tuning the two parameters may be done using setting up shelters to block the incoming solar radiation and adding ventilation fans to increase the local wind speed under ideal conditions. The contour plot in Fig 9(d) shows the results of the improvement of $\overline{PTA}$ by decreasing the solar irradiance and increasing the wind speed. Increasing shade percentage (i.e., decreasing solar irradiance) and increasing wind speed are set from 0 to 70% and 0 to 10 m/s, respectively. The increasing wind speed of 10 m/s is the maximum boost provided by typical outdoor ventilation fans [60]. The solid lines in Fig 9(d) indicate the threshold of $\overline{PTA}$ =0.5 to separate the region with $\overline{PTA}$ >0.5 (green colors) and the region with $\overline{PTA}$ <0.5 (warm colors). The intersections of the solid line and vertical and horizontal axes represent the minimum

requirements for the adopting the single way (only increasing wind speed and only increasing shade percentage) to achieve $\overline{PTA}$ =0.5. At 10:30 of the sunny day (the upper panel of Fig 9(d)), $\overline{PTA}$ =0.5 can be achieved by increasing 3.7 m/s in wind speed or blocking 42 % of solar irradiance. The corresponding value of wind speed and shade percentage on the solid line also denotes the minimum coupling mechanism criterion to achieve $\overline{PTA}$ = 0.5. For instance, we can improve $\overline{PTA}$ to 0.5 by increasing 2 m/s in wind speed and blocking 15% of solar irradiance simultaneously. In the lower panel of Fig 9(d), it is observed that the further increase in wind speed and shade percentage is required to reach the $\overline{PTA}$ baseline under a higher global solar irradiance at 13:30 of the sunny day as compared to that at any other time. $\overline{PTA}$ = 0.5 can be achieved by blocking 68 % of solar irradiance but cannot be achieved by only increasing the wind speed of <10 m/s.

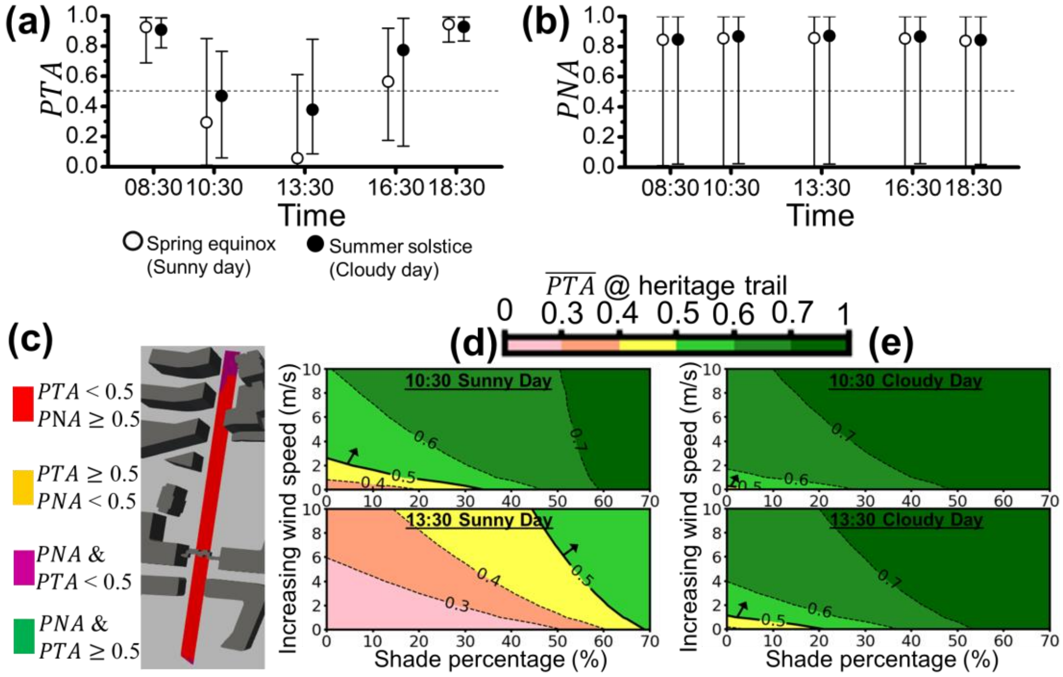

Fig 10. The area-averaged **(a)** PTA and **(b)** PNA of the heritage trail (site (ii)) for both sunny (open circles) and cloudy (filled circles) days are represented with open and filled circles (i.e., $\overline{PTA}$ and $\overline{PNA}$), respectively. The error bars extended from the area-averaged PTA or PNA (circles) represent the local maximum and minimum PTA or PNA on the heritage trail. The environmental acceptability threshold of 0.5 is denoted by the dotted line (i.e., $PTA = 0.5$ or $PNA = 0.5$). **(c)** The spatial plot of PTA and PNA above or below the threshold of 0.5 for heritage trail at 13:30 on the sunny day (worst case) is illustrated. The contour plot shows the potential improvement of $\overline{PTA}$ with the increase in shade and/or wind speed at 10:30 and 13:30 on **(d)** the sunny day, and **(e)** the cloudy day for the heritage trail.

Fig 10(a) and 10(b) show the area-averaged $\overline{PTA}$ and $\overline{PNA}$ of the sunny day and the cloudy day along the heritage trail. Unlike Fig 9(a), Fig 10(a) shows that $\overline{PTA}$s are less than 0.5 with the corresponding minimum $PTA \cong 0$ at 10:30 and 13:30 of the cloudy day. In Fig 10(b), PTAs are between 0.84 and 0.88, slightly less than that shown in Fig 9(b), but the corresponding minimum PNAs are close to 0 all the time. Fig 10(c) shows that most of the area of the heritage trail is exposed to the thermal problem with $PTA <$

0.5 (red and purple zones) at 13:30 on sunny day. At the same time, the space overlapped the area with $PNA < 0.5$ (purple zones) is located at the part of the north end of the heritage trail. The green ($PTA < 0.5$ and $PNA > 0.5$) and amber ($PTA > 0.5$ and $PNA > 0.5$) zones are almost none existed. Fig 10(c)-(d) show the improvement of $\overline{PTA}$ via two ways on the sunny and cloudy day. At 10:30 of the sunny day (the upper panel of Fig 10(c)), using increasing 2.6 m/s in wind speed or blocking 32 % of solar irradiance can reach $\overline{PTA}$ = 0.5. As can be seen in the lower panel of Fig 10(c), $\overline{PTA}$ = 0.5 can be achieved by blocking at least 68% of the incoming solar irradiance at 13:30 of the cloudy day, but the same $\overline{PTA}$ value cannot be achieved by only increasing the wind speed of $<$ 10 m/s. On the other hand, as shown in Fig 10(d), we can improve $\overline{PTA}$ to reach 0.5 via adding 6.7% shade percentage or increasing 0.3 m/s in wind speed at 10:30 of the cloudy day. $\overline{PTA}$ = 0.5 can be achievable by adding 21% shade percentage or increasing 1.1 m/s in wind speed at 13:30 of the cloudy day.

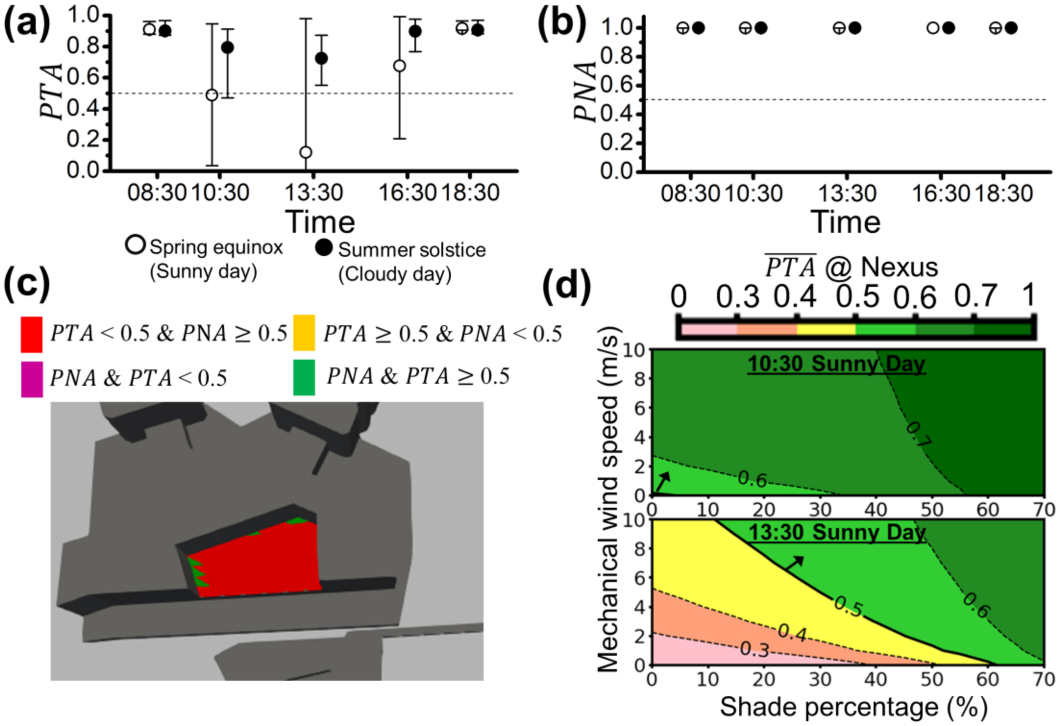

Fig 11. The area-averaged **(a)** PTA and **(b)** PNA of Nexus (site (iii)) for both sunny (open circles) and cloudy (filled circles) days are represented with open and filled circles (i.e., $\overline{PTA}$ and $\overline{PNA}$), respectively. The error bars extended from the area-averaged PTA or PNA (circles) represent the local maximum and local minimum PTA or PNA on Nexus. The environmental acceptability threshold of 0.5 is denoted by the dotted line (i.e., $PTA =$ 0.5 or $PNA =$ 0.5). **(c)** The spatial plot of PTA and PNA above or below the threshold of 0.5 for Nexus at 13:30 on the sunny day (worst case) is illustrated. **(d)** The contour plot shows the potential improvement of $\overline{PTA}$ with the increase in shade and/or wind speed at 10:30 and 13:30 on the sunny day for Nexus.

Fig 11(a)-(b) show the area-averaged $\overline{PTA}$ and $\overline{PNA}$ of the sunny day and the cloudy day at the Nexus. In Fig 11(a), $\overline{PTA}$s are higher than that shown in Fig 9(a) and Fig 10(a), especially on the cloudy day. However, the minimum PTA is still close to 0 on the sunny day, e.g., at 13:30 (worst case). From Fig 11(b), $\overline{PNA}$ is always close to 1 with almost zero variation that 100% of people satisfy the acoustic environment all the

time during peak hours. The spatial feature of PTA and PNA at 13:30 on the sunny day as shown in Fig 11(c) can be seen that the most of area is with $PTA < 0.5$ marked in red, few scattered areas with $PTA \geq 0.5$ are marked in green, and almost no area is with $PNA > 0.5$. Fig 11(d) shows the improvement of $\overline{PTA}$ via two strategies on the sunny day. At 10:30 of the sunny day (the upper panel of Fig 11(d)), merely using increasing 0.2 m/s in wind speed or blocking 4.2 % of solar irradiance can reach $\overline{PTA}$ = 0.5. In the lower panel of Fig 11(d), the baseline of $\overline{PTA}$ can be achievable by blocking 62% of solar irradiance at 13:30 of the sunny day but still be unachievable by solely increasing the wind speed.

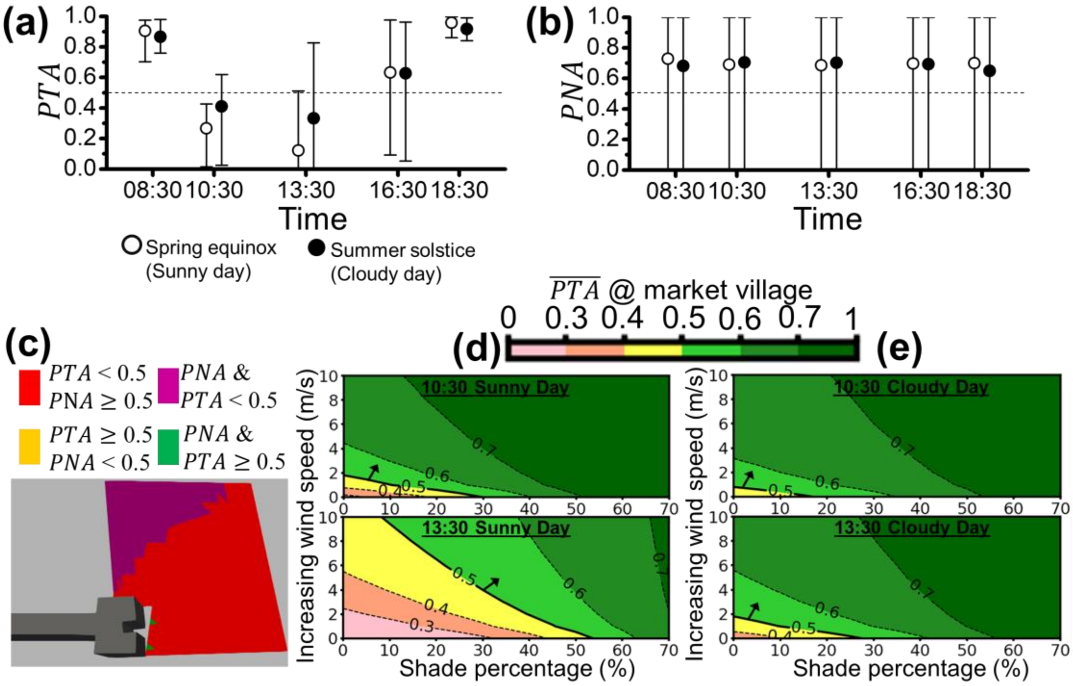

Fig 12 The area-averaged **(a)** PTA and **(b)** PNA of the market village (site (iv)) for both sunny (open circles) and cloudy (filled circles) days are represented with open and filled circles (i.e., $\overline{PTA}$ and $\overline{PNA}$), respectively. The error bars extended from the area-averaged PTA or PNA (circles) represent the local maximum and minimum PTA or PNA on the campus boulevard. The environmental acceptability

threshold of 0.5 is denoted by the dotted line (i.e., $PTA =$ 0.5 or $PNA =$ 0.5). **(c)** The spatial plot of PTA and PNA above or below the threshold of 0.5 for market village at 13:30 on the sunny day (worst case) is illustrated. The contour plot shows the potential improvement of $\overline{PTA}$ with the increase in shade and/or wind speed at 10:30 and 13:30 on **(d)** the sunny day, and **(e)** the cloudy day for the market village.

Figs 12(a)-(b) show the area-averaged $\overline{PTA}$ and $\overline{PNA}$ with the corresponding variation of the sunny day and the cloudy day at the market village. Similar to the results (see Fig 9(a)), $\overline{PTA}$s are less than 0.5 and the corresponding minimum $PTAs$ are close to 0 at 10:30 and 13:30 of the sunny and cloudy day as shown in Fig 12(a). It can be observed that the maximum PTAs at 10:30 and 13:30 of the sunny and cloudy day are much less than those elsewhere. On the other, in Fig 12(b), $\overline{PNA}$s are between 0.69 and 0.73, slightly less than that shown in the results from Figs 9(b), 10(b), and 11(b). The corresponding minimum $PNAs$ are close to 0 all the time. Fig 12(c) shows that the zone with $PTA < 0.5$ (red and purple) is almost everywhere and a part of space overlapped with $PNA < 0.5$ (purple) appears beside the noise sources. Figs 12(d)-(e) show the improvement of $\overline{PTA}$ via two strategies on the sunny and cloudy day. At 10:30 of the sunny day (the upper panel of Fig 10(d)), using increasing 1.8 m/s in wind speed or blocking 29 % of solar irradiance, we can achieve $\overline{PTA}$ = 0.5. In the lower panel of Fig 12(d), $\overline{PTA}$ = 0.5 can be achieved at 13:30 of the sunny day by blocking 54 % of solar irradiance. On the other hand, as shown in Fig 10(e), we can improve $\overline{PTA}$ to reach 0.5 via adding a 17% shade percentage or increasing 0.8-m/s in wind

speed at 10:30 of the cloudy day. $\overline{PTA}$ = 0.5 is achievable via adding 27.5% shade percentage or increasing 1.8-m/s wind speed at 13:30 of the cloudy day. The overall conditions to achieve $\overline{PTA}$ = 0.5 from Figs 9(d), 10(d), 10(e), 11(d), 12(d), and 12(e) are summarized in Table 4.

Table 4. The table summarizes the conditions to achieve $\overline{PTA}$ = 0.5 from Figs 9(d), 10(d), 10(e), 11(d), 12(d), and 12(e). In each cell, the upper panel is the required shade percentage, and the upper panel is the required increasing wind speed. The cells in green represent that $\overline{PTA}$ $\geq$ 0.5 can be naturally achieved. The cells in red represent that cannot be achieved by only cannot be achieved by only increasing the wind speed in a reasonable range (< 10 m/s).

		campus boulevard<br>site (i)	heritage trail<br>site (ii)	Nexus<br>site (iii)	market village<br>site (iv)
sunny day	10:30	42 %<br>3.7 m/s	32 %<br>2.6 m/s		29 %<br>1.8 m/s
	13:30	68%<br>>10 m/s	68 %<br>>10 m/s	4.2 %<br>0.2 m/s	54 %<br>>10 m/s
cloudy day	10:30		6.7 %<br>0.3 m/s		17 %<br>0.8 m/s
	13:30		21 %<br>1.1 m/s		27.5 %<br>1.8 m/s

3.3 Discussion

From Fig 9-12, $\overline{PTA}$ and $\overline{PNA}$ directly represent the overall human acceptance of outdoor environmental conditions in a specific area. The variation of PTA and PNA are affected by the local environmental conditions. At each time, the maximum or minimum PTA and PNA represent the most acceptable or unacceptable extent locally for thermal and acoustic comfort in different studied sites, respectively. The

results (Figs 9(b), 10(b), 11(b), and 12(b)) show that $\overline{PNA}$s do vary slightly (between 0.69 and 1) for the five different time slots due to the different wind and air temperature conditions for all the four sites in the business district. The site with more surrounding buildings, which act as acoustic barriers, has higher $\overline{PNA}$. Thus, the site (iii) (Nexus) has the highest $\overline{PNA}$ (= 1) among four sites and the site (iv) (market village) has the lowest $\overline{PNA}$ (~ 0.7). Although $\overline{PNA}$s is in the range between 0.7 and 1, the corresponding minimum PNA is close to 0 except for site (iii) (Nexus). From the results (Figs 9(c), 10(c), and 12(c)), the local zones with a small PNA are always near the noise sources. Among four sites in this study, the north-west region of site (iv) is exposed to the most serious traffic noise because this region is surrounded by noise sources without any acoustic barriers, like buildings. The straightforward way to improve the acoustic comfort by constructing local acoustic barriers to protect pedestrians from the traffic noise. However, the acoustic barriers will block airflow transport, further endangering thermal comfort. Thus, outdoor retail activities would not be recommended in the purple zones ($PNA < 0.5$ and $PTA < 0.5$) as shown in Figs 9(c), 10(c), and 12(c). For the four study sites, PNA < 0.5 is found in the small area near the traffic noise sources. PNA is always greater than 0.5 for most of the spaces in the four sites. In contrast, PTA < 0.5 for most of the spaces is always observed in the worst-case scenario as shown in Figs 9(c), 10(c), 11(c), and 12(c). For

high-level retail planning, $\overline{PTA}$ and $\overline{PNA}$ are representative metrics. $\overline{PNA}$ is always far beyond 0.5 in each site, which means no immediate action is required to mitigate traffic noise if the retail places are not near the planned roads in the business district. Considering Singapore's relatively low wind speed (< 3 m/s), solar irradiance becomes the essential parameter affecting $\overline{PTA}$. The results (Figs 9(a), 10(a), 11(a), and Fig 12(a)) show a high correlation between $\overline{PTA}$ and solar irradiance, as shown in Fig 2. As such, $\overline{PTA}$ is observed reaching the minimum at noon of both days, where the global solar irradiance is the maximum each day. To ensure that $\overline{PTA}$ can at least reach the baseline of 0.5 all the time, we evaluate the improving $\overline{PTA}$ at the hot times (Figs 9(d), 10(d), 10(e), 11(d), 12(d) and 12(e)) by increasing wind speed, blocking solar irradiance, or adopting above both simultaneously. In these contour plots, the solid line separates the parametric space to achieve $\overline{PTA} \geq 0.5$ (i.e., the green-colored region pointed by the arrow in contour plots) and $\overline{PTA} < 0.5$ (i.e., the warm-colored region). The intersections of the solid lines and vertical axis and horizontal axes represent the minimum requirements for adopting the only increasing wind speed and only blocking solar irradiance to achieve $\overline{PTA} = 0.5$. The parametric space of the required conditions to achieve $\overline{PTA} = 0.5$ at 13:30 (the lower panel of Figs 9(d), 10(d), 11(d), and 12(d)) of the sunny day is much smaller than that at any other time (the upper panel of Figs 9(d), 10(d), 10(e), 11(d), 12(d), and 12(e)). The corresponding required conditions for

achieving $\overline{PTA}$ ≥ 0.5 at each site at 13:30 (the worst-case scenario) can be applied to achieve $\overline{PTA}$ ≥ 0.5 at any other time. For example, in the lower panel of Fig 10(d), the requirements for achieving $\overline{PTA}$ ≥ 0.5 also satisfy that as shown in the upper panel of Fig 10(d) and Fig 10(e).

If considering using the single strategy for improving $\overline{PTA}$ at the hottest time (i.e., 13:30 of the sunny day as shown in the lower panel of Figs 9(d), 10(d), 11(d), and 12(d), blocking the solar irradiance is an efficient way to achieve $\overline{PTA}$ = 0.5 when the solar radiation is the direct dominant with a small zenith angle. Although the layout of surrounding buildings is different at study sites, the improvement trends of $\overline{PTA}$ are similar, and $\overline{PTA}$ at all four sites can be improved from <0.2 to 0.5 by blocking 54-68% of solar irradiance, respectively. It should be noted that $\overline{PTA}$ =0.5 at the site (iv) (market village) can be achieved by blocking 54%, which is the minimum among the four sites. This fact means that blocking solar irradiance is more efficient to improve $\overline{PTA}$ in open spaces that agree with the findings from [61, 62, 63, 64]. Increasing wind speed is another way to improve $\overline{PTA}$. However, the results at 13:30 of the sunny day (i.e., the lower panel of Figs 9(d), 10(d), 11(d), and 12(d)) show that $\overline{PTA}$ is always smaller than 0.5 even though the wind speed is increased by 10 m/s, which is the typical maximum wind speed for mechanical ventilation [60]. In contrast, the results at any other times when 0.3< $\overline{PTA}$ <0.5 show that $\overline{PTA}$ ≥0.5 is achievable by increasing the

wind speed to 0.2-3.7 m/s. As stated above, we can deduce that blocking solar irradiance can efficiently improve $\overline{PTA}$ at any time on both days, especially when $\overline{PTA}$ <0.2 (at 13:30 of the sunny day). On the other hand, enhancing ventilation by increasing wind speed is effective at the time when $\overline{PTA}$ >0.3 (e.g., the cloudy day) in the first place.

Furthermore, the combined effects of blocking solar irradiance and increasing wind speed can be a more efficient way to improve $\overline{PTA}$ than just adopting shade or boosting the wind. In the contour plots from Figs 9-12, every point on the solid line represents a combination of conditions to reach $\overline{PTA}$ = 0.5. For instance, the lower panel of Figs 9(d), 10(d), 11(d), and 12(d) show that the required blocking percentage of solar irradiance can be decreased ~10% for reaching $\overline{PTA}$ ≥0.5 at 13:30 of the sunny day when the wind speed is increased by 2 m/s which is roughly conventional fan airspeed driven by ceiling fans [65]. In practicality, this way provides flexibility in architecture and has been widely used around al fresco restaurants, outdoor amphitheaters, and some sheltered pedestrian ways in Singapore.

The main implication of the result is that $\overline{PTA}$ and $\overline{PNA}$ give an indication on the percentage of visitors who find the location comfortable enough for them to patronize or hang around al fresco dining and pop-up retail kiosks in the outdoor environment. In this study, $\overline{PTA}$ is a dominant factor to determine people's willingness to retail queue

or gather at all four sites at the hot time. On the other hand, although the $\overline{PNA}$ is always >0.5 at each site, traffic noise issue in the area with $PNA < 0.5$ still should be considered in the retail planning. From the spatial color mapping plots (Figs 9(d), 10(d), 11(d), and 12(d)), the regions with $PNA < 0.5$ are always associated with $PTA < 0.5$, marked in purple. This clearly defines the zone that is not recommended for outdoor retails if the thermal and acoustic comfort cannot be improved simultaneously. According to the results, specific guidelines for retail planning for each site can be made as follows. At site (i) (campus boulevard) where is a thoroughfare with buildings on either side, $\overline{PTA}$ can be expected to improve by installing pop-up shopping areas next to the side buildings and awnings for visitor queuing. To increase local airspeed using outdoor ventilation devices will further improve $\overline{PTA}$. In comparison between the results, Figs 9(a) and 10(a), site (ii) (heritage trail) is hotter than site (i) due to lacking buildings on the side to block the solar irradiance. The building shadows can directly improve the thermal comfort [66, 67]. The site (ii) is planned to be a pedestrian-only pathway renovated from an old road. Thus, $\overline{PTA}$ here can be expected to improve by installing a sheltered walkway [68] and planting street trees with overhead coverage [54, 69, 70]. The location of retail shops or kiosk that may attract long queues are not recommended in the purple zones (Figs 9(c) and 10(c)) near the road junctions at the site (i) and (ii). The site (iii) (Nexus) has a higher $\overline{PTA}$ benefiting from the

spontaneous shading by three-sides surrounding buildings. With sufficient shading percentage by the shelter and increased wind speed via ceiling fans suggested by Fig 11(d), the tenants or visitors are expected to be more willing to stay in the outdoor area for al fresco dining [71] and pop-up shopping over a long time. In addition, site (iii) is the only one for outdoor activities free from interfering traffic noise in this study as seen in Fig 11(c). The site (iv) (market village) is an open area for outdoor activities. According to the results from Fig 12(d), $\overline{PTA}$ can be efficiently improved by blocking solar irradiance via installing the canopy frames and planting trees. Keeping away from the area affected by traffic noise, the purple zone in Fig 12(c), outdoor amphitheaters with ceiling fans have a suitable setting for outdoor events.

If the shading techniques and mechanical wind devices may be challenging to deploy practically to reach the condition for $\overline{PTA}$ improvement, people may tend to remain indoors by relying on air conditioning during the hot time. On the other hand, outdoor activities become inappropriate in the area exposed the traffic noise, i.e., purple zones in Figs 9(c), 10(c), and 12(c). Because of that, urban planners and architects can choose retail options at outdoor locations for specific purposes. We have pointed out that the threshold of $\overline{PTA}$ = 0.5 and $\overline{PNA}$ = 0.5 is the baseline used in this study but not universal. The policy of the $\overline{PTA}$ and $\overline{PNA}$ standards can be formulated depending on the actual circumstances, e.g., $\overline{PTA}$ ≥0.6. According to Table 4 (the results from

Figs 9(d), 10(d), 10(e), 11(d), 12(d), and 12(e)), the architects and urban planners can find the corresponding conditions for improving $\overline{PTA}$ locally.

We note that this study had only examined the potential improvement on $\overline{PTA}$ via the simplified parametric analysis (i.e., decreasing the solar irradiance and increasing the wind speed) under ideal conditions, without considering trees, shades, or outdoor fans in the simulations. Under the ideal conditions, we assumed that the solar radiation level and wind speed have implicitly incorporated the effects of trees, shades, or outdoor fans. The double parametric space in terms of solar radiation and wind speed provides a guideline for architects to quickly identify the optimal microclimate conditions for planning the infrastructure and landscape combination during the scenario design stage. The exact details of the surrounding environment, i.e., shades, trees and fans, can be inversely designed based on the optimal microclimate condition, which is out of the scope of this paper and requires further study. Although the evaluation provides the parametric scope and the options to improve outdoor thermal comfort for future plan of combining landscape and infrastructure (e.g., trees with shelter walkaways, green walls with outdoor ventilation fans, and etc.) in design, the solar-wind-coupled simulations and on-site measurements with regards the as-built remains to be done at a later stage.

4. Conclusions

In conclusion, this paper evaluates the outdoor environmental comfort of an emerging

district in Singapore. This evaluation was based on Integrated Environmental Modeler's (IEM) simulation results, a 3D coupled physics simulation platform that includes solar radiation, wind, thermal, and traffic noise, using a single unified set of geometrical input. For the architectural purpose, we selected two representative days in the hottest period of Singapore (i.e., from March to June), which are the spring equinox (sunny day) and the summer solstice (cloudy day), respectively. The sunny day can be regarded as the worst-case scenario and the cloudy day is the reference scenario to conduct simulations considering the material properties of infrastructure and terrain features. TMY data and the BCA Green Mark standards were adopted in solar and wind simulations as the weather conditions in solar and wind simulations. The traffic noise sources were categorized based road characteristics (such as the number of lanes and average speed of the vehicles during the peak hour) and applied in the noise simulations to represent the worst-case traffic noise scenario. From the results of the IEM, we further derived environmental indices (i.e., TSV and L) on the four studied sites with high pedestrian traffic. These four sites are either pedestrian thoroughfares, or gathering areas for potential outdoor community events and activities for the tenants and residents staying near the business district. The people-centric acceptability factors (i.e., PTA and PNA), defined as a function of TSV and L, were introduced to define the degrees of environmental comfort. The area-averaged factors $\overline{PTA}$ and $\overline{PNA}$ with the

corresponding variance were applied to evaluate each studied site. The variance of PTA and PNA come from the spatial distribution of environmental conditions in this business district. In the worst-case scenario, i.e., at 13:30 on the sunny day, $PTA < 0.5$ is observed almost everywhere along all sites. In the meantime, the places, $PNA < 0.5$, are found concentrated near the noise sources, which overlaps with a part of the $PTA < 0.5$ region. These areas (purple zones in Figs 9(c), 10(c), and 12(c)) are not recommended for outdoor activities for retail purposes because improving PNA by constructing the noise barriers may further degrade the local PTA. For the high-level retail planning, we set the acceptable criterion of $\overline{PTA}$ = 0.5 and $\overline{PNA}$ = 0.5, meaning most people would be satisfied with the thermal and acoustic environment, respectively. $\overline{PNA}$ is always far beyond 0.5 in the studied sites, which means that most people here are satisfied with the acoustic comfort influenced by the proximity and road categories in the area except for people stay in purple zones as shown in Figs 9(c), 10(c), and 12(c). In contrast, $\overline{PTA}$ is highly dependent on global solar irradiance due to the low-wind-speed conditions which is always observed < 3 m/s in this study. Thus, $\overline{PTA}$ trends towards the minimum at noon of the sunny day when global solar irradiance is maximum. We proposed a simplified parametric analysis to improve the $\overline{PTA}$ when $\overline{PTA}$ < 0.5, (1) by blocking the solar irradiance and (2) by increasing the wind speed. The improvement of $\overline{PTA}$ is a function of the shading percentage and the mechanical

wind speed. Considering only a single strategy is adopted, blocking solar irradiance is efficient to improve $\overline{PTA}$ at 13:30 of the sunny day, i.e., the hottest time. $\overline{PTA}$ rises from <0.2 to 0.5 can be achieved by blocking 54%-68% solar irradiance among the four sites. However, when $\overline{PTA}$ <0.3, the improvement over baseline is difficult to achieve by enhancing the local wind speed by up to 10m/s, which is the maximum boost provided by typical ventilation fans. Enhancing the mechanical wind speed is effective to improve $\overline{PTA}$ at the time when $\overline{PTA}$ >0.3 (e.g., the cloudy day). Coupling the shading and boosting wind can be a more efficient way to improve $\overline{PTA}$. For example, increasing a 2 m/s wind speed requires a smaller shelter that blocks ~10% less solar irradiance as compared to a shading only solution to maintain $\overline{PTA}$ ≥0.5. This combination provides more flexibility for architecture based on site constraints. Moreover, the quantitative analysis of $\overline{PTA}$ and the well-defined uncomfortable region, purple zones as depicted in Figs 9(c), 10(c), and 12(c), can help develop strategies to improve environmental comfort to encourage outdoor gatherings and establish the reference of outdoor retail options for each site. Considering the surrounding building layouts and the $\overline{PTA}$ improvement at each study site, potential outdoor retail options were suggested. For instance, placement of pop-up kiosks with outdoor queues and al fresco dining options are preferred for areas where the thermal and acoustic acceptability are higher than the benchmark (i.e., 50%) most of the time.

Based on the assessment, the $\overline{PTA}$ can be set as the target for specific purposes, instead of the baseline of $\overline{PTA}$ = 0.5. If the shading techniques and mechanical wind devices are challenging to deploy to reach the target of $\overline{PTA}$ practically or the area exposed to the traffic noise issue at the same time, then air-conditioned stores and restaurants can be set up. To summarize the work, the assessment based on coupled-physics simulations can guide architects and urban planners to take the appropriate retail option for practical purposes of the district. Furthermore, according to the assessment of environmental acceptability, the people-centric and context-specific plans (such as outdoor comfort and retail planning) can be evaluated, and suitable infrastructure or mitigation measures can be put in place, to enable the planned activities even if the location might not be sufficiently comfortable (i.e., low $\overline{PTA}$) or for the planned activity in the first place. Possible topics for further exploration include solar-wind-noise coupled simulations, calibrated against on-site measurements, of the scenarios with future landscape and infrastructure combinations. (e.g., trees with shelter walkaways, green walls with outdoor ventilation fans etc.) In addition, this work could be extended to include key performance indicators (KPIs), such as building energy consumption and renewable energy options.

Author Contributions

Conceptualization: Po-Yen Lai, Wee Shing Koh.

Data curation: Po-Yen Lai, Dias Leong, Hyosoo Lee.

Formal analysis: Po-Yen Lai.

Funding acquisition: Wee Shing Koh.

Investigation: Po-Yen Lai, Wee Shing Koh, Harish Gopalan.

Methodology: Po-Yen Lai, Harish Gopalan, Huizhe Liu, Wee Shing Koh, Dias Leong, Hyosoo Lee, Gibert Peh, Gilbert Cher, Cheng Hui Eng, Jia Li Goh.

Project administration: Wee Shing Koh, Johnathan Goh.

Resources: Edmund Tan, James Tan.

Software: Harish Gopalan, Huizhe Liu, Dias Leong, Hyosoo Lee, Jacob Ang.

Supervision: Wee Shing Koh, Edmund Tan, James Tan.

Validation: Po-Yen Lai.

Visualization: Po-Yen Lai, Dias Leong, Jiun Yeu Lim, Hyosoo Lee.

Writing – original draft: Po-Yen Lai.

Writing – review & editing: Wee Shing Koh, Harish Gopalan, Huizhe Liu, Dias Leong, Hyosoo Lee, Johnathan Goh, Jiun Yeu Lim, Jacob Ang, Gibert Peh, Gilbert Cher, Cheng Hui Eng, Jia Li Goh, Edmund Tan, James Tan.

References

1. Meteorological Service Singapore, Past Climate Trends; 2021 [cited 2021 Jun 17] Available from: http://www.weather.gov.sg/climate-past-climate-trends/.

2. Watkins R, Palmer J, Kolokotroni M. Increased Temperature and Intensification of the Urban Heat Island: Implications for Human Comfort and Urban Design. Built Environment. 2007;33(1):85-96.
3. Wong NH, Yu C. Study of green areas and urban heat island in a tropical city. Habitat International. 2005;29:547-558.
4. Hung T, Uchihama D, Ochi S, Yasuoka Y. Assessment with satellite data of the urban heat island effects in Asian mega cities. International Journal of Applied Earth Observation and Geoinformation, 2008,8:31-48,.
5. SMILE - Guidelines for Road Traffic Noise Abatement; 2003 [cited 2022 Jan 18] Available from: https://webgate.ec.europa.eu/life/publicWebsite/project/details/1780#:~:text=%22S%20M%20I%20L%20E%20%2D%20Guidelines%20for%20Road%20Traffic%20Noise%20Abatement%22%20(2%20MB).
6. Kousis I, Pisello AL. For the mitigation of urban heat island and urban noise island: two simultaneous sides of urban discomfort. Environmental Research Letters. 2020,103004,15.
7. Chin C, Thang ZY, Saju S. Study on impact of noise annoyance from highway traffic in Singapore City, in 178th Meeting of the Acoustical Society of America: Architectural Acoustics: Paper 1aAA6, San Diego, California, 2019.
8. Tumini I, Higueras García E, Baereswyl Rada S. Urban microclimate and thermal comfort modelling: strategies for urban renovation. International Journal of Sustainable Building Technology and Urban Development. 2016,7(1),22-37.
9. Yu X, Zhang Q, Kang J, Cui F. Predicting integrated thermal and acoustic performance in naturally ventilated high-rise buildings using CFD and FEM simulation. Building Simulation. 2018,11(3),507-518,.
10. Kanda M. Progress in urban meteorology: a review. J Meteorol Soc Jpn. 2007,85B,363-383.
11. Toparlar Y, Blocken B, Maiheu B, Heijstd GJFV. A review on the CFD analysis of urban microclimate. Renewable and Sustainable Energy Reviews. 2017,80,1613-1640.
12. Wang B, Koh WS, Liu H, Yik J, Bui VP. Simulation and validation of solar heat gain in real urban environments. Building and Environment. 2017,123,261-276.

13. Allegrini J, Dorer V, Carmeliet J. Coupled CFD, radiation and building energy model for studying heat fluxes in an urban environment with generic building configurations. Sustainable Cities and Society. 2015,19,385-394.
14. Qu Y, Milliez M, Musson-Genon L, Carissimo B. Numerical study of the thermal effects of buildings on low-speed airflow taking into account 3D atmospheric radiation in urban canopy. Journal of Wind Engineering and Industrial Aerodynamics, 2012,104,474-483.
15. Roth M, Lim VH., Evaluation of canopy-layer air and mean radiant temperature simulations by a microclimate model over a tropical residential neighbourhood. Building and Environment. 2017,112,177-189.
16. Israelsson S, Sound Propagation in the Atmosphere near the Ground, Swedish: University of Uppsala, 1979.
17. Hornikx M, Dohmen M, Conen K, van Hooff T, Blocken B. The wind effect on sound propagation over urban areas: Predictions for generic urban sections. Building and Environment. 2018,144,519-531,.
18. Wagner S, Bareiß R, Guidati G. Wind Turbine Noise, Berlin, Heidelberg: Springer, 1996.
19. Matzarakis A, Rutz F, Mayer H. Modelling radiation fluxes in simple and complex environments: basics of the RayMan model. Int J Biometeorol. 2010,54(2),131-139.
20. Lindberg F, Holmer B, Thorsson S. 1.0-Modeling spatial variations of 3-D radiant fluxes and mean radiant temperature in complex urban settings. Int J Biometeorol. 2008,52(7),697-713.
21. Bruse M and Fleer H Simulating surface-plant-air interactions in urban environments with a three dimensional numerical model. Environ. Modell. Softw. 1998, 13, 374-384.
22. Zhao WJ, Liu EX, Poh HJ, Wang B, Gao SP and Png CE. 3D traffic noise mapping using unstructured surface mesh representation of buildings and roads. Applied Acoustics. 2017,127,297-304.
23. Wise D, Boppana V, Li K, Poh H, Effects of Minor Changes in the Wind Direction on Urban Flow Simulations. Sustainable Cities and Society. 2018,37,492-500.
24. Gopalan H. Evaluation of Wray-Agarwal turbulence model for simulation of neutral and non-neutral atmospheric boundary layers. Journal of Wind Engineering and Industrial Aerodynamics. 2018,182, 322-329.

25. Höppe P. Different aspects of assessing indoor and outdoor thermal comfort. Energy and Buildings. 2002,34(6),661-665.
26. Chen L, Ng E. Outdoor thermal comfort and outdoor activities: a review of research in the past decade. Cities. 2012,29(2),118-125.
27. Höppe P, The physiological equivalent temperature – a universal index for the biometeorological assessment of the thermal environment, International Journal of Biometeorology. 1999,43,71-75.
28. Gagge AP, Stolwijk JAJ, Hardy JD. Comfort and thermal sensations and associated physiological responses at various. Environmental Research. 1967,1,1-20.
29. Błażejczyk K, Jendritzky G, Bröde P, Fiala D, Havenith G, Epstein Y, et al. An introduction to the Universal Thermal Climate Index (UTCI). Geographia Polonica. 2013,86(1),5-10.
30. Fanger PO. Thermal Comfort. Copenhagen: Danish Technical Press. 1970.
31. ASHRAE, ASHRAE Standard 55-2004. Thermal environmental conditions for human occupancy, American Society of Heating, Refrigerating and Air-Conditioning Engineering, Atlanta, Georgia, USA. 2004.
32. Zaki N, Rijal S, Ahmad H, Razak N and Razak A. Field study of pedestrians' comfort temperatures under outdoor and semi-outdoor conditions in Malaysian university campuses Othman. International Journal of Biometeorology. vol. 2021,65,453-477,.
33. Yang W, Wong NH, Jusuf SK. Thermal comfort in outdoor urban spaces in Singapore. Building and Environment. 2013,59,426-435,.
34. Berglund B, Lindvall T, Schwela DH. Guidelines for Community Noise. London, United Kingdom: World Health Organization. 1999.
35. Mui K, Wong L, Wong Y. Acceptable noise levels for construction site offices. Building Serv. Eng. Res. Technol. 2009,30(1),87-94,.
36. Garg N, Maji S, A critical review of principal traffic noise models: Strategies and implications. Environmental Impact Assessment Review. 2014,46,68-81,.
37. Department of Transport, Welsh Office. Calculation of Road Traffic. United Kingdom: HSMO Publication. 1988.
38. Authority LT. Road Line Plan Explanatory Notes; [cited 2022 Jan 19] Available from: https://www.lta.gov.sg/content/dam/ltagov/industry_innovations/industry_matters/development_construction_resources/road_line_plan/pdf/Road_line_Plan_Explanatory_notes_final.pdf.

39. Climate of Singapore, Meteorological Service Singapore; 2021 [cited 2021 Aug 13] Available from: http://www.weather.gov.sg/climate-climate-of-singapore/.

40. ASHRAE. International Weather for Energy Calculations (IWEC Weather Files) Users Manual and CD-ROM, Atlanta: ASHRAE. 2001.

41. Green Mark for Non-Residential Buildings NRB: 2015, [cited 2021 Jan 6] Available from: https://www.bca.gov.sg/greenmark/others/Green_Mark_NRB_2015_Criteria_Draft_R3.pdf.

42. Perez R, Seals R, Michalsky J. All-weather model for sky luminance distribution-Preliminary configuration and validation. Solar Energy. 1993,50(3),235-245,.

43. Robinson D, Stone A. A simplified radiosity algorithm for general urban radiation exchange. Build. Serv. Eng. Res. Technol. 2005,26(4),271-284.

44. Lam KP, Mahdavi A, Ullah M, Ng E, Pal V. Comparative study of sky luminance models in the tropical context. Building Simulation. 1997,1,339.

45. Yang D. Solar radiation on inclined surfaces: Corrections and benchmarks. Solar Energy. 2016,136(15),288-302,.

46. Han X, Wray T, Agarwal R. Application of a New DES Model Based on Wray-Agarwal Turbulence Model for Simulation of Wall-Bounded Flows with Separation, in AIAA 2017-3966, Session: Applications of Fluid Mechanics, 47th AIAA Fluid Dynamics Conference, Denver, Colorado. 2017.

47. Rumsey C, Smith B, Huang G. Description of a website resource for turbulence modeling verification and validation. in Session: FD-32: Turbulent Flows: RANS and Hybrid Methods, 40th Fluid Dynamics Conference and Exhibit, Chicago, Illinois. 2010.

48. Monin A, Obukhov A. Basic laws of turbulent mixing in the surface layer of the atmosphere. Contrib. Geophys. Inst. Acad. Sci. USSR. 1954,151(163),187.

49. Foken T. 50 years of the Monin–Obukhov similarity theory. Boundary-Layer Meteorology. 2006,119(3),431-447.

50. Raithby GD, Chui EH. A Finite-Volume Method for Predicting a Radiant Heat Transfer in Enclosures With Participating Media. Journal of Heat Transfer. 1990,112(2),415-423.

51. Calculation of road traffic noise. Her Majesty's Stationery Office, London. 1988.

52. Zhao WJ, Boppana VBL, Liu EX, Poh HJ, Li KW, Tan TT. Traffic with Noise Modeling Consideration of Meteorological Effect. in INTER-NOISE and NOISE-CON Congress and Conference Proceedings, Hamburg GERMANY. 2016.
53. ISO 7726:1998 Ergonomics of the thermal environment -- Instruments for measuring physical quantities, Organization, International Standard. 1998.
54. Lai PY, Koh JH, Koh WS, Liu H. Effectively modeling surface temperature and evaluating mean radiant temperature in tropical outdoor industrial environments. Building and Environment. 2020,169,106277.
55. Hock KK. Environmental Protection Division Report. National Environmental Agency, Singapore; 2012, [cited 2021 May 31] Available from: https://www.nea.gov.sg/docs/default-source/resource/publications/environmental-protection-division-annual-report/epd-2011.pdf.
56. Wieringa J. Updating the Davenport roughness classification. Journal of Wind Engineering and Industrial Aerodynamics. 1992,41(1),357-368.
57. Nouri AS, Costa JP, Santamouris M, Matzarakis A. Approaches to Outdoor Thermal Comfort Thresholds through Public Space Design: A Review. Atmosphere, 2018,9(3),108.
58. Yang W, Wong NH, Lin Y. Thermal Comfort in High-rise Urban Environments in Singapore. Procedia Engineering. 2015,121, 2125-2131.
59. Malik A, Bongers C, McBain B, Rey-Lescure O, Dear Rd, et al. The potential for indoor fans to change air conditioning use while maintaining human thermal comfort during hot weather: an analysis of energy demand and associated greenhouse gas emissions. Lancet Planet Health. 2022,6,E301-E309.
60. Silva W, Moura D, Carvalho-Curi T, Seber R, Massari J. Evatuation system of exhaust fans used on ventilation system in commercial broiler house. Journal of the Brazilian Association of Agricultural Engineering. 2017,5(37),887-899.
61. Shashua-Bar L, Ioannis X, Milo H. Passive cooling design options to ameliorate thermal comfort in urban streets of a Mediterranean climate (Athens) under hot summer conditions. Build. Environ. 2012,57,110-119.
62. Cheung P, Jim C. Comparing the cooling effects of a tree and a concrete shelter using PET and UTCI. Build. Environ. 2018,130,49-61.

63. Nasrollahi N, Ghosouri A, Khodakarami J, Taleghani M. Heat-Mitigation Strategies to Improve Pedestrian Thermal Comfort in Urban Environments: A Review. Sustainability, 2020,12,10000.
64. Zaki SA, Syahidah S, Shahidan MF, Ahmad MI, Yakub F, et al. Assessment of Outdoor Air Temperature with Different Shaded Area within an Urban University Campus in Hot-Humid Climate Sustainability. 2020,12,5741.
65. Rafterya P, Fizer J, Chen W, He Y, Zhang H, Arens E, et al. Ceiling fans: Predicting indoor air speeds based on full scale laboratory measurements. Building and Environment. 2019,115,210-223.
66. Zaki SA, Othman N, Syahidah S, Yakub F, Muhammad-Sukki F, et al. Effects of urban morphology on microclimate parameters in an urban university campus. Sustainability. 2020,12(7),2962.
67. Lau KKL, Ren C, Ng JHE, Numerical modelling of mean radiant temperature in high-density sub-tropical urban environment. Energy and Buildings, 2016,114,80-86.
68. Subramanian R, Tunçer B, Binder A. Thermal Comfort Based Performance Appraisal of Covered Walkways in Singapore. in 24th CAADRIA Conference, New Zealand. 2019.
69. Liu H, Lim JY, Thet BWH, Lai PY, Koh WS. Evaluating the impact of tree morphologies and planting densities on outdoor thermal comfort in tropical residential precincts in Singapore. Building and Environment. 2022,221,109268.
70. Zaki SA, Toh HJ, Yakub F, Saudi ASM. Effects of roadside trees and road orientation on thermal environment in a tropical City. Sustainability. 2020,12,1053.
71. Vít V, Kopp J. Typology of Outdoor Seating Areas of Restaurants Based on Factors Influencing Their Thermal Comfort, A Case Study of Pilsen City Centre, Czechia. Journal of Settlements and Spatial Planning. 2019,10(2),131-142.

Outdoor environmental comfort evaluation for retail planning in a tropical business district using Integrated Environmental Modeller

Po-Yen Lai[1]*, Wee Shing Koh[1, 2], Harish Gopalan[1], Huizhe Liu[1], Dias Leong[3], Hyosoo Lee[3], Johnathan Goh[3], Jiun Yeu Lim[1], Jacob Ang[3], Gibert Peh[3], Gilbert Cher[3], Cheng Hui Eng[3], Jia Li Goh[3], Edmund Tan[3], James Tan[3]

[1]A*STAR Institute of High Performance Computing, 1 Fusionopolis Way, #16-16 Connexis, 138632, Singapore

[2]Department of Physics, National University of Singapore, 2 Science Drive 3, 117551, Singapore

[3]Smart District / New Estates 2 / Urban Design & Architecture Divisions, JTC Corporation, 8 Jurong Town Hall Road, 609434, Singapore

Abstract

This research proposes a simulation-based assessment of outdoor thermal and acoustic comfort for a planned business urban district in Singapore for retail planning using a customized OpenFOAM-centric multi-physics environmental simulation platform called the Integrated Environmental Modeller (IEM). IEM was employed to simulate the coupled impacts of solar radiation on wind and air temperature and wind and air

temperature effects on traffic noise propagation in the district on the equinox and solstice day of the hottest period. Using IEM simulation results, we computed the thermal and acoustic comfort acceptability indicators derived from local field studies' results. –The spatial distribution of environmental comfort acceptability indicators in the worst-case scenario can be used to distinguish the zones exposed to thermal or noise influence. The spatial distribution of environmental comfort acceptability indicators in the worst-case scenario can be used to distinguish the zones exposed to thermal or noise influence. The noise-affected zones are near the main roads and overlap a part of the thermal-affected area. The thermal-affected area is almost everywhere in the studied sites in the worst-case scenario. ConductingHaving outdoor retail activities spaces in thewith both poor thermal- and noiseacoustic overlapped zonescomfort is not recommended if the thermal and acoustic comfort cannot be improved simultaneously. For the high-level retail planning, *Compared with acoustic comfort, acceptable thermal comfort was more challenging to achieve.* wWe provided a simplified parametric analysis considering solar irradiance blockage and wind speed enhancements, is provided. Considering the worst-case scenario, ≥50% thermal acceptability can be achieved by blocking 54%-68% solar irradiance among the pedestrian thoroughfares and the retail spaces. Coupled together, blocking the solar irradiance and enhancing the wind speed can further improve thermal comfort locally. These results can guide the

Formatted: Font: Italic

retail mix (e.g., al fresco restaurants, pop-up kiosks etc.) near high footfall areas and provide reference for future plans combining landscape and infrastructure, (e.g., trees with shelter walkaways, green walls with outdoor ventilation fans etc.) taking into account the environmental acceptability of people working in or visiting the tropical urban district~~district~~.

Keywords: Retail planning, Integrated Environmental Modeller (IEM), ~~Tropical urban district,~~ Thermal comfort~~ index~~, Acoustic comfort~~ index, Environmental evaluation~~, ~~Retail planning~~Tropical urban district.

*Electronic address: lai_po-yen@ihpc.a-star.edu.sg

1. Introduction

Over the past two decades, rapid urbanization in Singapore has affected human livability in outdoor thermal and acoustic environments. [1] Urban corridors and high-rise urban structures absorb the intense solar radiation from the sun in the day and radiate heat at night. This induces a higher urban air temperature than that in rural areas, producing thermal stress [2], i.e., urban heat island (UHI) [3, 4]. As modern architectures and population exponentially grows, urban transportation needs have also vastly increased. With high road density and heavy traffic, traffic noise accounts for up to 80% of whole noise sources in urban areas [5]. Thus, the urban area is significantly

noisier than the rural area, i.e., urban noise island (UNI) [6, 7]. UHI and UNI coexist and seriously affect urban areas' societal life and health conditions [6]. Therefore, assessing thermal and acoustic environments for improving the urban livability of planned districts in Singapore is essential in urban planning and architectural design.

Microclimate variables (solar radiation, air temperature, and wind speed) and noise propagation together characterize the thermal and acoustic environments in urban areas [8]. To conduct thermal and acoustic environmental studies and assessments, numerical simulation has been widely used [9]. Such simulations can provide detailed information of the physical variables of interest at any point within the domain at different scales. [10, 11] Considering the typical weather condition in Singapore: a sunny day in the tropics with low average wind speeds, buoyancy-driven turbulence induced by solar radiation is the most critical parameter that influences urban microclimate simulations [12, 13, 14, 15] in which the core physical model is the wind-thermal interaction between wind dynamics and heat transfer on building and ground surfaces in the urban environment. Meanwhile, wind and thermal shear also impact the traffic noise levels in dense canopies [16, 17]. Therefore, a coupled wind-thermal and noise propagation model is valuable to simulate and evaluate the thermal and acoustic environment in dense urban spaces for lower wind speed conditions [18]. While, by using existing simulators (e.g., Rayman [19], SOlar Long Wave Environmental Irradiance Geometry

model (SOLWEIG) [20], and ENVI-met [21]~~[17]~~), many environmental studies have been done on simulations of solar radiation, wind dynamics, and noise propagation separately or solar-wind at most, there were no reported studies on the coupled solar-wind-noise simulations to our best knowledge. Hence, a novel coupled solar-wind-noise modeling tool, i.e., Integrated Environmental Modeller (IEM), has been developed to conduct coupled solar-wind-noise simulations in Singapore at different spatial scales from individual buildings to districts and regions ~~[12, 21, 22, 23]~~. The IEM framework integrates environmental models, including solar radiation [12], wind dynamics [23, 24], and noise propagation [22], to captures the correlation of several physical schemes in simulations. The outputs of such microscale simulations enable further evaluation of thermal and acoustic comfort in the Singapore's urban environment.

To adequately evaluate thermal comfort in outdoor spaces, the human physiological thermoregulation mechanisms, subjective psychological perceptions, cultural backgrounds, and weather conditions should be taken into account [25, 26]. Several thermal comfort indices have been proposed in different scopes of application, including Physiological Equivalent Temperature (PET) [27], Standard Effective Temperature (SET) [28], Universal Thermal Climate Index (UTCI) [29], Predicted Mean Vote (PMV) [30], Thermal Sensation Vote (TSV) [31], to name a few. To realize

a fair thermal comfort assessment in a tropical climate, it is better to consider the results from local field studies in the existing thermal comfort index, e.g., [32, 33]. Based on the statistical results from the field studies conducted in the Singapore's outdoor environment [33], such work proposed a correlation between TSV and operative temperature derived as a linear regression formula. TSV depends on the local microclimate factors and human physiological thermoregulation on the Singaporean background. The corresponding thermal acceptability was further derived as a function of TSV using binary logistic regression to quantify the occupants' reaction to the thermal environment.

Researchers commonly recognized the noise level as the acoustic comfort index, e.g., World Health Organization (WHO) noise guideline [34]. Similar to thermal acceptability, the research team proposed a relationship between the noise acceptability and noise level to quantify the occupants' reaction to the environmental noise according to the field study conducted in construction site offices in Hong Kong [35]. In a modern urban area, the main component of environmental noise comes from road traffic [5]. Traffic noise modeling is essential to evaluate the acoustic comfort in urban spaces by calculating the noise mapping, which helps qualitatively identify the noise level at a receiver position due to traffic emission source. [36] Traffic noise modeling considers traffic volume (e.g., average vehicle speed), composition (e.g., proportion of large

vehicles), road geometry, and absorption features of ground cover in the calculation. The Calculation of Road Traffic Noise (CRTN) [37] is the first systematic scheme developed to predict the noise level due to road traffic. The CRTN method has been applied to predict the traffic noise and validated experimentally in Singapore [22].

This research is the first to propose a simulation-based workflow for simultaneously assessing the thermal and acoustic environmental comforts of a planned urban district in Singapore. We conducted solar-wind-noise simulations based on a three dimensional (3D) urban district modeled by architects on equinox and solstice day during the hottest period of Singapore weather. Material properties, terrains, building appearance with 3D geometric details, and the worst-case peak period road traffic noise sources were considered in simulations. Using the local survey-based sensation models [33, 35], we further evaluate the acceptability of thermal and acoustic comfort of pedestrian thoroughfares and the retail spaces based on outputs from Integrated Environmental Modeller (IEM). [12, 21, 22, 23] Moreover, we perform the parametric study of the decreasing solar irradiance and the increasing wind speed to show room for improvement of outdoor environmental thermal comfort theoretically. The evaluation provides the guidance of high pedestrian footfall areas for retail and event planning (i.e., al fresco dining, popular pop-up retail kiosks with long queues, outdoor theme business community events, etc.) that considers thermal and acoustic comfort in the new

business district. This workflow provides a reference framework for incorporating environmental comfort modeling into future landscape-infrastructure planning as part of early stage architectural design. In this paper, the methodologies, including numerical approaches and the corresponding theoretical basis, are described in Sec. 2. The numerical results, the analysis, and the discussion are presented in Sec. 3. Finally, the concluding remarks on the work are given in Sec. 4.

2. Methodology

2.1 Main features of Study areas and environmental conditions

2.1.1 Main features of the business district

~~**Fig 1(a)** is the aerial view (Google Maps) of t~~The study area, as shown in Fig 1(a)~~,~~ is a coastal district in the north-eastern region of Singapore. The terrain is relatively flat, with an elevation of 15.6 m above sea level. A waterway crosses through the south part of the district. The district's planned business park is marked in white, and the various locations referred to by its respective mukim lot numbers. Fig 1(~~b~~a) shows the computer-aided design (CAD) view of the urban district where the business park is in the region marked within the black box. Fig 1(~~c~~b) represents the zoom-in feature of the marked region. For outdoor comfort evaluations, four study sites with high pedestrian or human traffic are marked in Fig 1(~~c~~b), which are respectively (i) an east-west

Formatted: Font: Not Bold

Formatted: Font: Not Bold

Formatted: Font: Not Bold

Formatted: Font: Not Bold

Formatted: Font: Not Bold

boulevard that connects the business park buildings to the waterfront ("campus boulevard"), (ii) a north-south trail that was converted from an old road to a pedestrian-only walkway ("heritage trail"), (iii) an open podium under a mid-rise office building ("Nexus"), and (iv) an outdoor waterfront plaza to hold gatherings and events for tenants and their customers or families ("market village"). The sites proposed in this study are either the main pedestrian thoroughfares, or gathering areas for outdoor community building and events. Taking advantage of the potential high pedestrian footfall, outdoor retail options are planned along or within these sites. For example, al fresco restaurants and pop-up retail kiosks may be set up around these sites, where visitors spend their time on various shopping, leisure, and dining activities. Thermal and acoustic comfort assessments in this work can be useful for retail planning to ensure that the outdoor areas are comfortable and attractive for outdoor dining, waiting for takeaways, queuing and shopping.

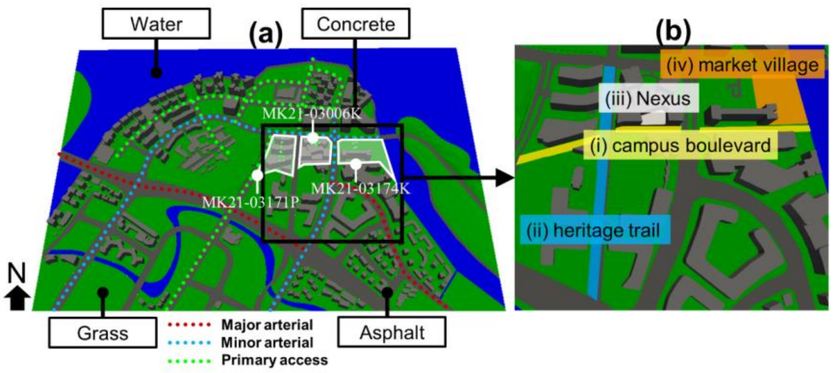

Fig 1. (a) The schematic illustration of ~~Aerial view (from Google Map) of~~ the studied

urban district in the north-east region of Singapore, including the 4four primary materials assignmented of infrastructuresto the 3D geometry and the categorization of3 different road categories traffic noise sources from main roads. White portions are the planned business park with the specific mukim lot numbers MK21-03171P, MK21-03006K and MK21-03174K. **(b)** The schematic illustration of the urban district, including the material assignment of infrastructures and the categorization of traffic noise sources from main roads. **(cb)** The zoom-in feature of the study sites with high pedestrian traffic for the evaluation of comfort factors: (i) campus boulevard, (ii) heritage trail, (iii) Nexus, and (iv) market village.

2.1.2 Environmental conditions for simulations

In Fig 1(ba), the district's urban surfaces are grouped by materials, i.e., concrete, asphalt, grass, and water, which represents the appropriate boundary conditions for IEM coupled simulation. In the various simulations, buildings, roads, greenery, and water body were assigned concrete, asphalt, grass, water in which the surface of each object has been defined own optical, thermal, acoustic properties. Table 1 gives the material properties used in this study.

The district's main roads classification is schematically represented by colored dashed lines in Fig 1(ba). In Singapore, main roads are classified into five categories: expressway, major arterial, minor arterial, primary access, and local access from high to low traffic flow. This classification is purposed for the developer to establish the buffer requirements of surrounding buildings from the road [38] and used to define the traffic noise source in simulations. Table 2 shows that the categories used in this study are defined according to the numerical setup [22] in which the noise source is based on

Formatted: Font: Not Bold

Formatted: Font: Not Bold

Formatted: Font: Not Bold

Formatted: Font: Not Bold

the hourly-averaged equivalent noise level during peak hours derived from measurements. Major arterials (i.e., 71 dB), shown in red, predominantly carry through traffic from one region to another, forming primary avenues of communication for urban traffic movements. Minor arterials (i.e., 70.2 dB), shown in blue, distribute traffic within the major residential and industrial areas. Primary accesses (i.e., 68.3 dB), shown in green, constitute the link between local accesses and arterial roads. In this study, the geometry description of the noise source was modeled as a finite line source at 1 meter above the center of the road surface.

Table 1 Material properties used in this paper.

Material	albedo	emissivity	heat absorptivity	sound absorptivity
asphalt	0.16	0.93	0.93	0.1
concrete	0.21	0.85	0.79	0.1
grass	0.39	0.98	0.61	0.1
water	0.14	0.96	0.86	0.1

Table 2 Road categories used in this paper.

Road Category	Hourly-averaged equivalent noise level (dB)
Major Arterial	71
Minor Arterial	70.2
Primary Access	68.3

The climate in Singapore is hot and humid, with a low average wind speed and abundant solar irradiation throughout the year. [39] The typical meteorological year (TMY) data

[40] for Singapore was adopted as the data source in this study. Two representative days in the hottest period (i.e., from March to June [37] in which the air temperature is commonly greater than the mean daily maximum temperature of 32 ^{o}C based on the climatological reference period between 1991 to 2020 [39]) in Singapore were selected for this study, which are equinox and solstice days respectively. The equinox day was a sunny day on 20 March, where the direct normal irradiance (DNI) mostly dominates over the diffused horizontal irradiance (DHI). Fig 2(a) shows the selected hourly-averaged climate data of sky longwave irradiance, direct normal irradiance, and diffuse horizontal irradiance on the equinox day, respectively. The solstice day was a cloudy day on 21 June. Fig 2(b) shows the corresponding metrological data in which the radiation was diffusely dominated.

Considering that winds in Singapore are generally light, the daily average wind speed and direction were adopted in this study for simplicity. To make sure that the assessments in this study are certificated by the local policy, we adopted the Building and Construction Authority's (BCA) Green Mark scheme [41] as the wind conditions (including averaged wind speed and direction, reference height, and aerodynamic roughness length), which is the compulsory requirement for green building in Singapore. Table 3 shows the average daily wind speed and direction in selected days according to the BCA Green Mark scheme [41] to evaluate green buildings in Singapore,

Formatted: Superscript

Field Code Changed

Formatted: Font: Not Bold

Field Code Changed

Formatted: Font: Not Bold

Formatted: Font: Not Bold

where the values are expected at the reference height of 15 m.

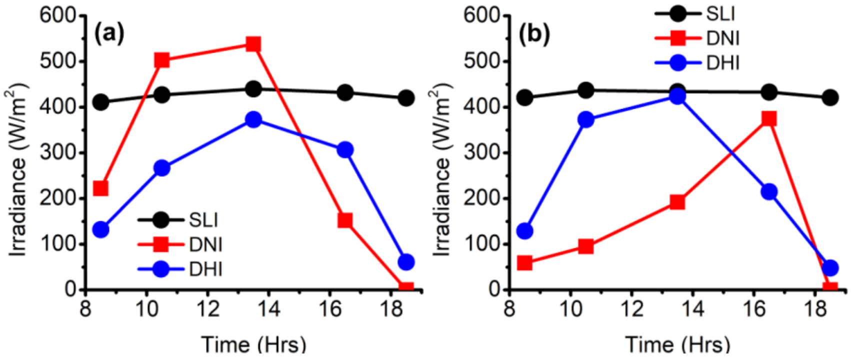

Fig 2 The selected hourly-averaged metrological data [40] of sky longwave irradiance (SLI), direct normal irradiance (DNI), and diffused horizontal irradiance (DHI) on **(a)** the equinox (sunny day) and **(b)** the solstice (cloudy day).

Table 3. Climate conditions of wind applied to this paper. The expected values follow the ~~Building and Construction Authority's (~~BCA~~)~~ Green Mark [41] at the reference height of 15 m based on the National Environment Agency's 18-year data.

Date	mean speed (m/s)	mean direction
Equinox (sunny day)	2.9	South
Solstice (cloudy day)	2.8	North-East

2.2 Integrated Environmental Modeller

The Integrated Environmental Modeller (IEM) [12, 22, 23, 24] has been developed as an integrated multi-physics urban microclimatic modeling tool for urban environment evaluation customized for the tropical climate. IEM can simulate the coupling between solar irradiance, wind flow, air temperature, and traffic noise propagation. Fig 3 shows

Formatted: Font: Not Italic

Field Code Changed

Formatted: Font: Bold

Formatted: Font: Bold

Formatted: Font: Not Bold

the workflow of IEM. Two different methods are available for calculating solar irradiance: ray-tracing and discrete ordinates models. The ray-tracing approach was used for standalone solar irradiance studies, while the discrete ordinate models were used for ~~coupled~~ wind-thermal computations to keep the computational cost low. Furthermore, the Perez all-weather sky model [12, 42] was incorporated as it has the best overall performance over a wide range of locations [43] and is one of the most suitable transposition models to predict solar irradiance for Singapore [44, 45]. Using the solar heat solver in IEM, the solar irradiance values on all surfaces in the studied system can be generated and passed to the aerodynamic simulation as input parameters.

The aerodynamic model in IEM was developed within OpenFOAM®, a finite-volume computational fluid dynamics (CFD) platform. Buoyancy effects were modeled using the Boussinesq approximation. The steady-state Reynolds-averaged Navier-Stokes (RANS) method was used for turbulence modeling to reduce the computational cost [24]. The turbulence effect was modeled using Wray-Agarwal (WA) one-equation model [24, 46, 47]. The turbulent external flows around the buildings of concern were resolved by solving the RANS equation. The Monin-Obukhov similarity theory (MOST) [48] was applied to specify the boundary conditions for wind, temperature, and turbulent viscosity. [49] All building surfaces and ground were set as non-slip walls. Atmospheric boundary layer profile for neutral flow was used at the inlet of the

computational domain. Surface-Energy-balance (SEB) model determines the heat transfer between the wind flow and the building surface. Solar shortwave radiation, thermal longwave radiation, and convective and convection heat transfer are the main mechanisms of the SEB model. The finite volume Discrete Ordinates Method (fvDOM) [50] was adopted to simulate the longwave radiation exchange between the urban surfaces and the sky. Using the aerodynamic model in IEM, the wind speed and air temperature values in the climate space can be generated and passed to noise simulation as input parameters.

The noise propagation model in IEM was developed based on the Calculation of Road Traffic Noise (CRTN) [51] coupled with the atmospheric refraction model [52] for accessing meteorological effects. Once the turbulent wind flow has been simulated using the aerodynamic model in IEM, the calculated wind speed, lapse rate, and wind shear will be passed to the atmospheric refraction model. The atmospheric refraction effects on noise propagation are calculated and considered in the CRTN model. The noise level of the area of concern due to distance attenuation, ground absorption, screening, and site layout effects can be evaluated using the CRTN model. This approach [22] allows the adoption of a set of unstructured surface mesh to represent arbitrary 3D building geometry instead of just an extrusion of a building footprint.

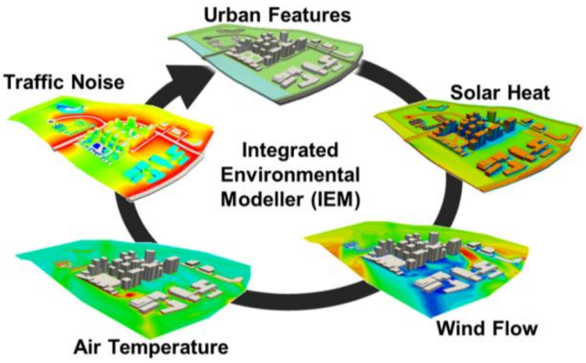

Fig 3. The workflow of coupled simulations using IEM.

2.3 Environmental comfort indices

Thermal indices have various degrees of dependence on environmental variables, including air temperature, wind speed, relative humidity, thermal radiation heat, and personal variables, including clothing, metabolic heat, mentality, and state of health. Because the empirical fits of these variables to derive thermal indices (e.g., predicted mean vote) are complicated, a more straightforward measure can be more useful in practice. Thus, operative temperature (T_O) was adopted in this study as the thermal index derived from air temperature, mean radiant temperature, and wind speed. According to the standard from ISO 7726:1998 [53], the mathematical form of T_O (℃) is written as

$$T_O = \frac{T_{air}\sqrt{10U}+T_{MRT}}{\sqrt{10U}+1}, \quad (1)$$

where T_{air} (℃) is air temperature, U (m/s) is wind speed, and T_{MRT} (℃) is mean

radiant temperature. By definition [54], T_{MRT} (℃) is the effective temperature and relates to the radiation received from all the incoming angles and can be written as

$$T_{MRT} = \sqrt[4]{\sum_k \frac{F_k}{\sigma}\left[(1-\alpha_p)Q_{Sk} + \varepsilon_p Q_{Lk}\right]} - 273.15. \quad (2)$$

where σ is Stefan-Boltzmann constant, $\alpha_p = 0.3$ is the effective albedo value for a person, and $\varepsilon_p = 0.9$ is the effective emissivity for a person. Q_{Sk} (W/m^2) is the received shortwave radiation, $Q_{Lk}(W/m^2)$ is the received longwave radiation, and F_k ~~are the received shortwave radiation, received longwave radiation, and~~is angle factor form in *k* direction. In this study, the evaluation of T_{MRT} is on the pedestrian level (i.e., 1.5 m apart from the ground).

To reveal the relationships between environment/personal variables and the human thermal comfort sensation, ASHRAE [31] defined the unitless index as the 7-point scale (–3 for cold, –2 for cool, –1 for slightly cool, 0 for neutrality, 1 for slightly warm, 2 for warm, and 3 for hot) called thermal sensation vote (TSV). Considering the average metabolic and clothing conditions in Singapore, a linear relationship [33] between TSV and T_O was derived as

$$TSV = 0.356T_O - 10.21. \quad (3)$$

According to the on-site survey in Singapore [33], the relationship between the percentage of thermal acceptability (PTA) and TSV was achieved using binary logistical regression, i.e.,

$$PTA = \frac{\exp(-1.118TSV+2.853)}{1+\exp(-1.118TSV+2.853)}. \quad (4)$$

In this study, Eq 4 was adopted to represent human acceptability with outdoor thermal conditions directly.

Noise intensity is another factor affecting human acceptability with outdoor environmental conditions. Unlike thermal comfort, the noise level is usually the only metric to define human acoustic comfort. According to the published literature [35], the logistic regression model of noise acceptance has been derived from on-site surveys and measurements in Hong Kong. Similar to Eq 4, the relationship between the percentage of noise acceptability (PNA) and noise level (L) (dB) can be written as

$$PNA = \frac{\exp(-0.55L+34.5)}{1+\exp(-0.55L+34.5)}. \quad (5)$$

Eq 5 is derived according to the equivalent continuous noise level sourced from the construction site and measured in the construction office, where the acceptable noise level is 65 dB [35]. According to the guideline from Sustainable Mobility Initiative for Local Environment (SMILE) [5], traffic noise can be regarded as the primary sound source in this study. From the National Environment Agency (NEA) [55] report in Singapore, the maximum acceptable value of traffic noise level is about 67 dB averaged over an hour. However, people's exposure to noise levels above 65 dB can cause severe health problems [5]. Considering that the noise levels >65 dB could endanger the

Formatted: Font: Not Bold

Formatted: Font: Not Bold

Formatted: Font: Not Bold

acoustic heath in urban environment [5]~~For simplicity~~, we ~~assume~~ assume that residents perceive traffic and construction noise annoyance level similarly ~~(~~~at 65 dB~~),~~ . Eq 5 was applied to predict the percentage of noise acceptability for traffic noise.

Formatted: Font: Not Bold

To evaluate the percentage of environmental acceptability within a specific region, the area-weighted average factors ($\overline{PTA}$ and $\overline{PNA}$) over the pedestrian's way can be defined

$$\overline{PTA} = \frac{1}{A_{tot}} \int_{A_{tot}} PTA \, dS, \tag{6}$$

$$\overline{PNA} = \frac{1}{A_{tot}} \int_{A_{tot}} PNA \, dS, \tag{7}$$

where dS is the area differential element and A_{tot} is the total area of the specific region. These two factors can facilitate summarizing the population the percentage of specific regions for urban planning and architectural design.

2.4 Study procedures

The procedures of this study can be summarized as shown in Fig 4. First, as discussed in Sec 2.1, the TMY weather data related to solar irradiance, the typical wind speed and direction from BCA, and the 3D STL model for the area of concern were set for input to conduct a coupled simulation for the day and time of interest. Next, radiative and thermal material properties were assigned to each group of urban surfaces (concrete, grass, roads and water body) of the 3D STL model. IEM would then automatically

Formatted: Font: Not Bold

Formatted: Font: Not Bold

generate meshes for the physical simulations. As discussed in Sec 2.2, the received shortwave irradiance (Q_S) mapping on the 3D model at a specific day and time was generated by the solar solver in IEM. Once Q_S values on the surface of 3D model have been calculated, Q_S will be taken into account in SEB model to determine the heat transfer boundary condition. Based on the heat transfer boundary condition, the meteorological quantities, air temperature (T_{air}), wind speed (U), and received longwave radiation (Q_L) were computed by the aerodynamic solver in IEM. The noise solver would calculate the noise level (L) in IEM, where U is considered in the noise simulation. Finally, as discussed in Sec 2.3, the corresponding thermal indices (T_O, T_{MRT}, and TSV) and environmental comfort acceptability (PTA and PNA) would be evaluated using Eqs 1~~, Eq 2, Eq 3, Eq 4, and Eq ~~5, respectively.

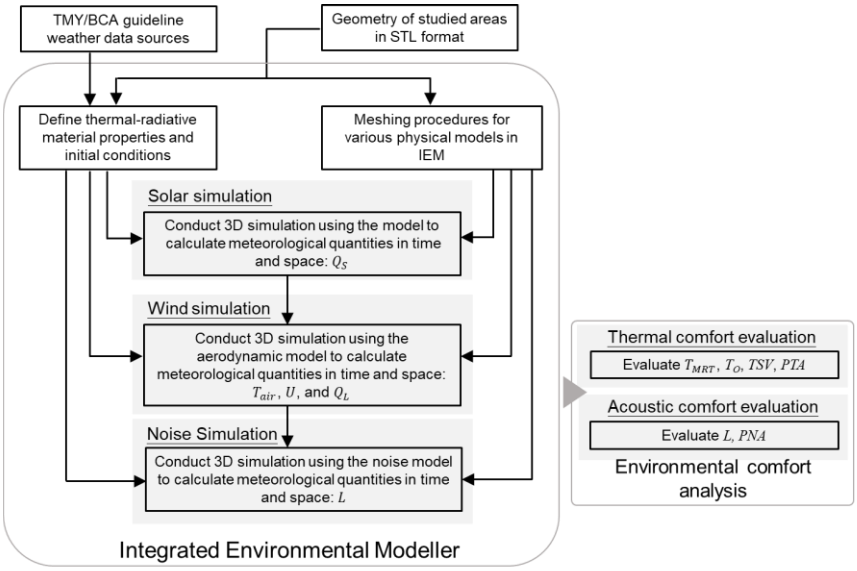

Formatted: Font: Not Bold

Formatted: Font: Not Bold

Formatted: Font: Not Bold

Formatted: Font: Not Bold

Formatted: Font: Not Bold

Formatted: Font: Not Bold

Formatted: Font: Not Bold

Formatted: Font: Not Bold

Fig 4. The flowchart represents the detailed research procedures in this study.

3. Results and discussion

3.1 Three-dimensional coupled simulations of the urban district

The size of the studied district is 2518 m × 1762 m × 84 m. For solar and noise simulations, the district's extent in STL format defines the computational domain. The meshes were generated uniformly for the 3D geometries. The resolution of the mesh is 2 meters, and the total mesh number is about 2.1 million. In wind simulations, the size of the computational domain must be greater than the original size of the district for setting corresponding inlet atmospheric boundary conditions. As discussed in Sec 2.1, the inlet wind condition is assigned according to the ~~BCA Green Mark~~ 2019 [41] shown in Table 3. According to the wind directions at different times, the computational domain sizes are 44226 m × 30976 m × 605 m for the south inlet wind direction (i.e., the sunny day) and 22057 m × 30976 m × 605 m for the north-east inlet wind direction (i.e., the cloudy day). For generating the mesh for wind simulations, snappyHexMesh utility in OpenFOAM® has been used to discretize the computational domain. The total cell count of the computational domain is 17 million and consists of a maximum mesh resolution of 8 m away from the buildings and a minimum resolution of 2 m in the region of the building. The vertical resolution is 1 m. ~~In addition, the initial reference~~

Formatted: Font: Not Bold

Formatted: Font: Not Bold

~~temperature is 30 °C.~~ According to the Davenport-Wieringa roughness classification [56] and the BCA Green Mark standard [41], the aerodynamic roughness of inflow and ground surfaces were selected as 0.03 and 1.0, respectively. We adopted the initial isothermal conditions of 30.0°C air temperatures to carry out the simulations according to the BCA Green Mark standard [41].

In this section, we present physical quantities computed using IEM on the sunny day at 10:30 and on the cloudy day at 16:30, in which the solar radiation is the most direct dominant in each day. Fig 5 shows the simulated physical quantities on the sunny day at 10:30. Fig 5~~-~~(a) is the solar irradiance mapping on the surfaces. The shadows of the buildings are on the west side due to the solar orientation in the morning. The air temperature and wind speed contours at the pedestrian level are shown in Figs 5~~Fig 5~~(b)-(~~ and Fig 5(~~c), respectively. The wind profile (Fig 5(c)) is determined by the inflow wind direction, topography, and material properties. It can be seen in Fig 5(b) that the hot spots are in the regions with abundant solar radiation ($Q_s > 600W/m^2$) and low wind speed (U < 0.5m/s). Fig 5(d) illustrates the 3D traffic noise map of the district. According to the traffic noise sources defined in Fig 1(~~b~~a) and Table 2, the color in the middle of the road is significantly darker, implying the roads have a higher noise level. This noise mapping at the district comes from calculating the noise propagation from the source and the noise reduction due to obstruction, distance attenuation, ground

Formatted: Font: 12 pt
Formatted: Font: 12 pt
Formatted: Font: 12 pt
Formatted: Font: Not Bold
Formatted: Font: Not Bold
Formatted: Font: Not Bold
Formatted: Font: Not Bold
Formatted: Font: Not Bold
Formatted: Font: Not Bold
Formatted: Font: Not Bold
Formatted: Font: Not Bold
Formatted: Font: Not Bold
Formatted: Font: Not Bold
Formatted: Font: Not Bold
Formatted: Font: Not Bold
Formatted: Font: Not Bold
Formatted: Font: Not Bold
Formatted: Font: Not Bold
Formatted: Font: Not Bold
Formatted: Font: Not Bold

absorption, and noise barrier screen. With such noise mapping and Eq 5, we can further evaluate the acoustic comfort in areas with high pedestrian traffic during peak hours and discuss this in Sec 3.2.

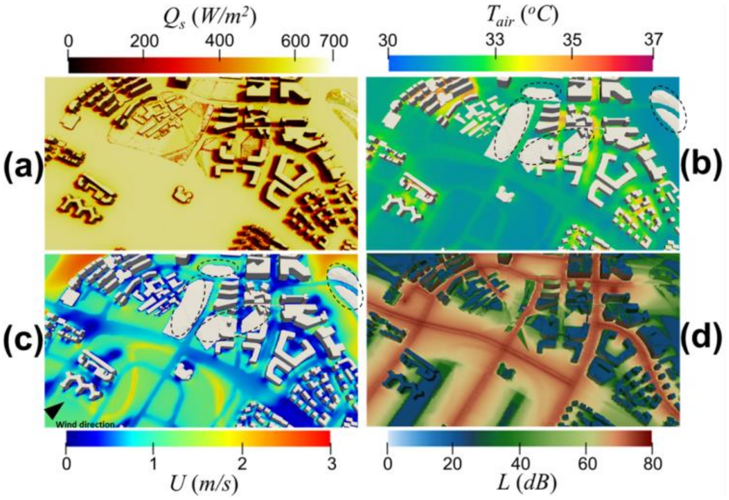

Fig 5. On the sunny day at 10:30, the figure shows **(a)** the solar irradiance mapping on the surface of the district, **(b)** air temperature as well as **(c)** wind speed contours at pedestrian level cut-plane (i.e., 1.5m) of the district, and **(d)** the noise level mapping on the surface of the district. The regions circled in **(b)** and **(c)** indicate that the terrain is higher than the 1.5m pedestrian level cut-plane. The arrow in **(~~b~~c)** denotes the wind direction.

On the other hand, Fig 6 shows the simulated physical quantities on the cloudy day at 16:30. The shadows of the buildings are on the east side due to the solar orientation in the afternoon. Fig 6(a) also shows that the amplitude of overall solar irradiance is relatively weak compared with that shown in Fig 5(a). It is reflected in the air temperature profile shown in Fig 6(b), where a decrease in global solar radiation

Formatted: Font: Not Bold

Formatted: Font: Not Bold

Formatted: Font: Not Bold

Formatted: Font: Not Bold

Formatted: Font: Not Bold

Formatted: Font: Not Bold

Formatted: Font: Not Bold

Formatted: Font: Not Bold

Formatted: Font: Not Bold

decreases the overall air temperature. Under the different inflow directions, the wind speed profile at that time is revealed in Fig 6(c). Fig 6(d) shows the corresponding noise mapping. There is no noticeable difference between Figs 5Fig 5(d) and and Fig 66(d) (d) because the temperature gradient and wind shear impact on traffic noise propagation of both cases is relatively low for the area and tropical climate under study.

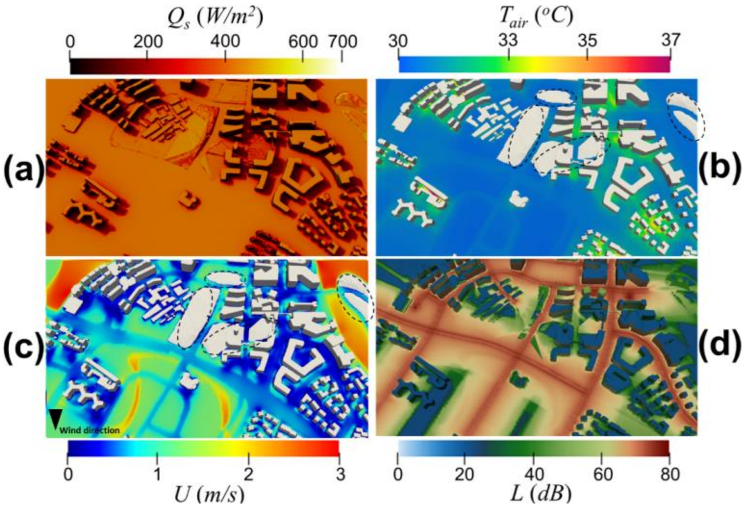

Fig 6. On the cloudy day at 16:30, the figure shows **(a)** the solar irradiance mapping on the surface of the district, **(b)** air temperature as well as **(c)** wind speed contours at pedestrian level (i.e., 1.5-m) of the district, and **(d)** the noise level mapping on the surface of the district. The regions circled in **(b)** and **(c)** indicate that the terrain is higher than the 1.5m pedestrian level cut-plane. The arrow in **(c)** denotes the wind direction.

3.2 Evaluation and analysis of thermal comfort on the study sites

To further evaluate the environmental comfort in the area with high pedestrian traffic are marked in Fig 1(cb), Fig 7 shows the thermal comfort index (i.e., TSV) and acoustic

Formatted: Font: Not Bold
Formatted: Font: Not Bold
Formatted: Font: Not Bold
Formatted: Font: Not Bold
Formatted: Font: Not Bold
Formatted: Font: Not Bold
Formatted: Font: Not Bold
Formatted: Font: Not Bold
Formatted: Font: Not Bold
Field Code Changed

comfort index (i.e., L) contours at the pedestrian level (i.e., 1.5 m). The TSV results are calculated using Eq 3 based on the simulation results shown in Fig 5(a)-(c) and Figs 6~~Fig 6~~(a)-(c). Fig 7(a) shows that TSV >2 in more than 80% of the area. The outdoor environment is considered hot on the sunny day at 10:30. In contrast, TSV on a cloudy day at 16:30, shown in Fig 7(c), is relatively low (<1) for >60% of the area of interest. The high TSV generally happens due to high solar irradiance, and due to the weak wind flow that is usually associated with a low wind speed of ≤ 2 m/s (see Fig 6(c)). Thus, in Figs 7~~Fig 7~~(a) and 7(c) ~~(c)~~, TSV hotspots occur in areas with great solar exposure and inadequate natural ventilation, strongly affected by street geometry, building orientation, wind direction, and sun position. For example, in Fig 7(a), the hotspot can be observed in the south area of the site (iii), where it is unshaded and lacks the speed for adequate permeability of natural ventilation on the sunny day 10:30. In contrast, in Fig 7(c), the reduction of TSV in the same place is because solar irradiance, sun position, and wind direction change on the cloudy day 16:30. The contour of the noise level (L) shown in Figs 7~~Fig 7~~(b) and 7~~Fig 7~~(d) are similar, indicating that the effect of wind and thermal shear are weak for the area of interest. These results in Figs 7~~Fig 7~~(b) and 7(d) are derived from the IEM outputs as shown in Figs 5~~Fig 5~~(d) and ~~Fig 6~~6(d).

Formatted: Font: Not Bold
Formatted: Font: Not Bold
Formatted: Font: Not Bold
Formatted: Font: Not Bold
Formatted: Font: Not Bold
Formatted: Font: Not Bold
Formatted: Font: Not Bold
Field Code Changed
Field Code Changed
Formatted: Font: Not Bold
Formatted: Font: Not Bold
Formatted: Font: Not Bold
Field Code Changed
Formatted: Font: Not Bold
Formatted: Font: Not Bold
Formatted: Font: Not Bold
Field Code Changed
Formatted: Font: Not Bold
Field Code Changed
Formatted: Font: Not Bold
Formatted: Font: Not Bold
Formatted: Font: Not Bold
Field Code Changed
Formatted: Font: Not Bold
Formatted: Font: Not Bold
Field Code Changed
Formatted: Font: Not Bold
Formatted: Font: Not Bold
Formatted: Font: Not Bold
Formatted: Font: Not Bold
Formatted: Font: Not Bold
Formatted: Font: Not Bold
Formatted: Font: Not Bold

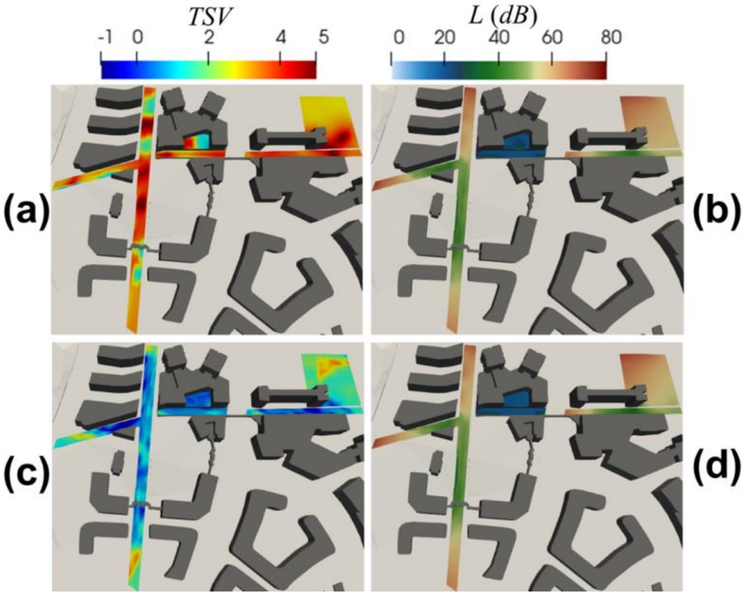

Fig 7. According to the simulation results shown in Fig 5 and Fig 6, the figure shows contours of comfort indices of **(a)** TSV and **(b)** noise level at the sunny day 10:30, and **(c)** TSV and **(d)** noise level at the cloudy day 16:30.

Environmental acceptability as a people-centric way to evaluate thermal comfort for architecture and urban design, compared to using typical thermal comfort metrics (such as TSV) is also evaluated here. Fig 8(a) shows the relationship between the percentage of thermal acceptability (PTA) and TSV calculated following Eq 4. Similarly, Fig 8(b) shows the relationship between the percentage of noise acceptability (PNA) and noise level according to Eq 5. The thresholds for thermal and acoustic comfort metrics are defined as 50% of the population feeling acceptable at TSV = 2.3 and L = 62.6 dB, respectively. The qualified evaluation of environmental comfort at a specific location is achievable based on whether the PTA and PNA are over 0.5. It is noted that the

Formatted: Font: Not Bold

Formatted: Font: Not Bold

Formatted: Font: Not Bold

Formatted: Font: Not Bold

Formatted: Font: Not Bold

Formatted: Font: Not Bold

Formatted: Font: Not Bold

threshold (PTA = 0.5 and PNA = 0.5) used in this study is an estimate of the feelings of the simple majority rather than the universal definition. From different studies [57, 58, 59], other environmental comfort thresholds were introduced based on different environmental matrices for the specific purpose.

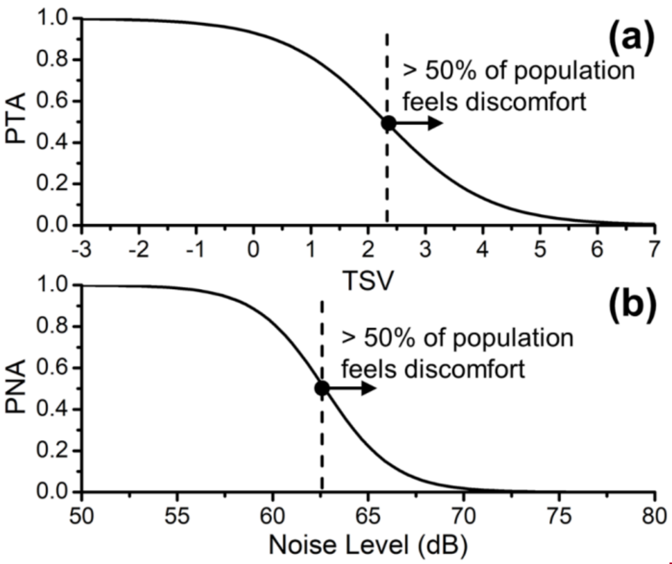

Fig 8. shows **(a)** the relationship between the percentage of thermal acceptability (PTA) and thermal sensation vote (TSV) obtained from Eq 4, and **(b)** the relationship between the percentage of noise acceptability (PNA) and noise level obtained from Eq 5, respectively. The dashed lines denote the index threshold (PTA = 0.5 and PNA = 0.5) of that over 50% population feels discomfort (i.e., the right area pointed by the arrow).

Formatted: Font: Not Bold

Formatted: Font: Not Bold

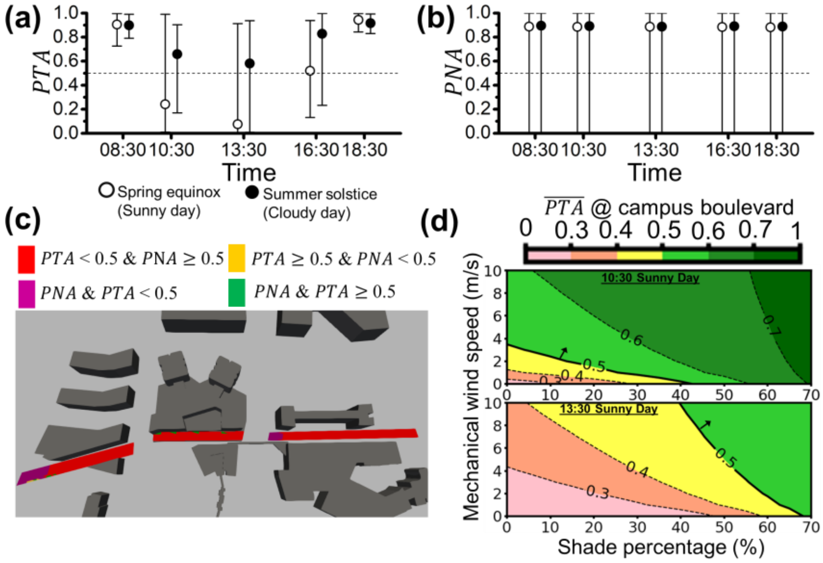

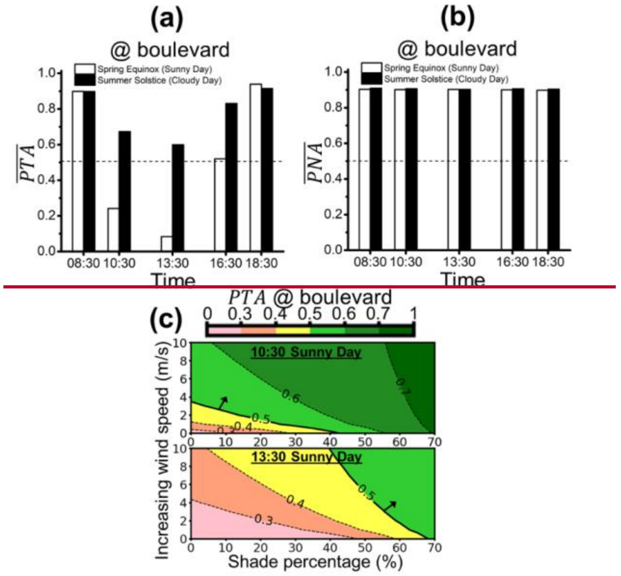

Fig 9. The area-averaged **(a)** $\overline{PTA}$ and **(b)** $\overline{PNA}$ of the campus boulevard (site (i)) for both sunny (open circles) and cloudy (filled circles) days are represented with open and filled circles (i.e., $\overline{PTA}$ and $\overline{PNA}$), respectively. The error bars extended from the area-averaged $\overline{PTA}$ or $\overline{PNA}$ (circles) represent the local maximum and local minimum PTA or PNA on the campus boulevard. The environmental acceptability threshold of 0.5 is denoted by the dotted line (i.e., $\overline{PTA} = 0.5$ or

Formatted: Font: Not Bold
Formatted: Font: Not Bold
Formatted: Font: Not Bold
Formatted: Font: Not Bold
Formatted: Font: Not Bold
Formatted: Font: Not Bold
Formatted: Font: Not Bold
Formatted: Font: Not Bold
Formatted: Font: Not Bold
Formatted: Font: Not Bold
Formatted: Font: Not Bold
Formatted: Font: Not Bold
Formatted: Font: Not Bold
Formatted: Font: Not Bold
Formatted: Font: Not Bold
Formatted: Font: Not Bold
Formatted: Font: Not Bold
Formatted: Font: Not Bold

$PNA =$ 0.5). **(c)** The spatial plot of PTA and PNA above or below the threshold of 0.5 for campus boulevard at 13:30 on the sunny day (worst case) is illustrated. **(d)** The contour plot shows the potential improvement of PTA with the increase in shade and/or wind speed at 10:30 and 13:30 on the sunny day for the campus boulevard. On campus boulevard (site (i)), **(a)** the hourly-averaged $\overline{PTA}$ of the both the sunny day (white column) and the cloudy day (black column) are shown. **(b)** The hourly-averaged $\overline{PNA}$ of the both the sunny day (white column) and the cloudy day (black column) are shown. The dot line in the figures denote the baseline of environmental acceptability (i.e., $\overline{PTA} = 0.5$ and $\overline{PNA} = 0.5$) in this study. **(c)** At the time points while the $\overline{PTA} < 0.5$ in **(a)**, the contour plot shows an improvement of $\overline{PTA}$ by increasing shade percentage and by accelerating wind speed where the upper/lower panel represents the improved $\overline{PTA}$ at 10:30/13:30 of the sunny day.

From Figs 9Fig 9–12to **Fig 12**, we evaluate the environmental comfort at each site (i, ii, iii, and iv) within the area with high pedestrian traffic are marked in Fig 1(ba). Fig 9(a) shows the hourlyarea--averaged PTA– (i.e., $\overline{PTA}$) of the sunny day and the cloudy day on the east-west boulevard. The cloudy day has higher $\overline{PTA}$s than the sunny day during the daytime, consistent with the trend of solar irradiance shown in Fig 2. The error bars illustrate the huge variation between local maximum and minimum PTA at different locations along the campus boulevard. The local variation of PTA strongly depends on the extent of the shade projected by the surrounding buildings at that hour of interest. There are two points in time (10:30 and 13:30) that $\overline{PTA}$ is below 0.5 on the sunny day. – and the corresponding minimum PTA is very close to 0. We expect these two points to be under a relatively high global solar irradiance, and >50% of people are uncomfortable with the thermal environment. In contrast, Fig 9(b) shows that the area-averaged PNA (i.e. $\overline{PNA}$s) isare almost always close to a constant (~0.9)

Formatted: Font: Not Bold
Formatted: Font: Not Bold
Formatted: Font: Not Bold
Formatted: Font: Not Bold
Formatted: Font: Not Bold
Formatted: Font: Not Bold
Formatted: Font: Not Bold
Formatted: Font: Not Bold
Formatted: Font: Not Bold
Formatted: Font: Not Bold
Formatted: Font: Not Bold
Formatted: Font: Not Bold
Field Code Changed
Formatted: Font: Not Bold
Field Code Changed
Formatted: Font color: Auto
Formatted: Font color: Auto
Field Code Changed
Formatted: Font: Not Bold

that far exceeds the baseline ($\overline{PNA}$ = 0.5) all the time during peak hours, even though the local minimum PNA is close to 0. In Fig 9(c), the spatial distribution of PTA and PNA at 13:30 on the sunny day (worst case) is illustrated. It is obvious that most of the area along the campus boulevard is expected to be thermally uncomfortable with $PTA < 0.5$ (red and purple zones), and the spaces, where $PNA < 0.5$, are concentrated near the two road junctions (i.e., traffic noise sources defined in Fig 1(a)), which overlaps with a part of the $PTA < 0.5$ region, marked in purple~~.~~. The small zones with, $PTA < 0.5$ and $PNA > 0.5$; and; $PTA > 0.5$ and $PNA > 0.5$ along the bottom edge of the purple zone with $PTA < 0.5$ are marked in amber and green, respectively. Th~~e fact~~ is indicates that traffic noise level is always acceptable by most people even at peak hour traffic except for people who stay in the small region directly affected by the traffic noise. Local noise mitigation measures are required in such the areas if open air retail spaces are planned in the purple and amber regions, where $PNA < 0.5$.~~, even at peak hour traffic.~~

For improving overall environmental comforts on the specific site for high-level retail planning, the averaged indicators (i.e., $\overline{PTA}$ and $\overline{PNA}$) rather than local values (i.e., PTA and PNA) will be further analyzed. As compared to $\overline{PNA}$ shown in Fig 9(b), the improvement of $\overline{PTA}$ is the primary objective to be achieved by architectural solutions. We provide a simplified parametric analysis to improve thermal comfort at the hot times

Formatted: Font color: Auto
Field Code Changed
Formatted: Font color: Auto
Formatted: Font color: Auto
Formatted: Font color: Auto
Formatted: Font color: Auto
Formatted: Font color: Auto
Formatted: Font: Not Bold, Font color: Auto
Formatted: Font: Not Bold, Font color: Auto
Formatted: Font color: Auto
Formatted: Font color: Auto
Formatted: Font color: Auto
Formatted: Font color: Auto
Formatted: Font color: Auto
Formatted: Font: Not Bold
Field Code Changed

while $\overline{PTA}$ < –0.5 (i.e., 10:30 and 13:30 of the sunny day) by decreasing solar irradiance and increasing the wind speed from the simulation results, and other conditions held constant. Tuning the two parameters may be done using setting up shelters to block the incoming solar radiation and adding ventilation fans to increase the local wind speed under ideal conditions. The contour plot in Fig 9(d) shows the results of the improvement of $\overline{PTA}$ by decreasing the solar irradiance and increasing the wind speed. Increasing shade percentage (i.e., decreasing solar irradiance) and increasing wind speed are set from 0 to 70% and 0 to 10 m/s, respectively. The increasing wind speed of 10 m/s is the maximum boost provided by typical outdoor ventilation fans [60]. The solid lines in Fig 9(d) indicate the threshold of $\overline{PTA}$ = –0.5 to separate the region with $\overline{PTA}$ > –0.5 (green colors) and the region with $\overline{PTA}$ < –0.5 (warm colors). The intersections of the solid line and vertical and horizontal axes represent the minimum requirements for the adopting the single way (only increasing wind speed and only increasing shade percentage) to achieve $\overline{PTA}$ = –0.5. At 10:30 of the sunny day (the upper panel of Fig 9(d)), $\overline{PTA}$ = –0.5 can be achieved by increasing 3.7 m/s in wind speed or blocking 42 % of solar irradiance. The corresponding value of wind speed and shade percentage on the solid line also denotes the minimum coupling mechanism criterion to achieve $\overline{PTA}$ = 0.5. For instance, we can improve $\overline{PTA}$ to 0.5 by increasing 2 m/s in wind speed and blocking 15% of solar irradiance simultaneously. In

Formatted: Font: Not Bold
Field Code Changed

Formatted: Font: Not Bold
Field Code Changed

Field Code Changed
Formatted: Font: Not Bold

the lower panel of Fig 9(ed), it is observed that the further increase in wind speed and shade percentage is required to reach the $\overline{PTA}$ baseline under a higher global solar irradiance at 13:30 of the sunny day as compared to that at any other time. $\overline{PTA}$ = 0.5 can be achieved by blocking 68 % of solar irradiance but cannot be achieved by only increasing the wind speed of <-10 m/s.

Field Code Changed

Formatted: Font: Not Bold

Formatted: Space Before: 6 pt, Line spacing: Double

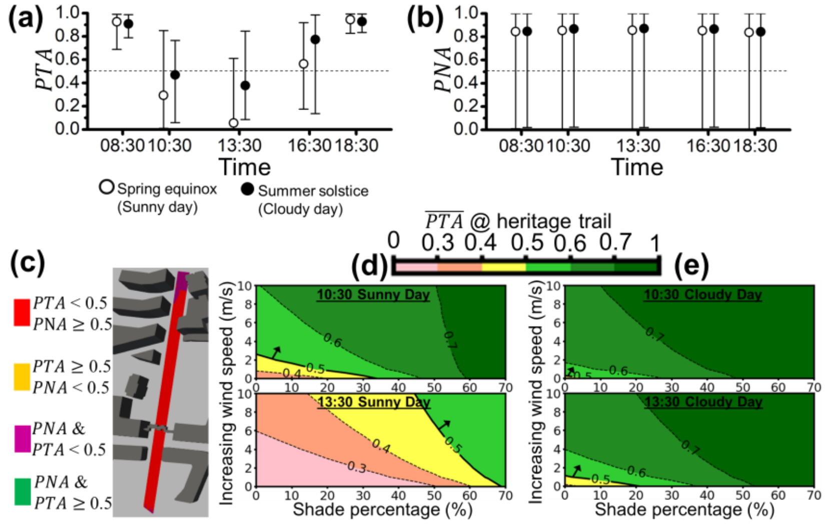

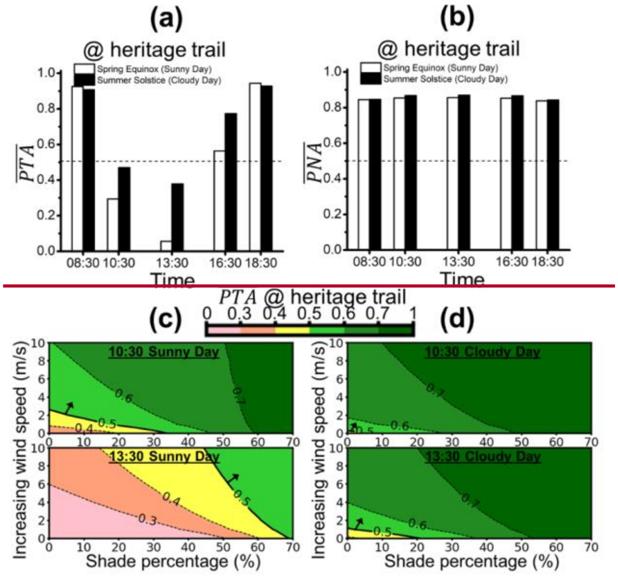

Fig 10. The area-averaged **(a)** $\overline{PTA}$ and **(b)** $\overline{PNA}$ of the heritage trail (site (ii)) for both sunny (open circles) and cloudy (filled circles) days are represented with open and filled circles (i.e., $\overline{PTA}$ and $\overline{PNA}$), respectively. The error bars extended from the area-averaged PTA or PNA (circles) represent the local maximum and minimum PTA or PNA on the heritage trail. The environmental acceptability threshold of 0.5 is denoted by the dotted line (i.e., $PTA =$ 0.5 or $PNA =$ 0.5). **(c)** The spatial plot of PTA and PNA above or below the threshold of 0.5 for heritage trail at 13:30 on the sunny day (worst case) is illustrated. The contour plot shows the potential improvement of $\overline{PTA}$ with the increase in shade and/or wind speed at 10:30 and 13:30 on **(d)** the sunny day, and **(e)** the cloudy day for the heritage trail. ~~On heritage trail (site (ii)), **(a)** the hourly-averaged $\overline{PTA}$ of the both the sunny day (white column) and the cloudy day (black column) are shown. **(b)** The hourly-averaged $\overline{PNA}$ of the both the sunny day (white column) and the cloudy day (black column) are shown. The dot line in figures denote the baseline of environmental acceptability (i.e., $\overline{PTA}$ = 0.5 and $\overline{PNA}$ = 0.5) in this study. At the time points while the $\overline{PTA}$ < 0.5 in **(a)**, the contour plot shows an improvement of $\overline{PTA}$ by increasing shade percentage and by accelerating wind speed where the upper/lower panel represents the improved $\overline{PTA}$ at 10:30/13:30 of **(c)** the sunny day, and **(d)** the cloudy day.~~

Fig 10(a) and 10(b) show the ~~hourly~~area--averaged $\overline{PTA}$ and $\overline{PNA}$ of the sunny day

Formatted: Font: 12 pt, Font color: Auto

Formatted: Font: 18 pt

Formatted: Font: 12 pt, Font color: Auto

Formatted: Caption, Centered

Formatted: Font: Not Bold

Formatted: Font: Not Bold

Formatted: Font: Not Bold

and the cloudy day along the heritage trail. Unlike Fig 9(a), Fig 10(a) shows that $\overline{PTA}$s are less than 0.5 with the corresponding minimum $PTA \cong 0$ –at 10:30 and ~~11~~13:30 of the cloudy day. In Fig 10(b), $\overline{PTA}$s are between 0.84 and 0.88, slightly less than that shown in Fig 9(b), but the corresponding minimum PNAs are close to 0 all the time. Fig 10(c) shows that most of the area of the heritage trail is exposed to the thermal problem with $PTA < 0.5$ (red and purple zones) at 13:30 on sunny day. At the same time, the space overlapped the area with $PNA < 0.5$ (purple zones) is located at the part of the north end of the heritage trail. The green ($PTA < 0.5$ and $PNA > 0.5$) and amber ($PTA > 0.5$ and $PNA > 0.5$) zones are almost none existed. Fig 10(c)~~, and~~ (d) ~~(d)~~ show the improvement of $\overline{PTA}$ via two ways on the sunny and cloudy day. At 10:30 of the sunny day (the upper panel of Fig 10(c)), using increasing 2.6 m/s in wind speed or blocking 32 % of solar irradiance can reach $\overline{PTA} = 0.5$. As can be seen in the lower panel of Fig 10(c), $\overline{PTA} = 0.5$ can be achieved by blocking at least 68% of the incoming solar irradiance at 13:30 of the cloudy day, but the same $\overline{PTA}$ value cannot be achieved by only increasing the wind speed of < 10 m/s. On the other hand, as shown in Fig 10(d), we can improve $\overline{PTA}$ to reach 0.5 via adding 6.7% shade percentage or increasing 0.3 m/s in wind speed at 10:30 of the cloudy day. $\overline{PTA} = 0.5$ can be achievable by adding 21% shade percentage or increasing 1.1 m/s in wind speed at 13:30 of the cloudy day.

Formatted: Font: Not Bold
Formatted: Font: Not Bold
Formatted: Font: Not Bold
Field Code Changed
Formatted: Font: Not Bold
Formatted: Font: Not Bold
Formatted: Font: Not Bold
Field Code Changed
Formatted: Font: Not Bold
Formatted: Font: Not Bold
Formatted: Font: Not Bold
Formatted: Font: Not Bold
Formatted: Font: Not Bold
Formatted: Font: Not Bold
Formatted: Font: Not Bold
Formatted: Font: Not Bold
Formatted: Font: Not Bold
Formatted: Font: Not Bold
Formatted: Font: Not Bold
Formatted: Font: Not Bold

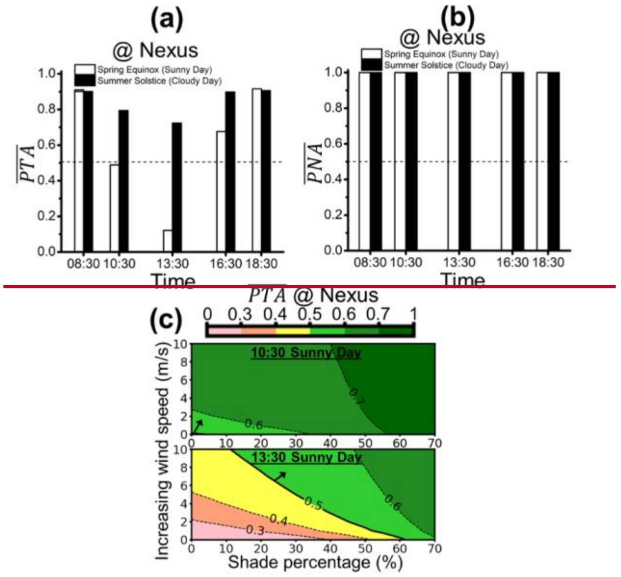

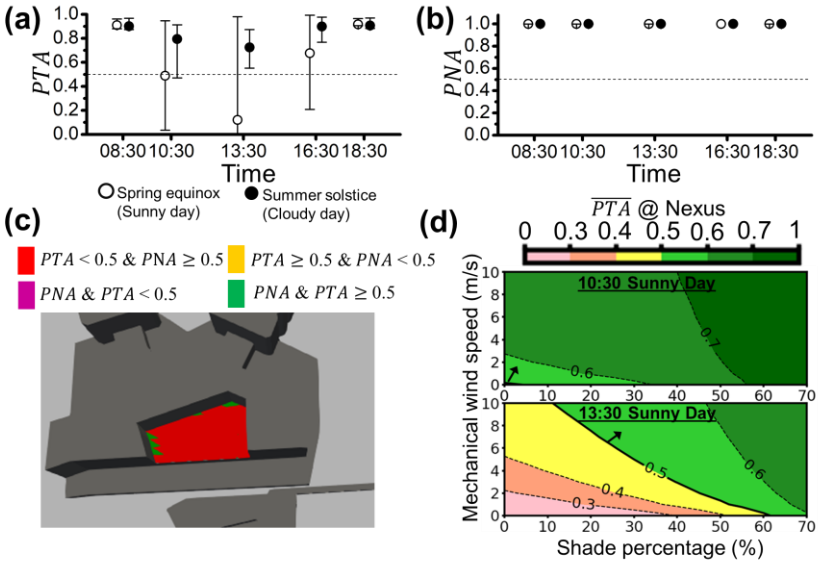

Fig 11. The area-averaged **(a)** PTA and **(b)** PNA of Nexus (site (iii)) for both sunny (open circles) and cloudy (filled circles) days are represented with open and filled circles (i.e., $\overline{PTA}$ and $\overline{PNA}$), respectively. The error bars extended from the area-averaged PTA or PNA (circles) represent the local maximum and local minimum PTA or PNA on Nexus. The environmental acceptability threshold of 0.5 is denoted

by the dotted line (i.e., $\overline{PTA} =$ 0.5 or $\overline{PNA} =$ 0.5). **(c)** The spatial plot of $\overline{PTA}$ and $\overline{PNA}$ above or below the threshold of 0.5 for Nexus at 13:30 on the sunny day (worst case) is illustrated. **(d)** The contour plot shows the potential improvement of $\overline{PTA}$ with the increase in shade and/or wind speed at 10:30 and 13:30 on the sunny day for Nexus.~~At Nexus (site (iii)), **(a)** the hourly-averaged $\overline{PTA}$ of the both the sunny day (white column) and the cloudy day (black column) are shown. **(b)** The hourly-averaged $\overline{PNA}$ of the both the sunny day (white column) and the cloudy day (black column) are shown. The dot line in figures denote the baseline of environmental acceptability (i.e., $\overline{PTA}$ = 0.5 and $\overline{PNA}$ = 0.5) in this study. **(c)** At the time points while the $\overline{PTA}$ < 0.5 in **(a)**, the contour plot shows an improvement of $\overline{PTA}$ by increasing shade percentage and by accelerating wind speed where the upper/lower panel represents the improved $\overline{PTA}$ at 10:30/13:30 of the sunny day.~~

~~Fig 11~~Fig 11(a)~~ and~~ (b) show the ~~hourly~~area-~~-~~averaged $\overline{PTA}$ and $\overline{PNA}$ of the sunny day and the cloudy day at the Nexus. In Fig 11(a), $\overline{PTA}$s are higher than that shown in Fig 9(a) and Fig 10(a), especially on the cloudy day. However, the minimum $\overline{PTA}$ is still close to 0 on the sunny day, e.g., at 13:30 (worst case). From Fig 11(b), $\overline{PNA}$ is always close to 1 with almost zero variation –that 100% of people satisfy the acoustic environment all the time during peak hours. The spatial feature of PTA and PNA at 13:30 on the sunny day as shown in Fig 11(c) can be seen that the most of area is with $PTA < 0.5$ marked in red, few scattered areas with $PTA \geq 0.5$ are marked in green, and almost no area is with $PNA > 0.5$. Fig 11(~~c~~d) shows the improvement of $\overline{PTA}$ via two strategies on the sunny day. At 10:30 of the sunny day (the upper panel of Fig 11(~~c~~d)), merely using increasing 0.2 m/s in wind speed or blocking 4.2 % of solar irradiance can reach $\overline{PTA}$ = 0.5. In the lower panel of Fig 11(~~c~~d), the baseline of $\overline{PTA}$

Formatted: Font: Not Bold
Formatted: Font: Not Bold
Formatted: Font: Not Bold
Formatted: Font: Not Bold
Formatted: Font: Not Bold
Field Code Changed
Formatted: Font: Not Bold
Formatted: Font: Not Bold
Formatted: Font: Not Bold
Formatted: Font: Not Bold
Formatted: Font: Not Bold
Formatted: Font: Not Bold
Formatted: Font: Not Bold
Formatted: Font: Not Bold
Formatted: Font: Not Bold
Formatted: Font: Not Bold
Formatted: Font: Not Bold
Formatted: Font: Not Bold
Formatted: Font: Not Bold
Formatted: Font: Not Bold

can be achievable by blocking 62% of solar irradiance at 13:30 of the sunny day but still be unachievable by solely increasing the wind speed.

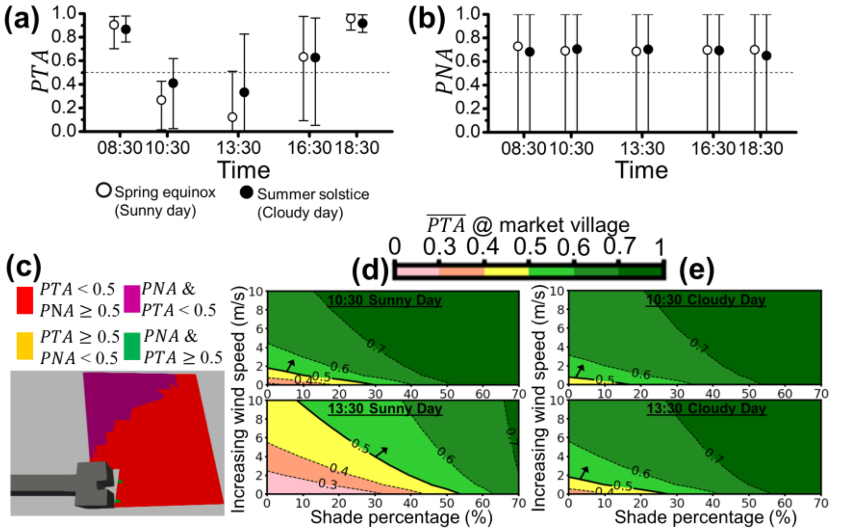

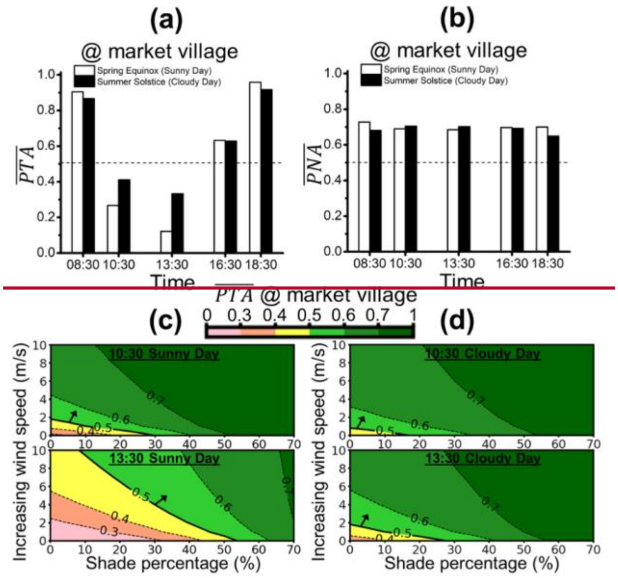

Fig 12 The area-averaged **(a)** $\overline{PTA}$ and **(b)** $\overline{PNA}$ of the market village (site (iv)) for both sunny (open circles) and cloudy (filled circles) days are represented with open

and filled circles (i.e., $\overline{PTA}$ and $\overline{PNA}$), respectively. The error bars extended from the area-averaged PTA or PNA (circles) represent the local maximum and minimum PTA or PNA on the campus boulevard. The environmental acceptability threshold of 0.5 is denoted by the dotted line (i.e., $PTA =$ 0.5 or $PNA =$ 0.5). **(c)** The spatial plot of PTA and PNA above or below the threshold of 0.5 for market village at 13:30 on the sunny day (worst case) is illustrated. The contour plot shows the potential improvement of PTA with the increase in shade and/or wind speed at 10:30 and 13:30 on **(d)** the sunny day, and **(e)** the cloudy day for the market village.At the market village (site (iv)), **(a)** the hourly-averaged $\overline{PTA}$ of the both the sunny day (white column) and the cloudy day (black column) are shown. **(b)** The hourly-averaged $\overline{PNA}$ of the both the sunny day (white column) and the cloudy day (black column) are shown. The dot line in figures denote the baseline of environmental acceptability (i.e., $\overline{PTA}$ = 0.5 and $\overline{PNA}$ = 0.5) in this study. At the time points while the $\overline{PTA}$ < 0.5 in **(a)**, the contour plot shows an improvement of $\overline{PTA}$ by increasing shade percentage and by accelerating wind speed where the upper/lower panel represents the improved $\overline{PTA}$ at 10:30/13:30 of **(c)** the sunny day, and **(d)** the cloudy day.

Figs 12(a)-(b) Fig 12 (a) and (b) show the hourlyarea-averaged $\overline{PTA}$ and $\overline{PNA}$ with the corresponding variation of the sunny day and the cloudy day at the market village. Similar to the results (see Fig 9(a)), $\overline{PNTA}$s are less than 0.5 and the corresponding minimum $PTAs$ are close to 0 at 10:30 and 1113:30 of the sunny and cloudy day as shown in Fig 12(a). It can be observed that the maximum PTAs at 10:30 and 13:30 of the sunny and cloudy day are much less than those elsewhere. On the other, in Fig 12(b), $\overline{PNA}$s are between 0.69 and 0.73, slightly less than that shown in the results from Figs 9(b), 10(b), and 11(b) Fig 9(b), Fig 10(b), and Fig 11(b). The corresponding minimum $PNAs$ are close to 0 all the time. Fig 12(c) shows that the zone with $PTA < 0.5$ (red and purple) is almost everywhere and a part of space overlapped with $PNA < 0.5$

Formatted: Caption, Centered
Formatted: Font: Not Bold
Formatted: Font: Not Bold
Formatted: Font: Not Bold
Field Code Changed
Formatted: Font: Not Bold
Formatted: Font: Not Bold
Formatted: Font: Not Bold
Formatted: Font: Not Bold
Formatted: Font: Not Bold
Formatted: Font: Not Bold
Formatted: Font: Not Bold
Field Code Changed
Formatted: Font: Not Bold
Formatted: Font: Not Bold
Formatted: Font: Not Bold
Formatted: Font: Not Bold
Formatted: Font: Not Bold
Formatted: Font: Not Bold
Formatted: Font: Not Bold

(purple) appears beside the noise sources. Figs 12(d)-(e) Fig 12 (c) and (d) show the improvement of $\overline{PTA}$ via two strategies on the sunny and cloudy day. At 10:30 of the sunny day (the upper panel of Fig 10(cd)), using increasing 1.8 m/s in wind speed or blocking 29 % of solar irradiance, we can achieve $\overline{PTA}$ = 0.5. In the lower panel of Fig 12(cd), $\overline{PTA}$ = 0.5 can be achieved at 13:30 of the sunny day by blocking 54 % of solar irradiance. On the other hand, as shown in Fig 10(de), we can improve $\overline{PTA}$ to reach 0.5 via adding a 17% shade percentage or increasing 0.8-m/s in wind speed at 10:30 of the cloudy day. $\overline{PTA}$ = 0.5 is achievable via adding 27.5% shade percentage or increasing 1.8-m/s wind speed at 13:30 of the cloudy day. The overall conditions to achieve $\overline{PTA}$ = 0.5 from Figs 9(d), 10(d), 10(e), 11(d), 12(d), and 12(e) are summarized in Table 4.

Table 4. The table summarizes the conditions to achieve $\overline{PTA}$ = 0.5 from Figs 9(d), 10(d), 10(e), 11(d), 12(d), and 12(e). In each cell, the upper panel is the required shade percentage, and the upper panel is the required increasing wind speed. The cells in green represent that $\overline{PTA} \geq 0.5$ can be naturally achieved. The cells in red represent that cannot be achieved by only cannot be achieved by only increasing the wind speed in a reasonable range (< 10 m/s).

		campus boulevard site (i)	heritage trail site (ii)	Nexus site (iii)	market village site (iv)
sunny day	10:30	42 %<br>3.7 m/s	32 %<br>2.6 m/s		29 %<br>1.8 m/s
	13:30	68%<br>>10 m/s	68 %<br>>10 m/s	4.2 %<br>0.2 m/s	54 %<br>>10 m/s
cloudy day	10:30		6.7 %<br>0.3 m/s		17 %<br>0.8 m/s
	13:30		21 %<br>1.1 m/s		27.5 %<br>1.8 m/s

Formatted: Font: Not Bold
Formatted: Font: Not Bold
Formatted: Font: Not Bold
Formatted: Font: Not Bold
Formatted: Font: Not Bold
Formatted: Font: Not Bold
Formatted: Font: Not Bold
Formatted: Font: Not Bold
Formatted: Font: Not Bold
Formatted: Font: Not Bold

3.3 Discussion

From ~~Fig 9~~Fig 9~~ to Fig 12~~12, $\overline{PTA}$ and $\overline{PNA}$ directly represent the overall human acceptance of outdoor environmental conditions in a specific area. The varia~~nce~~tion of PTA and PNA are affected by the local environmental conditions. At each time, the maximum or minimum PTA and PNA represent the most acceptable or unacceptable extent locally for thermal and acoustic comfort in different studied sites, respectively. The results (Figs 9(b), 10(b), 11(b), and 12(b)~~Fig 9(b), Fig 10(b), Fig 11(b), and Fig 12(b)~~) show that $\overline{PNA}$s do vary slightly (between 0.69 and 1) for the five different time slots due to the different wind and air temperature conditions for all the four sites in the business district. The site with more surrounding buildings, which act as acoustic barriers, has higher $\overline{PNA}$. Thus, the site (iii) (Nexus) has the highest $\overline{PNA}$ (= 1) among four sites and the site (iv) (market village) has the lowest $\overline{PNA}$ (~ 0.7). Although $\overline{PNA}$s is in the range between 0.7 and 1, the corresponding minimum PNA is close to 0 except for site (iii) (Nexus). From the results (Figs 9(c), 10(c), and 12(c)), the local zones with a small PNA are always near the noise sources. Among four sites in this study, the north-west region of site (iv) is exposed to the most serious traffic noise because this region is surrounded by noise sources without any acoustic barriers, like buildings. The straightforward way to improve the acoustic comfort by

Formatted: Font: Not Bold
Field Code Changed
Formatted: Font: Not Bold
Field Code Changed
Field Code Changed
Field Code Changed
Formatted: Font: Not Bold
Formatted: Font: Not Bold
Formatted: Font: Not Bold
Formatted: Font: Not Bold
Formatted: Font: Not Bold
Formatted: Font: Not Bold
Field Code Changed
Formatted: Font: Not Bold
Formatted: Font: Not Bold
Formatted: Font: Not Bold

constructing local acoustic barriers to protect pedestrians from the traffic noise. However, the acoustic barriers will block airflow transport, further endangering thermal comfort. Thus, outdoor retail activities would not be recommended in the purple zones ($PNA < 0.5$ and $PTA < 0.5$) as shown in Figs 9(c), 10(c), and 12(c). For the four study sites, PNA < 0.5 is found in the small area near the traffic noise sources. PNA is always greater than 0.5 for most of the spaces in the four sites. In contrast, PTA < 0.5 for most of the spaces is always observed in the worst-case scenario as shown in Figs 9(c), 10(c), 11(c), and 12(c). For high-level retail planning, $\overline{PTA}$ and $\overline{PNA}$ are representative metrics. $\overline{PNA}$ is always far beyond 0.5 in each site, which means no immediate action is required to mitigate traffic noise if the retail places are not near the planned roads in the business district.In general, $\overline{PNA}$ is always far beyond 0.5 in each site, which means no immediate action is required to mitigate traffic noise from the planned roads in the business district. Considering Singapore's relatively low wind speed (< 3 m/s), solar irradiance becomes the essential parameter affecting $\overline{PTA}$. The results (Figs 9(a), 10(a), 11(a), and Fig 12(a)Fig 9(a), Fig. 10(a), Fig 11(a), and Fig 12(a)) show a high correlation between $\overline{PTA}$ and solar irradiance, as shown in Fig 2. As such, $\overline{PTA}$ is observed reaching the minimum at noon of both days, where the global solar irradiance is the maximum each day. To ensure that $\overline{PTA}$ can at least reach the baseline of 0.5 all the time, we evaluate the improving $\overline{PTA}$ at the hot times (Figs

Formatted: Font: Not Bold
Formatted: Font: Not Bold
Formatted: Font: Not Bold
Formatted: Font: Not Bold
Field Code Changed
Formatted: Font: Not Bold
Formatted: Font: Not Bold
Field Code Changed
Formatted: Font: Not Bold
Formatted: Font: Not Bold
Field Code Changed

9(d), 10(d), 10(e), 11(d), 12(d) and 12(e)~~Fig 9(c), Fig 10(c), Fig 10(d), Fig 11(c), Fig 12(c) and Fig 12(d)~~) by increasing wind speed, blocking solar irradiance, or adopting above both simultaneously. In these contour plots, the solid line separates the parametric space to achieve $\overline{PTA} \geq 0.5$ (i.e., the green-colored region pointed by the arrow in contour plots) and $\overline{PTA} < 0.5$ (i.e., the warm-colored region). The intersections of the solid lines and vertical axis and horizontal axes represent the minimum requirements for adopting the only increasing wind speed and only blocking solar irradiance to achieve $\overline{PTA} = 0.5$. The parametric space of the required conditions to achieve $\overline{PTA} = 0.5$ at 13:30 (the lower panel of Figs 9(d), 10(d), 11(d), and 12(d)~~Fig 9-12 (c)~~) of the sunny day is much smaller than that at any other time (the upper panel of Figs 9(d), 10(d), 10(e), 11(d), 12(d), and 12(e)~~Fig 9-12 (c), Fig 10(d), and Fig 12(d)~~). The corresponding required conditions for achieving $\overline{PTA} \geq 0.5$ at each site at 13:30 (the worst-case scenario) can be applied to achieve $\overline{PTA} \geq 0.5$ at any other time. For example, in the lower panel of Fig 10(~~c~~d), the requirements for achieving $\overline{PTA} \geq 0.5$ also satisfy that as shown in the upper panel of Fig 10(~~c~~d) and Fig 10(~~d~~e).

If considering using the single strategy for improving $\overline{PTA}$ at the hottest time (i.e., 13:30 of the sunny day as shown in the lower panel of Figs 9(d), 10(d), 11(d), and 12(d)~~Fig 9(c), Fig 10(c), Fig 11(c), Fig 12(c)~~, blocking the solar irradiance is an efficient way to achieve $\overline{PTA} = 0.5$ when the solar radiation is the direct dominant with a small

Formatted: Font: Not Bold
Field Code Changed
Formatted: Font: Not Bold
Formatted: Font: Not Bold
Formatted: Font: Not Bold
Formatted: Font: Not Bold
Formatted: Font: Not Bold
Formatted: Font: Not Bold
Formatted: Font: Not Bold
Field Code Changed
Formatted: Font: Not Bold
Formatted: Font: Not Bold
Field Code Changed
Formatted: Font: Not Bold
Formatted: Font: Not Bold
Field Code Changed
Formatted: Font: Not Bold
Formatted: Font: Not Bold
Field Code Changed
Formatted: Font: Not Bold
Formatted: Font: Not Bold
Formatted: Font: Not Bold
Formatted: Font: Not Bold
Field Code Changed
Formatted: Font: Not Bold
Formatted: Font: Not Bold
Formatted: Font: Not Bold
Formatted: Font: Not Bold
Formatted: Font: Not Bold
Formatted: Font: Not Bold
Formatted: Font: Not Bold
Field Code Changed
Formatted: Font: Not Bold
Formatted: Font: Not Bold
Formatted: Font: Not Bold
Formatted: Font: Not Bold
Formatted: Font: Not Bold
Formatted: Font: Not Bold
Field Code Changed
Formatted: Font: Not Bold
Formatted: Font: Not Bold

zenith angle. Although the layout of surrounding buildings is different at study sites, the improvement trends of $\overline{PTA}$ are similar, and $\overline{PTA}$ at all four sites can be improved from <0.2 to 0.5 by blocking 54-68% of solar irradiance, respectively. It should be noted that $\overline{PTA}$ =0.5 at the site (iv) (market village) can be achieved by blocking 54%, which is the minimum among the four sites. This fact means that blocking solar irradiance is more efficient to improve $\overline{PTA}$ in open spaces that agree with the findings from [61, 62, 63, 64]. Increasing wind speed is another way to improve $\overline{PTA}$. However, the results at 13:30 of the sunny day (i.e., the lower panel of Figs 9(d), 10(d), 11(d), and 12(d) ~~Fig 9(c), Fig 10(c), Fig 11(c), Fig 12(c)~~) show that $\overline{PTA}$ is always smaller than 0.5 even though the wind speed is increased by 10 m/s, which is the typical maximum wind speed for mechanical ventilation [60]. In contrast, the results at any other times when 0.3< $\overline{PTA}$ <0.5 show that $\overline{PTA}$ ≥0.5 is achievable by increasing the wind speed to 0.2-3.7 m/s. As stated above, we can deduce that blocking solar irradiance can efficiently improve $\overline{PTA}$ at any time on both days, especially when $\overline{PTA}$ <0.2 (at ~~11~~13:30 of the sunny day). On the other hand, enhancing ventilation by increasing wind speed is effective at the time when $\overline{PTA}$ >0.3 (e.g., the cloudy day) in the first place.

Furthermore, the combined effects of blocking solar irradiance and increasing wind speed can be a more efficient way to improve $\overline{PTA}$ than just adopting shade or boosting the wind. In the contour plots from Figs 9-12 ~~Fig 9 to Fig 12~~, every point on

Formatted: Font: Not Bold
Formatted: Font: Not Bold
Formatted: Font: Not Bold
Formatted: Font: Not Bold
Formatted: Font: Not Bold
Field Code Changed
Formatted: Font: Not Bold
Field Code Changed
Formatted: Font: Not Bold
Formatted: Font: Not Bold

Formatted: Font: Not Bold
Field Code Changed
Formatted: Font: Not Bold

the solid line represents a combination of conditions to reach $\overline{PTA}$ = 0.5. For instance, the lower panel of Figs 9(d), 10(d), 11(d), and 12(d) ~~Fig 9(c), Fig 10(c), Fig 11(c), and Fig 12(c)~~ show that the required blocking percentage of solar irradiance can be decreased ~10% for reaching $\overline{PTA}$ ≥0.5 at ~~11~~13:30 of the sunny day when the wind speed is increased by 2 m/s which is roughly conventional fan airspeed driven by ceiling fans [65]. In practicality, this way provides flexibility in architecture and has been widely used around al fresco restaurants, outdoor amphitheaters, and some sheltered pedestrian ways in Singapore.

The main implication of the result is that $\overline{PTA}$ and $\overline{PNA}$ give an indication on the percentage of visitors who find the location comfortable enough for them to patronize or hang around al fresco dining and pop-up retail kiosks in the outdoor environment. In this study, $\overline{PTA}$ is a dominant factor to determine people's willingness to retail queue or gather at all four sites at the hot time. On the other hand, although the $\overline{PNA}$ is always >0.5 at each site, traffic noise issue in the area with $PNA < 0.5$ still should be considered in the retail planning. From the spatial color mapping plots (Figs 9(d), 10(d), 11(d), and 12(d)), the regions with $PNA < 0.5$ are always associated with $PTA < 0.5$, marked in purple. This clearly defines the zone that is not recommended for outdoor retails if the thermal and acoustic comfort cannot be improved simultaneously. According to the results, specific guidelines for retail planning for each

Formatted: Font: Not Bold

Formatted: Font: Not Bold

Formatted: Font: Not Bold

Formatted: Font: Not Bold

Field Code Changed

Formatted: Font: Not Bold

Formatted: Font: Not Bold

Field Code Changed

Formatted: Font: Not Bold

Formatted: Font: Not Bold

site can be made as follows. At site (i) (campus boulevard) where is a thoroughfare with buildings on either side, $\overline{PTA}$ can be expected to improve by installing pop-up shopping areas next to the side buildings and awnings for visitor queuing. To increase local airspeed using outdoor ventilation devices will further improve $\overline{PTA}$. In comparison between the results, Figs 9(a) and 10(a)~~Fig 9(a) and Fig 10(a)~~, site (ii) (heritage trail) is hotter than site (i) due to lacking buildings on the side to block the solar irradiance. The building shadows can directly improve the thermal comfort [66, 67]. The site (ii) is planned to be a pedestrian-only pathway renovated from an old road. Thus, $\overline{PTA}$ here can be expected to improve by installing a sheltered walkway [68] and planting street trees with overhead coverage [54, 69, 70]. T~~Moreover, outdoor activities for retail purpose~~he location of retail shops or kiosk that may attract long queues~~, *e.g.*, visitor queuing,~~ are not recommended in the purple zones (Figs 9(c) and 10(c)) near the road junctions at the site (i) and (ii). The site (iii) (Nexus) has a higher $\overline{PTA}$ benefiting from the spontaneous shading by three-sides surrounding buildings. With sufficient shading percentage by the shelter and increased wind speed via ceiling fans suggested by Fig 11(~~c~~d), the tenants or visitors are expected to be more willing to stay in the outdoor area for al fresco dining [71] and pop-up shopping over a long time. In addition, ~~form **Fig 11(c)**,~~ site (iii) is the only one for outdoor activities free from interfering traffic noise in this study as seen in Fig 11(c). The site (iv) (market village)

Formatted: Font: Not Bold

Formatted: Font: Not Bold

Formatted: Font: Not Bold

Formatted: Font: Not Bold

Field Code Changed

Formatted: Font: Italic

Formatted: Font: Not Bold

Formatted: Font: Not Bold

is an open area for outdoor activities. According to the results from Fig 12(~~e~~d), $\overline{PTA}$ can be efficiently improved by blocking solar irradiance via installing the canopy frames and planting trees. ~~Moreover~~Keeping away from the area affected by traffic noise, the purple zone in Fig 12(c), outdoor amphitheaters with ceiling fans have a suitable setting for outdoor events.

If the shading techniques and mechanical wind devices may be challenging to deploy practically to reach the condition for $\overline{PTA}$ improvement, people may tend to remain indoors by relying on air conditioning during the hot time. On the other hand, outdoor activities become inappropriate in the area exposed the traffic noise, i.e., purple zones in Figs 9(c), 10(c), and 12(c). Because of that, urban planners and architects can choose retail options at outdoor locations for specific purposes. We have pointed out that the threshold of $\overline{PTA}$ = 0.5 and $\overline{PNA}$ = 0.5 is the baseline used in this study but not universal. The policy of the $\overline{PTA}$ and $\overline{PNA}$ standards can be formulated depending on the actual circumstances, e.g., $\overline{PTA}$ ≥ 0.6. According to ~~the~~Table 4 (the results from Figs 9(d), 10(d), 10(e), 11(d), 12(d), and 12(e)~~Fig 9(c), Fig 10(c), Fig 10(d), Fig 11(c), Fig 12(c), and Fig 12(d)~~), the architects and urban planners can find the corresponding conditions for improving $\overline{PTA}$ locally.

We note that this study had only examined the potential improvement on $\overline{PTA}$ via the simplified parametric analysis (i.e., decreasing the solar irradiance and increasing the

Formatted: Font: Not Bold
Field Code Changed
Formatted: Font: Not Bold
Field Code Changed
Formatted: Font: Not Bold
Formatted: Font: Not Bold
Formatted: Font: Not Bold
Formatted: Font: Not Bold
Formatted: Font: Not Bold
Formatted: Font: Not Bold
Formatted: Font: Not Bold
Formatted: Font: Not Bold
Field Code Changed
Formatted: Font: Not Bold
Formatted: Font: Not Bold
Formatted: Font: Not Bold
Field Code Changed
Formatted: Font: Not Bold
Formatted: Font: Not Bold

wind speed) under ideal conditions, without considering trees, shades, or outdoor fans in the simulations. Under the ideal conditions, we assumed that the solar radiation level and wind speed have implicitly incorporated the effects of trees, shades, or outdoor fans. The double parametric space in terms of solar radiation and wind speed provides a guideline for architects to quickly identify the optimal microclimate conditions for planning the infrastructure and landscape combination during the scenario design stage. The exact details of the surrounding environment, i.e., shades, trees and fans, can be inversely designed based on the optimal microclimate condition, which is out of the scope of this paper and requires further study. Although the evaluation provides the parametric scope and the options to improve outdoor thermal comfort for future plan of combining landscape and infrastructure (e.g., trees with shelter walkaways, green walls with outdoor ventilation fans, and etc.) in design, the solar-wind-coupled simulations and on-site measurements with regards the as-built remains to be done at a later stage.

4. Conclusions

In conclusion, this paper evaluates the outdoor environmental comfort of an emerging district in Singapore. This evaluation was based on Integrated Environmental Modeler's (IEM) simulation results, a 3D coupled physics simulation platform that includes solar radiation, wind, thermal, and traffic noise, using a single unified set of geometrical input. For the architectural purpose, we selected two representative days in the hottest period

of Singapore (i.e., from March to June), which are the spring equinox (sunny day) and the summer solstice (cloudy day), respectively. The sunny day can be regarded as the worst-case scenario and the cloudy day is the reference scenario to conduct simulations considering the material properties of infrastructure and terrain features. TMY data and the BCA Green Mark standards were adopted in solar and wind simulations as the weather conditions in solar and wind simulations. The traffic noise sources were categorized based road characteristics (such as the number of lanes and average speed of the vehicles during the peak hour) and applied in the noise simulations to represent the worst-case traffic noise scenario. From the results of the IEM, we further derived environmental indices (i.e., TSV and L) on the four studied sites with high pedestrian traffic. These four sites are either pedestrian thoroughfares, or gathering areas for potential outdoor community events and activities for the tenants and residents staying near the business district. The people-centric acceptability factors (i.e., PTA and PNA), defined as a function of TSV and L, were introduced to define the degrees of environmental comfort. The area-averaged factors $\overline{PTA}$ and $\overline{PNA}$ with the corresponding variance were applied to evaluate each studied site. The variance of PTA and PNA come from the spatial distribution of environmental conditions in this business district. In the worst-case scenario, i.e., at 13:30 on the sunny day, $PTA < 0.5$ is observed almost everywhere along all sites. In the meantime, the places, $PNA < 0.5$,

Formatted: Font color: Text 1

are found concentrated near the noise sources, which overlaps with a part of the $PTA <$ 0.5 region. These areas (purple zones in Figs 9(c), 10(c), and 12(c)) are not recommended for outdoor activities for retail purposes because improving PNA by constructing the noise barriers may further degrade the local PTA. For the high-level retail planning, wWe set the acceptable criterion of $\overline{PTA}$ = 0.5 and $\overline{PNA}$ = 0.5, meaning most people would be satisfied with the thermal and acoustic environment, respectively. $\overline{PNA}$ is always far beyond 0.5 in the studied sites, which means that most people here are satisfied with the acoustic comfort influenced by the proximity and road categories in the area except for people stay in purple zones as shown in Figs 9(c), 10(c), and 12(c). In contrast, $\overline{PTA}$ is highly dependent on global solar irradiance due to the low-wind-speed conditions which is always observed < 3 m/s in this study. Thus, $\overline{PTA}$ trends towards the minimum at noon of the sunny day when global solar irradiance is maximum. We proposed a simplified parametric analysis to improve the $\overline{PTA}$ when $\overline{PTA}$ < 0.5, (1) by blocking the solar irradiance and (2) by increasing the wind speed. The improvement of $\overline{PTA}$ is a function of the shading percentage and the mechanical wind speed. Considering only a single strategy is adopted, blocking solar irradiance is efficient to improve $\overline{PTA}$ at 13:30 of the sunny day, i.e., the hottest time. $\overline{PTA}$ rises from <0.2 to 0.5 can be achieved by blocking 54%-68% solar irradiance among the four sites. However, when $\overline{PTA}$ <0.3, the improvement over baseline is difficult to achieve

Formatted: Font color: Text 1

Formatted: Font color: Text 1

Formatted: Font color: Text 1

by enhancing the local wind speed by up to 10m/s, which is the maximum boost provided by typical ventilation fans. Enhancing the mechanical wind speed is effective to improve $\overline{PTA}$ at the time when $\overline{PTA}$ >0.3 (e.g., the cloudy day). Coupling the shading and boosting wind can be a more efficient way to improve $\overline{PTA}$. For example, increasing a 2 m/s wind speed requires a smaller shelter that blocks ~10% less solar irradiance as compared to a shading only solution to maintain $\overline{PTA}$ ≥0.5. This combination provides more flexibility for architecture based on site constraints. Moreover, the quantitative analysis of $\overline{PTA}$ and ~~PNA~~ the well-defined ~~difficult~~uncomfortable region, purple zones as ~~shown~~~~deplicted~~depicted in Figs 9(c), 10(c), and 12(c), can help develop strategies to improve environmental comfort to encourage outdoor gatherings and establish the reference of outdoor retail options for each site. Considering the surrounding building layouts and the $\overline{PTA}$ improvement at each study site, potential outdoor retail options were suggested. For instance, placement of pop-up kiosks with outdoor queues and al fresco dining options are preferred for areas where the thermal and acoustic acceptability are higher than the benchmark (i.e., 50%) most of the time. Based on the assessment, the $\overline{PTA}$ can be set as the target for specific purposes, instead of the baseline of $\overline{PTA}$ = 0.5. If the shading techniques and mechanical wind devices are challenging to deploy to reach the target of $\overline{PTA}$ practically or the area exposed to the traffic noise issue at the same time, then air-

Formatted: Font color: Text 1

Formatted: Font color: Text 1

conditioned stores and restaurants can be set up. To summarize the work, the assessment based on coupled-physics simulations can guide architects and urban planners to take the appropriate retail option for practical purposes of the district. Furthermore, according to the assessment of environmental acceptability, the people-centric and context-specific plans (such as outdoor comfort and retail planning) can be evaluated, and suitable infrastructure or mitigation measures can be put in place, to enable the planned activities even if the location might not be sufficiently comfortable (i.e., low $\overline{PTA}$) or for the planned activity in the first place. Possible topics for further exploration include solar-wind-noise coupled simulations, calibrated against on-site measurements, of the scenarios with future landscape and infrastructure combinations. (e.g., trees with shelter walkaways, green walls with outdoor ventilation fans etc.) In addition, this work could be extended to include key performance indicators (KPIs), such as building energy consumption and renewable energy options~~, to achieve the district-scale sustainability goal~~.

~~Acknowledgement~~

~~This research is supported by A*STAR under its URBAN & GREENTECH OFFICE (UGTO) CONSULTANCY FUND (Grant No.: UGTO/CF/01). This development work was undertaken as a part of the project "Modeling of Air Flow, Thermal and Chemical Gas Dispersion Towards Next Generation Port (Tuas Maritime Hub)", funded by the~~

Formatted: Default, Space Before: 6 pt, After: 6 pt

~~Singapore Maritime Institute (SMI-2016-MA-04). The conclusions put forward reflect the views of the author alone, and not necessarily those of the funding institution.~~

Author Contributions

Conceptualization: Po-Yen Lai, Wee Shing Koh.

Data curation: Po-Yen Lai, Dias Leong, Hyosoo Lee.

Formal analysis: Po-Yen Lai.

Funding acquisition: Wee Shing Koh.

Investigation: Po-Yen Lai, Wee Shing Koh, Harish Gopalan.

Methodology: Po-Yen Lai, Harish Gopalan, Huizhe Liu, Wee Shing Koh, Dias Leong, Hyosoo Lee, Gibert Peh, Gilbert Cher, Cheng Hui Eng, Jia Li Goh.

Project administration: Wee Shing Koh, Johnathan Goh.

Resources: Edmund Tan, James Tan.

Software: Harish Gopalan, Huizhe Liu, Dias Leong, Hyosoo Lee, Jacob Ang.

Supervision: Wee Shing Koh, Edmund Tan, James Tan.

Validation: Po-Yen Lai.

Visualization: Po-Yen Lai, Dias Leong, Jiun Yeu Lim, Hyosoo Lee.

Writing – original draft: Po-Yen Lai.

Writing – review & editing: Wee Shing Koh, Harish Gopalan, Huizhe Liu, Dias Leong, Hyosoo Lee, Johnathan Goh, Jiun Yeu Lim, Jacob Ang, Gibert Peh, Gilbert Cher,

Cheng Hui Eng, Jia Li Goh, Edmund Tan, James Tan.

References

1. Meteorological Service Singapore, Past Climate Trends; 2021 [cited 2021 Jun 17] Available from: http://www.weather.gov.sg/climate-past-climate-trends/.
2. Watkins R, Palmer J, Kolokotroni M. Increased Temperature and Intensification of the Urban Heat Island: Implications for Human Comfort and Urban Design. Built Environment. 2007;33(1):85-96.
3. Wong NH, Yu C. Study of green areas and urban heat island in a tropical city. Habitat International. 2005;29:547-558.
4. Hung T, Uchihama D, Ochi S, Yasuoka Y. Assessment with satellite data of the urban heat island effects in Asian mega cities. International Journal of Applied Earth Observation and Geoinformation, 2008,8:31-48,.
5. SMILE - Guidelines for Road Traffic Noise Abatement; 2003 [cited 2022 Jan 18] Available from: https://webgate.ec.europa.eu/life/publicWebsite/project/details/1780#:~:text=%22S%20M%20I%20L%20E%20%2D%20Guidelines%20for%20Road%20Traffic%20Noise%20Abatement%22%20(2%20MB).
6. Kousis I, Pisello AL. For the mitigation of urban heat island and urban noise island: two simultaneous sides of urban discomfort. Environmental Research Letters. 2020,103004,15.
7. Chin C, Thang ZY, Saju S. Study on impact of noise annoyance from highway traffic in Singapore City, in 178th Meeting of the Acoustical Society of America: Architectural Acoustics: Paper 1aAA6, San Diego, California, 2019.
8. Tumini I, Higueras García E, Baereswyl Rada S. Urban microclimate and thermal comfort modelling: strategies for urban renovation. International Journal of Sustainable Building Technology and Urban Development. 2016,7(1),22-37.
9. Yu X, Zhang Q, Kang J, Cui F. Predicting integrated thermal and acoustic performance in naturally ventilated high-rise buildings using CFD and FEM simulation. Building Simulation. 2018,11(3),507-518,.

10. Kanda M. Progress in urban meteorology: a review. J Meteorol Soc Jpn. 2007,85B,363-383.

11. Toparlar Y, Blocken B, Maiheu B, Heijstd GJFV. A review on the CFD analysis of urban microclimate. Renewable and Sustainable Energy Reviews. 2017,80,1613-1640.

12. Wang B, Koh WS, Liu H, Yik J, Bui VP. Simulation and validation of solar heat gain in real urban environments. Building and Environment. 2017,123,261-276.

13. Allegrini J, Dorer V, Carmeliet J. Coupled CFD, radiation and building energy model for studying heat fluxes in an urban environment with generic building configurations. Sustainable Cities and Society. 2015,19,385-394.

14. Qu Y, Milliez M, Musson-Genon L, Carissimo B. Numerical study of the thermal effects of buildings on low-speed airflow taking into account 3D atmospheric radiation in urban canopy. Journal of Wind Engineering and Industrial Aerodynamics, 2012,104,474-483.

15. Roth M, Lim VH., Evaluation of canopy-layer air and mean radiant temperature simulations by a microclimate model over a tropical residential neighbourhood. Building and Environment. 2017,112,177-189.

16. Israelsson S, Sound Propagation in the Atmosphere near the Ground, Swedish: University of Uppsala, 1979.

17. Hornikx M, Dohmen M, Conen K, van Hooff T, Blocken B. The wind effect on sound propagation over urban areas: Predictions for generic urban sections. Building and Environment. 2018,144,519-531,.

18. Wagner S, Bareiß R, Guidati G. Wind Turbine Noise, Berlin, Heidelberg: Springer, 1996.

19. Matzarakis A, Rutz F, Mayer H. Modelling radiation fluxes in simple and complex environments: basics of the RayMan model. Int J Biometeorol. 2010,54(2),131-139.

20. Lindberg F, Holmer B, Thorsson S. 1.0-Modeling spatial variations of 3-D radiant fluxes and mean radiant temperature in complex urban settings. Int J Biometeorol. 2008,52(7),697-713.

21. Bruse M and Fleer H Simulating surface-plant-air interactions in urban environments with a three dimensional numerical model. Environ. Modell. Softw. 1998, 13, 374-384.

22. Zhao WJ, Liu EX, Poh HJ, Wang B, Gao SP and Png CE. 3D traffic noise mapping using unstructured surface mesh representation of buildings and roads. Applied Acoustics. 2017,127,297-304.

23. Wise D, Boppana V, Li K, Poh H, Effects of Minor Changes in the Wind Direction on Urban Flow Simulations. Sustainable Cities and Society. 2018,37,492-500.
24. Gopalan H. Evaluation of Wray-Agarwal turbulence model for simulation of neutral and non-neutral atmospheric boundary layers. Journal of Wind Engineering and Industrial Aerodynamics. 2018,182, 322-329.
25. Höppe P. Different aspects of assessing indoor and outdoor thermal comfort. Energy and Buildings. 2002,34(6),661-665.
26. Chen L, Ng E. Outdoor thermal comfort and outdoor activities: a review of research in the past decade. Cities. 2012,29(2),118-125.
27. Höppe P, The physiological equivalent temperature – a universal index for the biometeorological assessment of the thermal environment, International Journal of Biometeorology. 1999,43,71-75.
28. Gagge AP, Stolwijk JAJ, Hardy JD. Comfort and thermal sensations and associated physiological responses at various. Environmental Research. 1967,1,1-20.
29. Błażejczyk K, Jendritzky G, Bröde P, Fiala D, Havenith G, Epstein Y, et al. An introduction to the Universal Thermal Climate Index (UTCI). Geographia Polonica. 2013,86(1),5-10.
30. Fanger PO. Thermal Comfort. Copenhagen: Danish Technical Press. 1970.
31. ASHRAE, ASHRAE Standard 55-2004. Thermal environmental conditions for human occupancy, American Society of Heating, Refrigerating and Air-Conditioning Engineering, Atlanta, Georgia, USA. 2004.
32. Zaki N, Rijal S, Ahmad H, Razak N and Razak A. Field study of pedestrians' comfort temperatures under outdoor and semi-outdoor conditions in Malaysian university campuses Othman. International Journal of Biometeorology. vol. 2021,65,453-477,.
33. Yang W, Wong NH, Jusuf SK. Thermal comfort in outdoor urban spaces in Singapore. Building and Environment. 2013,59,426-435,.
34. Berglund B, Lindvall T, Schwela DH. Guidelines for Community Noise. London, United Kingdom: World Health Organization. 1999.
35. Mui K, Wong L, Wong Y. Acceptable noise levels for construction site offices. Building Serv. Eng. Res. Technol. 2009,30(1),87-94,.
36. Garg N, Maji S, A critical review of principal traffic noise models: Strategies and implications. Environmental Impact Assessment Review. 2014,46,68-81,.

37. Department of Transport, Welsh Office. Calculation of Road Traffic. United Kingdom: HSMO Publication. 1988.
38. Authority LT. Road Line Plan Explanatory Notes; [cited 2022 Jan 19] Available from: https://www.lta.gov.sg/content/dam/ltagov/industry_innovations/industry_matters/development_construction_resources/road_line_plan/pdf/Road_line_Plan_Explanatory_notes_final.pdf.
39. Climate of Singapore, Meteorological Service Singapore; 2021 [cited 2021 Aug 13] Available from: http://www.weather.gov.sg/climate-climate-of-singapore/.
40. ASHRAE. International Weather for Energy Calculations (IWEC Weather Files) Users Manual and CD-ROM, Atlanta: ASHRAE. 2001.
41. Green Mark for Non-Residential Buildings NRB: 2015, [cited 2021 Jan 6] Available from: https://www.bca.gov.sg/greenmark/others/Green_Mark_NRB_2015_Criteria_Draft_R3.pdf.
42. Perez R, Seals R, Michalsky J. All-weather model for sky luminance distribution-Preliminary configuration and validation. Solar Energy. 1993,50(3),235-245,.
43. Robinson D, Stone A. A simplified radiosity algorithm for general urban radiation exchange. Build. Serv. Eng. Res. Technol. 2005,26(4),271-284.
44. Lam KP, Mahdavi A, Ullah M, Ng E, Pal V. Comparative study of sky luminance models in the tropical context. Building Simulation. 1997,1,339.
45. Yang D. Solar radiation on inclined surfaces: Corrections and benchmarks. Solar Energy. 2016,136(15),288-302,.
46. Han X, Wray T, Agarwal R. Application of a New DES Model Based on Wray-Agarwal Turbulence Model for Simulation of Wall-Bounded Flows with Separation, in AIAA 2017-3966, Session: Applications of Fluid Mechanics, 47th AIAA Fluid Dynamics Conference, Denver, Colorado. 2017.
47. Rumsey C, Smith B, Huang G. Description of a website resource for turbulence modeling verification and validation. in Session: FD-32: Turbulent Flows: RANS and Hybrid Methods, 40th Fluid Dynamics Conference and Exhibit, Chicago, Illinois. 2010.
48. Monin A, Obukhov A. Basic laws of turbulent mixing in the surface layer of the atmosphere. Contrib. Geophys. Inst. Acad. Sci. USSR. 1954,151(163),187.
49. Foken T. 50 years of the Monin–Obukhov similarity theory. Boundary-Layer Meteorology. 2006,119(3),431-447.

50. Raithby GD, Chui EH. A Finite-Volume Method for Predicting a Radiant Heat Transfer in Enclosures With Participating Media. Journal of Heat Transfer. 1990,112(2),415-423.
51. Calculation of road traffic noise. Her Majesty's Stationery Office, London. 1988.
52. Zhao WJ, Boppana VBL, Liu EX, Poh HJ, Li KW, Tan TT. Traffic with Noise Modeling Consideration of Meteorological Effect. in INTER-NOISE and NOISE-CON Congress and Conference Proceedings, Hamburg GERMANY. 2016.
53. ISO 7726:1998 Ergonomics of the thermal environment -- Instruments for measuring physical quantities, Organization, International Standard. 1998.
54. Lai PY, Koh JH, Koh WS, Liu H. Effectively modeling surface temperature and evaluating mean radiant temperature in tropical outdoor industrial environments. Building and Environment. 2020,169,106277.
55. Hock KK. Environmental Protection Division Report. National Environmental Agency, Singapore; 2012, [cited 2021 May 31] Available from: https://www.nea.gov.sg/docs/default-source/resource/publications/environmental-protection-division-annual-report/epd-2011.pdf.
56. Wieringa J. Updating the Davenport roughness classification. Journal of Wind Engineering and Industrial Aerodynamics. 1992,41(1),357-368.
57. Nouri AS, Costa JP, Santamouris M, Matzarakis A. Approaches to Outdoor Thermal Comfort Thresholds through Public Space Design: A Review. Atmosphere, 2018,9(3),108.
58. Yang W, Wong NH, Lin Y. Thermal Comfort in High-rise Urban Environments in Singapore. Procedia Engineering. 2015,121, 2125-2131.
59. Malik A, Bongers C, McBain B, Rey-Lescure O, Dear Rd, et al. The potential for indoor fans to change air conditioning use while maintaining human thermal comfort during hot weather: an analysis of energy demand and associated greenhouse gas emissions. Lancet Planet Health. 2022,6,E301-E309.
60. Silva W, Moura D, Carvalho-Curi T, Seber R, Massari J. Evatuation system of exhaust fans used on ventilation system in commercial broiler house. Journal of the Brazilian Association of Agricultural Engineering. 2017,5(37),887-899.
61. Shashua-Bar L, Ioannis X, Milo H. Passive cooling design options to ameliorate thermal comfort in urban streets of a Mediterranean climate (Athens) under hot summer conditions. Build. Environ. 2012,57,110-119.

62. Cheung P, Jim C. Comparing the cooling effects of a tree and a concrete shelter using PET and UTCI. Build. Environ. 2018,130,49-61.
63. Nasrollahi N, Ghosouri A, Khodakarami J, Taleghani M. Heat-Mitigation Strategies to Improve Pedestrian Thermal Comfort in Urban Environments: A Review. Sustainability, 2020,12,10000.
64. Zaki SA, Syahidah S, Shahidan MF, Ahmad MI, Yakub F, et al. Assessment of Outdoor Air Temperature with Different Shaded Area within an Urban University Campus in Hot-Humid Climate Sustainability. 2020,12,5741.
65. Rafterya P, Fizer J, Chen W, He Y, Zhang H, Arens E, et al. Ceiling fans: Predicting indoor air speeds based on full scale laboratory measurements. Building and Environment. 2019,115,210-223.
66. Zaki SA, Othman N, Syahidah S, Yakub F, Muhammad-Sukki F, et al. Effects of urban morphology on microclimate parameters in an urban university campus. Sustainability. 2020,12(7),2962.
67. Lau KKL, Ren C, Ng JHE, Numerical modelling of mean radiant temperature in high-density sub-tropical urban environment. Energy and Buildings, 2016,114,80-86.
68. Subramanian R, Tunçer B, Binder A. Thermal Comfort Based Performance Appraisal of Covered Walkways in Singapore. in 24th CAADRIA Conference, New Zealand. 2019.
69. Liu H, Lim JY, Thet BWH, Lai PY, Koh WS. Evaluating the impact of tree morphologies and planting densities on outdoor thermal comfort in tropical residential precincts in Singapore. Building and Environment. 2022,221,109268.
70. Zaki SA, Toh HJ, Yakub F, Saudi ASM. Effects of roadside trees and road orientation on thermal environment in a tropical City. Sustainability. 2020,12,1053.
71. Vít V, Kopp J. Typology of Outdoor Seating Areas of Restaurants Based on Factors Influencing Their Thermal Comfort, A Case Study of Pilsen City Centre, Czechia. Journal of Settlements and Spatial Planning. 2019,10(2),131-142.

PLoS One / Ref. No.: PONE-D-22-17882

Title: Outdoor environmental comfort evaluation for retail planning in a tropical business district using Integrated Environmental Modeller

Author: Po-Yen Lai, Wee Shing Koh, Harish Gopalan, Huizhe Liu, Dias Leong, Hyosoo Lee, Johnathan Goh, Jiun Yeu Lim, Jacob Ang, Gibert Peh, Gilbert Cher, Cheng Hui Eng, Jia Li Goh, Edmund Tan, James Tan

---

Dear Editor,

Thanks for your response. We also would like to thank the reviewers for their valuable comments. Our point-by-point replies for the journal requirements and reviewers' comments are shown as follows. The revised manuscript is resubmitted. Please consider for the publication on PLOS ONE.

Reply to Journal requirements

C1	Please ensure that your manuscript meets PLOS ONE's style requirements, including those for file naming. The PLOS ONE style templates can be found at https://journals.plos.org/plosone/s/file?id=wjVg/PLOSOne_formatting_sample_main_body.pdf and https://journals.plos.org/plosone/s/file?id=ba62/PLOSOne_formatting_sample_title_authors_affiliations.pdf
R1	Thanks for the suggestion. We've applied the format of the manuscript in accordance with the requirements of the journal strictly, including those for file naming.
C2	a) Please note that PLOS journals require authors to make all data necessary to replicate their study's findings publicly available without restriction at the time of publication. Please see our Data Availability policy at https://journals.plos.org/plosone/s/data-availability. As such, please make the full specific dataset used in this study available by either A) uploading the full dataset as supplementary information files, or B) including a URL link in your Data Availability Statement and Methods section to where the full dataset can be accessed.<br>b) Additionally, please note that PLOS ONE has specific guidelines on code sharing for submissions in which author-generated code underpins the findings in the manuscript. In these cases, all author-generated code must be made available without restrictions upon publication of the work. Please review our guidelines at https://journals.plos.org/plosone/s/materials-and-software-sharing#loc-sharing-code and ensure that your code is shared in a way that follows best practice and facilitates reproducibility and reuse.
R2	a) We want to change our description of "Data Availability" as:<br>"All the simulation conditions were listed in the manuscript. The CAD data used in simulations will be made available at the A*STAR Open Access Repository (https://oar.a-star.edu.sg/) upon acceptance of the manuscript. Registration of an authenticated access account at the A*STAR Open Access Repository site is required. The CAD data will be made freely available if the user is going to use the CAD data for the sole purpose to reproduce the results presented in this paper. Permission has to be sought if the CAD data is going to be used for any other purposes."<br>b) The software used in the manuscript has been licensed by Wizvision Pte Ltd (https://www.wizvision.com/contact-us/). Interested users purchase the Integrated Environmental Modeller software from the distributor.
C3	Thank you for stating the following in the Acknowledgments Section of your manuscript:<br>"This research is supported by A*STAR under its URBAN & GREENTECH OFFICE (UGTO) CONSULTANCY FUND (Grant No.: UGTO/CF/01). This development work was undertaken as a part of the project "Modeling of Air Flow,

	Thermal and Chemical Gas Dispersion Towards Next Generation Port (Tuas Maritime Hub)", funded by the Singapore Maritime Institute (SMI-2016-MA-04). The conclusions put forward reflect the views of the author alone, and not necessarily those of the funding institution"<br>We note that you have provided funding information that is not currently declared in your Funding Statement. However, funding information should not appear in the Acknowledgments section or other areas of your manuscript. We will only publish funding information present in the Funding Statement section of the online submission form.<br>Please remove any funding-related text from the manuscript and let us know how you would like to update your Funding Statement. Currently, your Funding Statement reads as follows:<br>"This research is supported by A*STAR under its URBAN & GREENTECH OFFICE (UGTO) CONSULTANCY FUND (Grant No.: UGTO/CF/01). This development work was undertaken as a part of the project “Modeling of Air Flow, Thermal and Chemical Gas Dispersion Towards Next Generation Port (Tuas Maritime Hub)", funded by the Singapore Maritime Institute (SMI-2016-MA-04). The conclusions put forward reflect the views of the author alone, and not necessarily those of the funding institution. The funders had no role in study design, data collection and analysis, decision to publish, or preparation of the manuscript.<br>Please include your amended statements within your cover letter; we will change the online submission form on your behalf.
R3	Thanks for the comment. In this case, we removed the “Acknowledgments section” in the manuscript and we confirmed that the current Funding Statement reads as follows: “This development work was undertaken as a part of the project “Modeling of Air Flow, Thermal and Chemical Gas Dispersion Towards Next Generation Port (Tuas Maritime Hub)", funded by the Singapore Maritime Institute (SMI-2016-MA-04). The conclusions put forward reflect the views of the author alone, and not necessarily those of the funding institution. The funders had no role in study design, data collection and analysis, decision to publish, or preparation of the manuscript.” is correct.
C4	We note that Figure 1 in your submission contain [map/satellite] images which may be copyrighted. All PLOS content is published under the Creative Commons Attribution License (CC BY 4.0), which means that the manuscript, images, and Supporting Information files will be freely available online, and any third party is permitted to access, download, copy, distribute, and use these materials in any way, even commercially, with proper attribution. For these reasons, we cannot publish previously copyrighted maps or satellite images created using proprietary data, such as Google software (Google Maps, Street View, and Earth). For more information, see our copyright guidelines: http://journals.plos.org/plosone/s/licenses-and-copyright.<br>We require you to either (a) present written permission from the copyright holder to publish these figures specifically under the CC BY 4.0 license, or (b) remove the figures from your submission:<br>a. You may seek permission from the original copyright holder of Figure 1 to publish the content specifically under the CC BY 4.0 license.<br>We recommend that you contact the original copyright holder with the Content Permission Form (http://journals.plos.org/plosone/s/file?id=7c09/content-permission-form.pdf) and the following text:<br>“I request permission for the open-access journal PLOS ONE to publish XXX under the Creative Commons Attribution License (CCAL) CC BY 4.0 (http://creativecommons.org/licenses/by/4.0/). Please be aware that this license allows unrestricted use and distribution, even commercially, by third parties. Please reply and provide explicit written permission to publish XXX under a CC BY license and complete the attached form.”<br>Please upload the completed Content Permission Form or other proof of granted permissions as an "Other" file with your submission.<br>In the figure caption of the copyrighted figure, please include the following text: “Reprinted from [ref] under a CC BY

license, with permission from [name of publisher], original copyright [original copyright year].”

b. If you are unable to obtain permission from the original copyright holder to publish these figures under the CC BY 4.0 license or if the copyright holder’s requirements are incompatible with the CC BY 4.0 license, please either i) remove the figure or ii) supply a replacement figure that complies with the CC BY 4.0 license. Please check copyright information on all replacement figures and update the figure caption with source information. If applicable, please specify in the figure caption text when a figure is similar but not identical to the original image and is therefore for illustrative purposes only.

The following resources for replacing copyrighted map figures may be helpful:

USGS National Map Viewer (public domain): http://viewer.nationalmap.gov/viewer/

The Gateway to Astronaut Photography of Earth (public domain): http://eol.jsc.nasa.gov/sseop/clickmap/

Maps at the CIA (public domain): https://www.cia.gov/library/publications/the-world-factbook/index.html and https://www.cia.gov/library/publications/cia-maps-publications/index.html

NASA Earth Observatory (public domain): http://earthobservatory.nasa.gov/

Landsat: http://landsat.visibleearth.nasa.gov/

USGS EROS (Earth Resources Observatory and Science (EROS) Center) (public domain): http://eros.usgs.gov/#

Natural Earth (public domain): http://www.naturalearthdata.com/

R4 Thanks for pointing this out. We combined the **Figure 1(a)** and **(b)** and remove the copyrighted [map/satellite] images in **Figure 1(a)**. The amended **Figure 1** and the caption are as follows

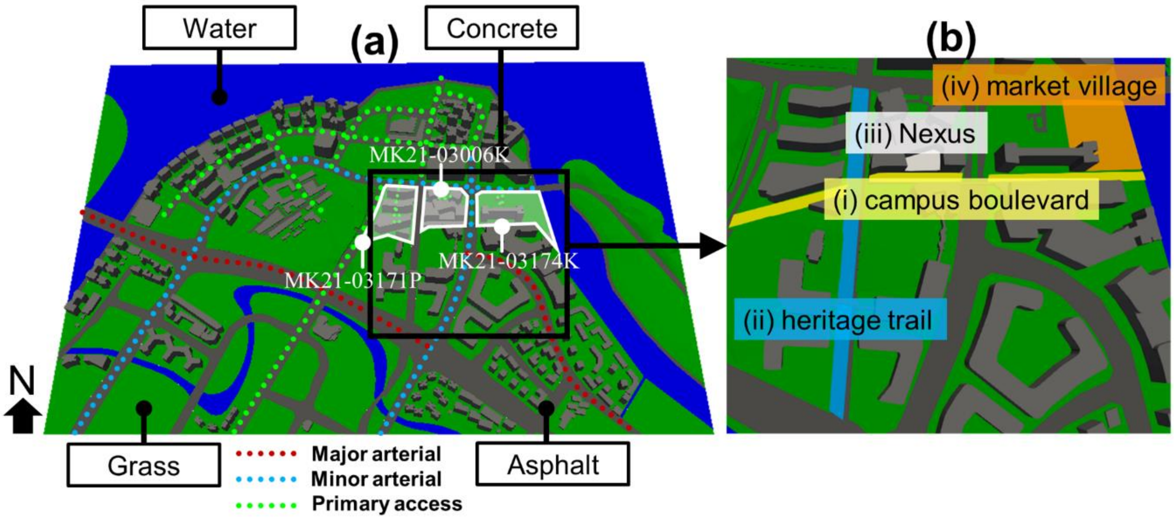

Fig 1. (a) The schematic illustration of the studied urban district in the north-east region of Singapore, including the material assignment of infrastructures and the categorization of traffic noise sources from main roads. White portions are the planned business park with the specific mukim lot numbers MK21-03171P, MK21-03006K and MK21-03174K. **(b)** The zoom-in feature of the study sites with high pedestrian traffic for the evaluation of comfort factors: (i) campus boulevard, (ii) heritage trail, (iii) Nexus, and (iv) market village.

The figure number has been updated accordingly in corresponding context. (Please see the document “Revised Manuscript with Track Changes” or “Manuscript”.)

C5 Regarding the potential copyright of figures 1, 5, 6, 7, and 8c please respond the following prompts:

A. For the above figures, were any shapefiles or basemaps obtained? If so, where were they obtained? Please provide any relevant links as well as any relevant copyright information.

B. For the above figures, were any external data utilized in the creation of these figures? If so, where were they obtained?

	Please inform us if the data was collected or produced by the authors. Please also provide any relevant links as well as any relevant copyright information.<br>C. Was any software (e.g. ArcGIS) used in the creation of these figures?
R5	A. No basemaps or shapefiles were used.<br>B. All the geometries were generated by the authors.<br>C. Commercial software, Revit and Rhino, were used to generate, report, and export the geometries for simulations. Simulations results were plotted with the open-source software, Paraview.

Reply to Reviewer #1's comments

C1	Include all the keywords inside the Abstract. "Tropical urban district", "Thermal comfort index", "Acoustic comfort index", "Environmental evaluation" and "retail planning" are not in the abstract.
R1	We thank the reviewer for the comment. The keywords are slightly revised as 「Retail planning, Integrated Environmental Modeller (IEM), Thermal comfort, Acoustic comfort, Tropical urban district」 and added into Abstract as follows.<br>Ln 16: 「…for a planned business urban district in Singapore for **retail planning** using…」<br>Ln 18: 「…called the **Integrated Environmental Modeller** (IEM). 」<br>Ln 22: 「…**thermal** and **acoustic comfort** acceptability indicators. 」<br>Ln 38-39: 「people working in or visiting the **tropical urban district**. 」
C2	Since coupled wind-thermal is one of main research, if possible the author might to explain more on definition or background what is coupled wind-thermal?
R2	The "wind-thermal" here is defined as the interaction between wind dynamics and heat transfer on the building or ground surfaces in the urban environment. "coupled" in the paper is mainly used to represent the connection between "wind-thermal model" and "noise propagation". For example, in the Ln 68「…coupled wind-thermal and noise propagation…」 To make it clearer, we add the definition of "wind-thermal" in the Ln 65-67 「in which the core physical model is the wind-thermal interaction between wind dynamics and heat transfer on building and ground surfaces in the urban environment. 」 and remove "coupled" in Ln 235.
C3	The author claim there is no studies on the coupled solar-wind-noise simulations, however there is model developed and used to study for this case? Seems like contradict. (on line 67 – 71).
R3	Thanks for pointing this out. The references [12, 21, 22, 23] are the different modules in IEM which was published for each physical schemes (i.e., solar radiation [12], wind dynamics [22, 23], and noise propagation [21]). The results of coupled solar-wind-noise simulations are not published yet.<br>To avoid misleading the readers, we amend the context and include few more current references on Ln 76-81 as 「… Integrated Environmental Modeller (IEM), has been developed to conduct coupled solar-wind-noise simulations in Singapore at different spatial scales from individual buildings to districts and regions. The IEM framework integrates environmental models, including solar radiation [12], wind dynamics [23, 24], and noise propagation [22], to captures the correlation between the physical models in simulations…」
C4	Line 442, reference defined the thresholds for thermal and acoustic comfort? Same goes to threshold for PTA and PNA.
R4	The comfort thresholds are not universal but are defined for different purposes and target matrices. We listed down few references, in which authors mentioned about "thermal comfort threshold", below for instance:<br>[Ref 4-1] Atmosphere 2018, 9, 108<br>[Ref 4-2] Procedia Engineering 2015, 2125-2131, 121<br>[Ref 4-3] Lancet Planet Health 2022, e301-e309, 6<br>In this work, we adopted the majority threshold for thermal and acoustic comfort, i.e., PTA = 0.5 and PNA = 0.5, to

determine whether majority of pedestrians accepts the thermal and acoustic conditions of the specific locations. The local thermal or acoustic comfort must be improved where PTA < 0.5 or PNA < 0.5. Then we claim the threshold used in this study is not universal in the latter context.

To make it clearer, we added the context on Ln 452-454 as 「…From different studies [Ref 4-1, Ref 4-2, Ref 4-3], other environmental comfort thresholds were introduced based on different environmental matrices for the specific purpose. 」

C5 Line 612, is there any reference to support author claim on blocking solar.

R5 Thanks for the comment. As referring [Ref 5-1, Ref 5-2, Ref 5-3, Ref 5-4], controlling solar radiation (blocking by shelter or tree canopy) can be seen as the most effective way to improve the outdoor thermal comfort. We added these references into the manuscript on Ln 681-682 as 「…This fact means that blocking solar irradiance is more efficient to improve $\overline{PTA}$ in open spaces that agree with the findings from [Ref 5-1, Ref 5-2, Ref 5-3, Ref 5-4]. …」

[Ref 5-1] Shashua-Bar, L., Ioannis, X.T., Milo, H. Passive cooling design options to ameliorate thermal comfort in urban streets of a Mediterranean climate (Athens) under hot summer conditions. Build. Environ. 2012, 57, 110–119.

[Ref 5-2] Cheung, P.K. & Jim, C.Y. Comparing the cooling effects of a tree and a concrete shelter using PET and UTCI. Build. Environ. 2018, 130, 49–61.

[Ref 5-3] Nasrollahi, N., Ghosouri, A., Khodakarami, J., Taleghani, M. Heat-Mitigation Strategies to Improve Pedestrian Thermal Comfort in Urban Environments: A Review. Sustainability 2020, 12, 10000.

[Ref 5-4] Assessment of outdoor air temperature with different shaded area within an urban university campus in hot-humid climate. Open Access. Zaki, S.A., Syahidah, S.W., Shahidan, M.F., (...), Hassan, M.Z., Daud, M.Y.M. 2020. Sustainability (Switzerland). 12(14), 5741, pp. 1-24

C6 Maybe the author can compile results from Figure 9, 10 ,11 and 12 in Table.

R6 Thanks for the comment. We added Table 4 that summarize the results from Figures 9, 10 ,11 and 12 as follows:

Table 4. The table summarizes the conditions to achieve $\overline{PTA}$ = 0.5 from **Error! Reference source not found.**(d), 10(d), 10(e), 11(d), 12(d), and 12(e). In each cell, the upper panel is the required shade percentage, and the upper panel is the required increasing wind speed. The cells in green represent that $\overline{PTA}$ ≥ 0.5 can be naturally achieved. The cells in red represent that cannot be achieved by only cannot be achieved by only increasing the wind speed in a reasonable range (< 10 m/s).

		campus boulevard<br>site (i)	heritage trail<br>site (ii)	Nexus<br>site (iii)	market village<br>site (iv)
sunny day	10:30	42 %<br>3.7 m/s	32 %<br>2.6 m/s		29 %<br>1.8 m/s
	13:30	68%<br>>10 m/s	68 %<br>>10 m/s	4.2 %<br>0.2 m/s	54 %<br>>10 m/s
cloudy day	10:30		6.7 %<br>0.3 m/s		17 %<br>0.8 m/s
	13:30		21 %<br>1.1 m/s		27.5 %<br>1.8 m/s

The corresponding context was added on Ln 615-617: 「…The overall conditions to achieve $\overline{PTA}$ = 0.5 from **Error! Reference source not found.**(d), 10(d), 10(e), 11(d), 12(d), and 12(e) are summarized in Table 4. 」 and on Ln 743-744: 「…According to Table 4 (the results from **Error! Reference source not found.**(d), **Error! Reference source not**

	**found.**(d), **Error! Reference source not found.**(e), **Error! Reference source not found.**(d), **Error! Reference source not found.**(d), and **Error! Reference source not found.**(e)) …」
C7	Here are several references the author might consider looking at:<br>[Ref 1] Field study of pedestrians' comfort temperatures under outdoor and semi-outdoor conditions in Malaysian university campuses Othman, N.E., Zaki, S.A., Rijal, H.B., Ahmad, N.H., Razak, A.A. 2021. International Journal of Biometeorology. 65(4), pp. 453-477<br>[Ref 2] Analysis of urban morphological effect on the microclimate of the urban residential area of Kampung Baru in Kuala Lumpur using a geospatial approach. Open Access. Zaki, S.A., Azid, N.S., Shahidan, M.F., (...), Ali, M.S.M., Yakub, F. 2020. Sustainability (Switzerland). 12(18),7301<br>[Ref 3] Assessment of outdoor air temperature with different shaded area within an urban university campus in hot-humid climate. Open Access. Zaki, S.A., Syahidah, S.W., Shahidan, M.F., (...), Hassan, M.Z., Daud, M.Y.M. 2020. Sustainability (Switzerland). 12(14),5741, pp. 1-24<br>[Ref 4] Effects of urban morphology on microclimate parameters in an urban university campus. Open Access. Zaki, S.A., Othman, N.E., Syahidah, S.W., (...), Shahidan, M.F., Saudi, A.S.M. 2020. Sustainability (Switzerland). 12(7), 2962<br>[Ref 5] Effects of roadside trees and road orientation on thermal environment in a tropical City. Open Access. Zaki, S.A., Toh, H.J., Yakub, F., (...), Ardila-Rey, J.A., Muhammad-Sukki, F. 2020. Sustainability (Switzerland). 12(3),1053
R7	Thanks for introducing the relevant references. We included four of them as follows:<br>[Ref 1] was cited on Ln 91 as: 「…To realize a fair thermal comfort assessment in a tropical climate, it is better to consider the results from local field studies in the existing thermal comfort index, e.g., [Ref 1, 33].…」<br>[Ref 3] was cited on Ln 682 as 「…This fact means that blocking solar irradiance is more efficient to improve $\overline{PTA}$ in open spaces that agree with the findings from [61, 62, 63, Ref 3]. …」<br>[Ref 4] was cited on Ln 719 as 「…The building shadows can directly improve the thermal comfort [Ref 4, 67]. …」<br>[Ref 5] was cited on Ln 722 as 「…and planting street trees with overhead coverage [54, 69, Ref 5]. …」
C8	The comments about the research conditions:<br>a) The reason of author choosing those specific research place?<br>b) The author might consider to include yearly temperature to support author chosen day.<br>c) Table 3, how the author choose the direction?<br>d) Line 318, reference on choosing dB value?<br>e) Line 366, the reason of choosing this specific initial temperature?<br>f) Line 667, the author can include the reasoning using this ideal condition.
R8	Thanks for the comments. Our responses are below:<br>a) The chosen place in study is a real planned district in Singapore (as mentioned in Abstract: Ln 16). The research results can help the planner to evaluate outdoor thermal and acoustic comfort. The selected studied sites in this paper are either the main pedestrian thoroughfares or gathering areas for outdoor community building and events with potential high pedestrian footfall where outdoor retail options are planned along or within these sites (see the context in Sec 2.1.1 Ln 149-157).<br>b) According to the Singapore's records of climate station in the past 30 years [39], the mean daily maximum air temperature is 32 ºC as defined "hot". To make it clear, we add the definition of hot day on Ln 200-202: 「…the hottest period (i.e., from March to June in which the air temperature is commonly higher than the mean daily maximum temperature of 32 ºC based on the climatological reference period between 1991 to 2020 [39]) …」<br>c) We adopted the BCA Green Mark's prevailing wind conditions [41], including mean speed and mean direction for Singapore, is based on the National Environment Agency's 18-year data at a reference height of 15.0 m. To make

<table>
<tr><td></td><td>Table 3 clearer, we amended the caption and table as follows:

Table 3. Climate conditions of wind applied to this paper. The expected values follow the Building and Construction Authority’s (BCA) Green Mark [41] at the reference height of 15 m based on the National Environment Agency’s 18-year data in Singapore.

<table>
<tr><th>Date</th><th>mean speed (m/s)</th><th>mean direction</th></tr>
<tr><td>Equinox (sunny day)</td><td>2.9</td><td>South</td></tr>
<tr><td>Solstice (cloudy day)</td><td>2.8</td><td>North-East</td></tr>
</table>
d) The value of 65 dB is suggested by WHO for the acoustic health purpose. To make it clear, we amend the context and add the corresponding reference on Ln 321-323 as: 「…Considering that the noise levels >65 dB could endanger the acoustic heath in urban environment [5], we assume that residents perceive traffic and construction noise annoyance level similarly at 65 dB …」
e) We adopted the BCA Green Mark’s initial isothermal conditions of 30.0°C air temperatures to carry out the simulations. To make it clearer, we rewrote the corresponding context on Ln 373-374 as: 「…We adopted the initial isothermal conditions of 30.0°C air temperatures to carry out the simulations according to the BCA Green Mark standard [41].」
f) In this work, the reason for applying the ideal conditions is to propose the general parametric scope of thermal comfort improvement efficiently that can provide a quick guide for architects to plan the infrastructure and landscape combination during the scenario design stage. These ideal conditions are assumed that the solar radiation levels and wind speed have implicitly incorporated the effects of trees, shades, or outdoor fans. The exact details of surrounding environment, i.e., shades, trees and fans can be inversely designed based on the microclimate conditions, which is out of scope of this paper.
g) To make it clearer, we added the reason for ideal condition in the corresponding context on Ln 749-756 as: 「… Under the ideal conditions, we assumed that the solar radiation level and wind speed have implicitly incorporated the effects of trees, shades, or outdoor fans. The double parametric space in terms of solar radiation and wind speed provides a guideline for architects to quickly identify the optimal microclimate conditions for planning the infrastructure and landscape combination during the scenario design stage. The exact details of the surrounding environment, i.e., shades, trees and fans, can be inversely designed based on the optimal microclimate condition, which is out of the scope of this paper and requires further study. …」</td></tr>
<tr><td>C9</td><td>The comments for corrections:
a) Always start introduce the abbreviation first before use it. Line 64, “SOLWEIG” and “ENVI-met” not defined. Line 89, “WHO”. Line 104, “3D”.
b) Line 111, since author already defined IEM, just use IEM instead of full name.
c) Line 287, “Fk” referring for three things?
d) Figure 5, no arrow in (b).</td></tr>
<tr><td>R9</td><td>Thanks for the comments. Our responses are below:
a) We added “SOlar Long Wave Environmental Irradiance Geometry model (SOLWEIG)” in Ln 71-72, “World Health Organization (WHO)” in Ln 100, and “three dimensional (3D)” in Ln 115-116, respectively. ENVI-met is not the abbreviation but the name of the micro-climate software and the funded company.
b) We removed the full name of IEM in Ln 121.</td></tr>
</table>

	c) F_k is the angle factor form in k direction. To avoid misleading, we have rewritten the context in Ln 290-292: 「… Q_{Sk} (W/m^2) is the received shortwave radiation, $Q_{Lk}(W/m^2)$ is the received longwave radiation, and F_k is angle factor form in k direction…」<br>d) It's the typo. We corrected the caption in Figure 5: 「…The arrow in **(c)** denotes the wind direction.」<br>In addition, we also did the revisions in the manuscript as follows:<br>• "hourly average" to "hourly-averaged"<br>• Typo: we revised all "11:30" to "13:30"

Reply to Reviewer #2's comments

C1	This study does not include the novelty and significance of their research. This study is close to a technical report rather than a research paper. In addition, the acoustic comfort evaluation was simply conducted.
R1	We thank the reviewer to point out the comment that helps us to further improve the manuscript. We have carefully revised the **Abstract**, **Results and discussion**, and **Conclusion** sections to enhance the work's novelty and significance. Our bullet-point reply to this comment is addressed as follows:<br>• The novelty and significance of this research:<br>**Novelty:**<br>1. The first workflow of the solar-wind-noise coupled simulation rather than the conventional two-step solar-wind coupled simulations [12-15] is presented.<br>2. The spatial plots of environmental acceptability indicators to distinguish the different zones exposed to thermal or noise problems in the worst-case scenario are proposed. (in the revised paper)<br>3. The parametric contours of averaged thermal acceptability indicators for improving overall thermal comfort at the studied sites are proposed.<br>4. The guideline, considering thermal and acoustic comfort evaluation simultaneously for retail planning in a planned district, is given.<br>**Significance:**<br>1. The coupled physics (solar-wind-noise) simulations have been conducted for a planned business urban district in Singapore.<br>2. Using the simulation results, the spatial distribution of thermal and acoustic acceptability indicators can be divided into local zones with different comfort extents.<br>3. In the worst-case scenario, noise-affected zones are concentrated near the noise sources, and the thermal-affected zones are almost everywhere at the studied sites.<br>4. The area-averaged thermal acceptability indicators have been derived to quickly evaluate and improve the overall thermal comfort at each specific site for the high-level retail training.<br>5. According to the evaluation results, the work proposed the guideline of selecting the proper locality for outdoor retail planning, the strategy for improving outdoor environmental comfort, and excluding the difficult areas exposed thermal-noise problem if the thermal and acoustic comfort cannot be improved simultaneously.<br>• Acoustic comfort evaluation in this study:<br>We agree that the evaluation of acoustic comfort is simpler than thermal comfort in the previous manuscript. The area-averaged environmental acceptable indicators conveniently evaluate the overall thermal and acoustic comforts for the specific area. The overall acoustic comforts are more acceptable at each site than thermal comforts. However, the local acoustic comfort is always unacceptable in the region near the noise sources. The fact shows that spatial noise level variations should be considered in retail planning to define the uncomfortable acoustic zone. Therefore,

	we add the statistical error bar, extending from the averaged environmental acceptability indicators, to propose the most acceptable or unacceptable extent to each site's thermal and acoustic environmental conditions at different points in time. Moreover, we add the spatial plots of the distribution of environmental acceptability indicators to divide the area of each studied site into the zone affected by thermal or noise issues. According to the newly added contents, the complex regions encountering thermal and noise issues simultaneously are recommended to exclude outdoor retail activities if the thermal and acoustic issues cannot be improved simultaneously. This can further improve the evaluation and guideline for the retail planning for the planned district in Singapore. The corresponding content can be found in the revised manuscript's **Abstract**, **Results and discussion**, and **Conclusion** sections.
