# Supplementary material for: Outdoor environmental comfort evaluation for retail planning in a tropical business district using Integrated Environmental Modeller
Source: PLoS One. 2023 Mar 3;18(3):e0282106. doi: 10.1371/journal.pone.0282106 (PMC9983900; doi:10.1371/journal.pone.0282106)
Supplement: Attachment — Submitted filename: Response to Reviewers.pdf [file pone.0282106.s004.pdf]

PLoS One / Ref. No.: PONE-D-22-17882R1

Title: Outdoor environmental comfort evaluation for retail planning in a tropical business district using Integrated Environmental Modeller

Author: Po-Yen Lai, Wee Shing Koh, Harish Gopalan, Huizhe Liu, Dias Leong, Hyosoo Lee, Johnathan Goh, Jiun Yeu Lim, Jacob Ang, Gibert Peh, Gilbert Cher, Cheng Hui Eng, Jia Li Goh, Edmund Tan, James Tan

---

Dear Editor,

Thanks for your response. We also would like to thank the reviewers for their valuable comments in improving the manuscript. Our point-by-point replies to the journal requirements and reviewers' comments are as follows. The revised manuscript has been resubmitted. Please consider the revised manuscript for publication on PLOS ONE.

Reply to Journal requirements

C1	Please review your reference list to ensure that it is complete and correct. If you have cited papers that have been retracted, please include the rationale for doing so in the manuscript text, or remove these references and replace them with relevant current references. Any changes to the reference list should be mentioned in the rebuttal letter that accompanies your revised manuscript. If you need to cite a retracted article, indicate the article's retracted status in the References list and also include a citation and full reference for the retraction notice.
R1	We confirm that the reference list is complete and correct. One new reference has been added and addressed in R1 in Reply to Reviewer #1's comments.

Reply to Reviewer #1's comments

C1	The author answered all the questions and made the required changes. However, one of the suggestion paper is not included in this paper as follows:<br>[Ref 1] Analysis of urban morphological effect on the microclimate of the urban residential area of Kampung Baru in Kuala Lumpur using a geospatial approach. Open Access. Zaki, S.A., Azid, N.S., Shahidan, M.F., (...), Ali, M.S.M., Yakub, F. 2020. Sustainability (Switzerland). 12(18), 730
R1	Thanks for raising this point. We included this reference as follows:<br>[Ref 1] was cited on Ln 50 in the revised paper as: 「…This induces a higher urban air temperature than that in rural areas, producing thermal stress [2], i.e., urban heat island (UHI) [3, 4] which can be particularly observed in tropical environments [Ref 1]. …」<br>[Ref 1] Analysis of urban morphological effect on the microclimate of the urban residential area of Kampung Baru in Kuala Lumpur using a geospatial approach. Open Access. Zaki, S.A., Azid, N.S., Shahidan, M.F., (...), Ali, M.S.M., Yakub, F. 2020. Sustainability (Switzerland). 12(18), 730

Reply to Reviewer #3's comments

C1	Clarify if the IEM simulation results are validated in the study.
R1	Thanks for this comment. The physical models in the IEM solver have been validated in previous works for solar [Ref 1], wind [Ref 2, Ref 3], and noise-propagation [Ref 4] simulations under climate conditions in Singapore. This study aims to provide an environmental evaluation of a ***planned district*** in Singapore using IEM under TMY climate conditions for retail purposes. This district's construction is ongoing, so that no corresponding measurements can be done. That is why we added the validation works by on-site measurements in future works. To avoid misunderstanding, we added a sentence about the IEM validation into Sec 2.2.<br>On Ln 236-238 in the revised manuscript, we added the context as 「…These physical models in IEM have been validated for solar radiation [Ref 1], wind dynamics [Ref 2, Ref 3], and traffic-noise simulations [Ref 4] under the climate conditions in Singapore.…」<br>[Ref 1] Wang B, Koh WS, Liu H, Yik J, Bui VP. Simulation and validation of solar heat gain in real urban environments. Building and Environment. 2017,123,261-276.<br>[Ref 2] Wise D, Boppana V, Li K, Poh H, Effects of Minor Changes in the Wind Direction on Urban Flow Simulations. Sustainable Cities and Society. 2018,37,492-500.<br>[Ref 3] Gopalan H. Evaluation of Wray-Agarwal turbulence model for simulation of neutral and non-neutral

	atmospheric boundary layers. Journal of Wind Engineering and Industrial Aerodynamics. 2018,182, 322-329.<br>[Ref 4] Zhao WJ, Liu EX, Poh HJ, Wang B, Gao SP and Png CE. 3D traffic noise mapping using unstructured surface mesh representation of buildings and roads. Applied Acoustics. 2017,127,297-304.
C2	Please correct the typographical and grammatical errors in the manuscript.
R2	Thanks for the comments for corrections. Our responses are below:<br>• To make it clearer, we removed "mukim" on Ln 140 in the previous manuscript and rewrote the corresponding content as: 「…are referred to by their respective lot numbers. …」 on Ln 142 in the revised manuscript. Also, we removed "mukim" in the caption of **Fig 1** in the revised manuscript.<br>• We corrected the typo "metrological" on Ln 208 in the previous manuscript to "meteorological" on Ln 210 in this previous manuscript.<br>• To make it clearer, we rewrote 「"…, a more straightforward measure can be more useful in practice. Thus, operative temperature (T_O) was adopted in this study as the thermal index derived from air temperature, mean radiant temperature, and wind speed."」 on Ln 279-281 in the previous manuscript to 「"…, we proposed to use a compact thermal index, operative temperature (T_O). T_O can be derived from the direct measurement of air temperature using a thermometer, mean radiant temperature using a globe thermometer, and wind speed using an anemometer."」 on Ln 283-286 in the revised manuscript.<br>• We corrected "*PTA*" on Ln 543 in the previous manuscript to "*PNA*" on Ln 547 in the revised manuscript.<br>• We corrected "are almost none existed" on Ln 549 in the previous manuscript to "are almost nonexistent" on Ln 553 in the revised manuscript.<br>• We rewrote "<" on Ln 555 in the previous manuscript as "up to" on Ln 559 in the revised manuscript.<br>• We corrected "can be achievable" on Ln 557 in the previous manuscript to "is achievable" on Ln 561 in the revised manuscript.<br>• We corrected "PNA > 0.5" on Ln 581 in the previous manuscript to "PNA < 0.5" on Ln 586 in the revised manuscript.<br>• In the caption of **Table 4**, we omitted the repetition "cannot be achieved by only" in the previous manuscript and rewrote "< 10 m/s" as "of up to 10 m/s" in the revised manuscript.<br>• To make it clearer, we rewrote "The site with more surrounding buildings, which act as acoustic barriers, has higher PNA. Thus, the site (iii)…" on Ln 633-634 in the previous manuscript to "The site with a greater number of surrounding buildings, which act as acoustic barriers, has a higher PNA. For instance, the site (iii)…" on Ln 638-639 in the revised manuscript.<br>• We corrected "smaller than" on Ln 685 in the previous manuscript to "less than" on Ln 690 in the revised manuscript.<br>• To make it clearer, we rewrote 「"At site (i) (campus boulevard) where is a thoroughfare with buildings on either side, $\overline{PTA}$ can be expected to improve by installing pop-up shopping areas next…"」 on Ln 714 in the previous manuscript to 「"The site (i) (campus boulevard) is a thoroughfare with buildings on either side. The $\overline{PTA}$ along this thoroughfare can be improved by installing pop-up shopping areas next…"」 on Ln 718-719 in the revised manuscript.<br>• We rewrote "lacking" on Ln 718 in the previous manuscript as "the lack of" on Ln 723 in the revised manuscript.」<br>• We rewrote 「"On the other hand, outdoor activities become inappropriate in the area exposed the traffic noise, i.e., purple zones"」 on Ln 738-739 in the previous manuscript as 「"In addition, outdoor retail activities might possibly affect people's health in the area with a high-level thermal and noise exposure, i.e., purple zones"」 on Ln 742-744 in the revised manuscript.<br>• We rewrote 「"Considering only a single strategy is adopted, blocking solar irradiance is efficient to …"」 on Ln 802-803 in the previous manuscript as 「"Blocking solar irradiance is effective to …"」 on Ln 796 in the revised manuscript.<br>• We rewrote 「"PTA rises from …"」 on Ln 802-803 in the previous manuscript as 「"The values of PTA rise from …"」 on Ln 797 in the revised manuscript.
C3	Based on the study, certain generic recommendations can be provided in the discussion / conclusion.
R3	Our responses are summarized in R4.
C4	In the Conclusion, first few sentences are based on the methodology adopted which could be removed and it can begin with the specific recommendations based on the findings of the study and shall conclude with generic suggestion for future studies.
R4	Thanks for the suggestion. Our responses are below:<br>• We removed the according sentences in the Conclusion.<br>• We rewrote the content on Ln 816-827 in the previous manuscript by adding the specific recommendations based on the findings of the study on Ln 810-826 in the revised manuscript: 「…With the aid of thermal and acoustic comfort evaluation results derived from IEM, specific outdoor retail space design recommendations for each site were suggested. The site (i) could be a naturally ventilated shopping street with awnings or overhangs extended out from the buildings to enhance the thermal comfort for visitor queuing. Retail spaces are not recommended for the site (ii), especially for the northern section with poor thermal and acoustic comfort. They could function as a pedestrian-only path with roadside trees or extensive shelters to improve the thermal comfort as a much higher amount of shading as compared to the site (i). The site (ii) is required to achieve the same amount of thermal acceptability from 50% of the visitors or pedestrians walking along this pedestrian thoroughfare. The site (iii)

	benefits from the shade natural noise barrier from three sides through the clever design of the building architecture. Thus, this makes this site an ideal location for long-term al fresco dining and/or as a podium for small-scale community events. The site (iv) exhibits thermal and acoustic comfort issues, and only short-term retail activities and community events with plenty of shading, such as the weekend bazaar held under tentages, are recommended. If long-term retail spaces are to be situated at this site, the space must be air-conditioned to mitigate long-term thermal stress and noise impact on the staff working here.⋯」<br>• We rewrote the content on Ln 827-837 in the previous manuscript by adding the generic recommendations and future works based on the findings of the study on Ln 826-848 in the revised manuscript: 「⋯Our findings show that the design of the outdoor retail spaces for a tropical urban district is generally divided into three types of spaces: (1) outdoor spaces with no thermal and acoustic comfort issues (blue zone), (2) spaces with thermal comfort issues only (red zone), and (3) spaces with significant thermal and acoustic comfort issues (purple zone). The general retail options recommended for blue zones are al fresco dining or outdoor barbeque restaurants. For the red zones, naturally ventilated retail spaces, shopping streets with solar-blocking standalone shelters, awnings or overhangs from buildings are preferred. With the expected high traffic noise level and thermally unacceptable environment in the purple zones, only short-term naturally ventilated retail spaces, e.g., weekend bazaars or short-term pop-up kiosks, should be considered. This will avoid extended periods of high-level noise exposure, even though the thermal comfort could be improved with shading infrastructure or active mechanical ventilation. However, if retail stores or restaurants must be included in the purple zones, these spaces (purple zone) must be enclosed and air-conditioned to keep out both traffic noise and ambient heat to minimize the health risk posed to the staff and regular patrons at these retail spaces with prolonged exposure. Furthermore, this study reveals that both thermal and acoustic comfort, derived from IEM solar-wind-noise coupled mapping, presents a previously under-explored dimension of retail space planning. Potential future works include validating IEM results against on-site measurements and evaluating possible landscape and infrastructure combinations that could help mitigate outdoor thermal and acoustic comfort issues locally (e.g., trees with shelter walkaways, green walls with outdoor ventilation fans, etc.).」
